# AI-Enhanced Prediction of Aortic Stenosis Progression: Insights From the PROGRESSA Study

**DOI:** 10.1016/j.jacadv.2024.101234

**Published:** 2024-09-11

**Authors:** Melissa Sanabria, Lionel Tastet, Simon Pelletier, Mickael Leclercq, Louis Ohl, Lara Hermann, Pierre-Alexandre Mattei, Frederic Precioso, Nancy Coté, Philippe Pibarot, Arnaud Droit

**Affiliations:** a Centre hospitalier universitaire de Québec – Université Laval, Québec City, Québec, Canada; b Université Côte d'Azur, Inria, CNRS, I3S, Maasai, Sophia Antipolis, France; c Institut universitaire de cardiologie et de pneumologie de Québec - Université Laval, Québec City, Québec, Canada; d Cardiovascular Division, Department of Medicine, University of California, San Francisco, California, USA

**Keywords:** aortic stenosis, deep learning, machine learning, risk prediction

## Abstract

**Background:**

Aortic valve stenosis (AS) is a progressive chronic disease with progression rates that vary in patients and therefore difficult to predict.

**Objectives:**

The aim of this study was to predict the progression of AS using comprehensive and longitudinal patient data.

**Methods:**

Machine and deep learning algorithms were trained on a data set of 303 patients enrolled in the PROGRESSA (Metabolic Determinants of the Progression of Aortic Stenosis) study who underwent clinical and echocardiographic follow-up on an annual basis. Performance of the models was measured to predict disease progression over long (next 5 years) and short (next 2 years) terms and was compared to a standard clinical model with usually used features in clinical settings based on logistic regression.

**Results:**

For each annual follow-up visit including baseline, we trained various supervised learning algorithms in predicting disease progression at 2- and 5-year terms. At both terms, LightGBM consistently outperformed other models with the highest average area under curves across patient visits (0.85 at 2 years, 0.83 at 5 years). Recurrent neural network-based models (Gated Recurrent Unit and Long Short-Term Memory) and XGBoost also demonstrated strong predictive capabilities, while the clinical model showed the lowest performance.

**Conclusions:**

This study demonstrates how an artificial intelligence-guided approach in clinical routine could help enhance risk stratification of AS. It presents models based on multisource comprehensive data to predict disease progression and clinical outcomes in patients with mild-to-moderate AS at baseline.

Aortic valve stenosis (AS) is a chronic progressive disease and is the most prevalent valvular heart disease in high-income countries.^1^ In North America, it is estimated that 3 million individuals are affected by AS, and its prevalence as well as its ensuing health and economic burden are expected to increase substantially over the next decades.^2^ Currently surgical aortic valve replacement (SAVR) and transcatheter aortic valve implantation (TAVI) remain the only therapeutic options.^3^^,^^4^ Echocardiography is the primary imaging modality for diagnosis, assessment of hemodynamic severity and progression rate of AS, which determine the timing of intervention.^3^^,^^4^ However, predicting the hemodynamic progression of AS remains challenging, as disease progression varies widely from one patient to another. Consequently, there is an urgent need to develop novel approaches to enhance prediction of AS progression and risk stratification to optimize the timing of follow-up and intervention in AS.

Artificial intelligence (AI) algorithms have been previously used for various tasks in cardiology including risk prediction and optimization of decision-making.^5^^,^^6^ Several studies reported the applicability and accuracy of such algorithms to detect AS in various settings.^7^, ^8^, ^9^ In addition, we previously demonstrated the usefulness of a machine learning pipeline that integrates a few echocardiographic parameters to improve risk stratification of AS.^10^ However, the ability of such algorithms to predict AS progression has not yet been explored. Recurrent neural networks (RNN), usually implemented for problems where temporal sequences are involved, could provide a promising approach to apply deep learning for predicting disease progression over time, relevant for therapeutic decision-making. Additionally, tree-based algorithms like XGBoost and LightGBM, which also have demonstrated strong performance with temporal data,^11^^,^^12^ may deliver accurate predictions in our context.

In the present study, we integrated comprehensive clinical and echocardiographic data from patients with years of follow-up into a RNN and tree-based algorithms to predict AS progression and then improve the risk prediction of clinical events compared to well-known cardiovascular risk factors and standard echocardiographic parameters.

## Methods

### Study sample

Patients with at least mild AS (ie peak aortic jet velocity [V_peak_] ≥2.0 m/s), with no or mild symptoms, were prospectively recruited in the PROGRESSA (Metabolic Determinants of the Progression of Aortic Stenosis) study (NCT01679431), started in 2005 and ongoing, at the Institut universitaire de cardiologie et de pneumologie de Québec–Université Laval and underwent Doppler echocardiography annually. The purpose and design of the PROGRESSA study have been previously described.^13^^,^^14^ Patients were excluded if they had symptomatic AS, moderate or greater aortic regurgitation, or mitral valve disease (stenosis or regurgitation), left ventricular ejection fraction < 50%, and if they were pregnant or lactating. None of the patients had an indication for aortic valve intervention at baseline. Among the 351 patients recruited until January 2020, 303 patients had follow-up visit(s) with comprehensive clinical and imaging data for the present subanalysis of the PROGRESSA study. No patients were lost in follow-up after enrollment. The study was approved by the Ethics Committee of the Institut universitaire de cardiologie et de pneumologie de Québec–Université Laval (Québec, CANADA) and all patients signed a written informed consent at the time of enrollment.

### Clinical data

Clinical data included age, sex, body surface area (BSA), body mass index (BMI), and functional status (ie, NYHA functional classification) at the time of index echocardiography. Clinical comorbidities were documented at baseline visit and included hypertension, diabetes mellitus, history of smoking, coronary artery disease, atrial fibrillation, and other clinical risk factors. The clinical identification of patients with features of the metabolic syndrome was assessed as previously described.^14^

### Laboratory data

From fasting blood samples, plasma levels of glucose, creatinine, N-terminal pro B-type natriuretic peptide, high-sensitivity troponin T, standard lipid profile, apolipoprotein B, apolipoprotein A-I, and standard hematology profile were measured using automated techniques standardized with the Canadian reference laboratory.

### Echocardiographic data

Comprehensive Doppler echocardiography exams were conducted by the same team of sonographers and cardiologists using commercially available ultrasound systems; images were analyzed in a core laboratory by experienced readers, as previously described.^13^^,^^15^ The aortic valve phenotype (ie, bicuspid versus tricuspid) was recorded. Stroke volume was calculated by multiplying the LV outflow tract area by the flow velocity-time integral and was indexed to BSA (stroke volume index). The Doppler-echocardiographic parameters of AS severity included V_peak_, mean pressure gradient, and aortic valve area (AVA) calculated by the standard continuity equation and indexed to BSA, as recommended by guidelines.^16^ AS severity was primarily classified mild (V_peak_ 2.0-2.9 m/s or gradient <20 mm Hg and AVA >1.5 cm^2^), moderate (V_peak_ 3.0-3.9 m/s, gradient 20-39 mm Hg or AVA 1.0-1.5 cm^2^), high-gradient severe (V_peak_ ≥4.0 m/s or gradient ≥40 mm Hg), or low-gradient severe (V_peak_ <4.0 or gradient <40 mm Hg, and AVA≤1.0 cm^2^) AS were classified as severe. Patients with V_peak_ <3.0 m/s but AVA <1.5 cm^2^ were classified as moderate AS. LV dimensions and mass were measured according to the recommendations of the American Society of Echocardiography and European Association of Cardiovascular Imaging.^17^ LV ejection fraction was measured with the use of the biplane Simpson method. E- and A-wave peak velocities from the mitral inflow profile were measured using pulsed wave Doppler according to guidelines.^18^ Early diastolic velocity of the mitral annulus (e’) was obtained by Doppler tissue imaging at the lateral and septal aspects of the annulus and then averaged for each patient. Total E/e’ ratio was then calculated.

### Data preprocessing

Our database contains 127 measures (so-called features) from 3 different sources: clinical, laboratory, and echocardiographic data (Table 1). None of these features contained more than 5% missing data. The categorical features (ie, features having a finite set of possible values) were transformed to one-hot encoding vectors, ie, categorical values become features and values are binarized with 0 and 1 in each new feature. After this transformation, our data set presented 176 features. Finally, each feature was normalized between −1 and 1 and the missing values were replaced by −1 to indicate the absence of data, clearly distinguishing them from the other values, hence helping the model differentiate between actual data and missing values.^19^

**Table 1:** Baseline Characteristics of the Study Sample

	All Patients (N = 303)	Clinical Events (n = 169)
Clinical data		
Age, y	64 ± 14	67 ± 11
Female	85 (28)	41 (24)
Body surface area, m^2^	1.89 ± 0.20	1.90 ± 0.19
Body mass index, kg/m^2^	29 ± 5	29 ± 4
NYHA functional class I or II	298 (99)	164 (98)
Systolic blood pressure, mm Hg	137 ± 18	139 ± 17
Diastolic blood pressure, mm Hg	77 ± 9	77 ± 8
Hypertension	209 (69)	127 (75)
Diabetes mellitus	77 (25)	46 (27)
Metabolic syndrome	62 (21)	32 (19)
History of smoking	183 (60)	107 (63)
Coronary artery disease	90 (30)	56 (33)
History of atrial fibrillation	39 (13)	22 (13)
Medication data		
ACE inhibitors	82 (27)	53 (31)
ARBs	91 (30)	53 (31)
Beta-blockers	94 (31)	55 (33)
Lipid-lowering agents	200 (66)	112 (66)
Anticoagulants	21 (7)	12 (7)
Laboratory data		
LDL-C, mmol/L	2.17 (1.75-2.73)	2.20 (1.80-2.77)
apoB, g/L	0.80 (0.70-0.99)	0.81 (0.73-1.00)
Triglycerides, mmol/L	1.27 (0.90-1.74)	1.30 (0.95-1.77)
Fasting glucose, mmol/L	5.4 (5.0-6.1)	5.4 (5.0-6.1)
Creatinine, μmol/L	81 (70-94)	83 (73-95)
NT-proBNP, pg/mL	83 (41-204)	108 (50-236)
High-sensitivity troponin T, ng/L	8.1 (5.4-12.3)	9.1 (5.9-13.6)
Echocardiographic data		
Bicuspid aortic valve	75 (26)	30 (18)
Stroke volume index, mL/m^2^	42 ± 7	43 ± 7
Peak aortic jet velocity, cm/s	274 ± 52	295 ± 56
Mean gradient, mm Hg	18 ± 8	21 ± 9
Aortic valve area, cm^2^	1.27 ± 0.31	1.18 ± 0.27
Indexed aortic valve area, cm^2^/m^2^	0.67 ± 0.16	0.63 ± 0.14
AS severity		
Mild AS	202 (67)	86 (51)
Moderate AS	36 (12)	32 (19)
Severe AS	7 (2)	7 (4)
Low-gradient severe AS	58 (19)	44 (26)
LV mass index, g/m^2^	105 ± 23	109 ± 24
E/e’ ratio	11.2 ± 3.8	11.9 ± 4.2
LV ejection fraction, %	64 ± 6	65 ± 6

Values are mean ± SD, n (%), or median (25th–75th percentiles).

ACE = angiotensin-converting enzyme; apoB = apolipoprotein B; ARB = angiotensin receptor blocker; AS = aortic valve stenosis; LDL-C = low-density lipoprotein cholesterol; LV = left ventricular; NT-proBNP = N-terminal pro B-type natriuretic peptide.

About follow-up, patients had between 1- and 10-year follow-up (between 1 and 11 visits spaced 1 year apart). But less than 25% of the patients have more than 5 years of follow-up (6 visits) (Supplemental Figure 1), therefore only the data for the first 6 visits were used for the training.

### Definition of study end point

We defined 2 main clinical end point events: 1) the occurrence of aortic valve intervention (SAVR or TAVI) or all-cause mortality; and 2) a change in AS hemodynamic severity (ie increase of AS grade from mild to moderate or mild/moderate to severe between baseline and last imaging follow-up).

To predict the occurrence of clinical end points in the next 5 years, all patient visits are labeled as class 1 if any of the end points are reached during any of their visits. Thus, the training was performed on a composite of aortic valve intervention, all-cause mortality, or AS hemodynamic progression. Considering that 5 years is a long-term event, we also trained a model to predict if a patient will have a clinical event in the next 2 years. Thus, if the patient matches at least one of the clinical end points in the next 2 years, the current visit was labeled as 1, otherwise 0.

### Generation of machine and deep learning models

Following deep learning terminology, each patient is a sample, and the visits are the time steps. Using Keras,^20^ we trained Gated Recurrent Unit (GRU) (Supplemental Figure 2) and Long Short-Term Memory (LSTM) models to predict the occurrence of a clinical end point in the next 5 years and next 2 years. Moreover, using scikit-Learn,^21^ we trained the following machine learning classifiers to predict the same end points: XGBoost, LightGBM, Naive Bayes, and Logistic regression. These models were trained on individual visits, treating each visit as an independent instance, using all features from the previously described data. This noncumulative approach means that each training instance consisted of data from a patient at a specific visit, without incorporating information from previous visits. We also conducted a cumulative analysis for the machine learning models. In this approach, features from each visit were added to the model incrementally, so that with each new visit, the number of features increased, reflecting the accumulation of data over time. All model’s hyperparameters are provided in Supplemental Table 1.

To train the clinical model to mirror current practice accuracy, we performed logistic regression using scikit-learn library with a specific subset of features. Based on previous findings,^22^ we selected clinically relevant features that are associated with faster progression of AS: age, sex, BMI, systolic blood pressure, hypertension, metabolic syndrome, diabetes, anticoagulant therapy, lipid-lowering agents, plasma level of low-density lipoprotein, triglycerides, apolipoprotein B, creatinine, serum calcium and phosphate, and AS hemodynamic severity (ie, V_peak_). We used logistic regression because it is a standard and widely used method for binary classification tasks, especially for clinical problems,^23^^,^^24^ allowing us to establish a baseline for comparison with more complex models.

For all trainings, the data set was split into 70%, 15%, 15% train, validation, and test sets, using random stratified sampling, where each set preserves original class balance. Deep learning training was stopped when the loss on the validation set stopped by early stopping on 150 consecutive epochs with a maximum of 1,000 epochs. We executed models 100 times with new random splits and reported the average performance on the test sets in the results.

### Statistical analysis

Continuous variables were presented as mean ± SD or median (IQR) for non-normally distributed variables. Continuous variables were compared between groups with Student’s *t*-test, or with Wilcoxon-Mann-Whitney test or Kruskal-Wallis test followed by Dunn’s post hoc test for non-normally distributed variables. Categorical variables were presented as frequencies and percentages and were compared with chi-square test or Fisher’s exact test as appropriate. We used receiver operating characteristic and area under curve (AUC) on the test sets to illustrate the diagnostic ability of the RNN model. A 2-tailed *P* value <0.05 was considered significant. Statistical analyses were performed with Stata software, version 14.2 (StataCorp). All metrics shown in the Results section were obtained with the Python package scikit-learn.^21^ Two-sided bootstrap CIs (95% CI) were computed using the stats.bootstrap function of the scipy Python package with default parameters.

## Results

### Study sample characteristics

Table 1 describes the baseline characteristics of the 303 patients included in this analysis. The mean age was 64 ± 14 years and 28% were women. Most of the patients had no or mild symptoms at baseline (99% of New York Heart Association class I or II). Comorbidities including hypertension, coronary artery disease, diabetes, and atrial fibrillation were present in 69%, 30%, 25%, and 13% of the study sample, respectively. Bicuspid aortic valve was present in 26% of patients. AS was hemodynamically mild (V_peak_ 2.0-2.9 m/s or gradient <20 mm Hg and AVA >1.5 cm^2^) in 67% of patients, moderate (V_peak_ 3.0-3.9 m/s, gradient 20-39 mm Hg or AVA 1.0-1.5 cm^2^) in 12%, severe (V_peak_ ≥4.0 m/s or gradient ≥40 mm Hg) in 2%, and 19% had severe low-gradient AS (V_peak_ <4.0 or gradient <40 mm Hg, and AVA≤1.0 cm^2^). None of the patients with severe AS had a class I or IIa indication for intervention at baseline.

### Predictive performance of machine and deep learning algorithms

During a mean follow-up of 4.4 ± 2.6 years (median: 4.0 years [IQR: 2.3-6.0]), a total of 198 (65%) patients had worsening of AS hemodynamic severity grade, 104 (34%) underwent AVR, and 24 (8%) died. The composite end point (ie any of the clinical events) occurred in a total of 260 (86%) patients. LightGBM and XGBoost, followed by GRU and LSTM demonstrated high predictive performance to predict the risk of clinical events on the test sets with better accuracy than the logistic regression model. Test sets were composed of 46, 46, 38, 29, 20, and 17 patients for years 1 to 5, respectively. Out of the 100 training runs, the optimal epoch, on average, occurred at approximately epoch 187.

**Figure 1:**
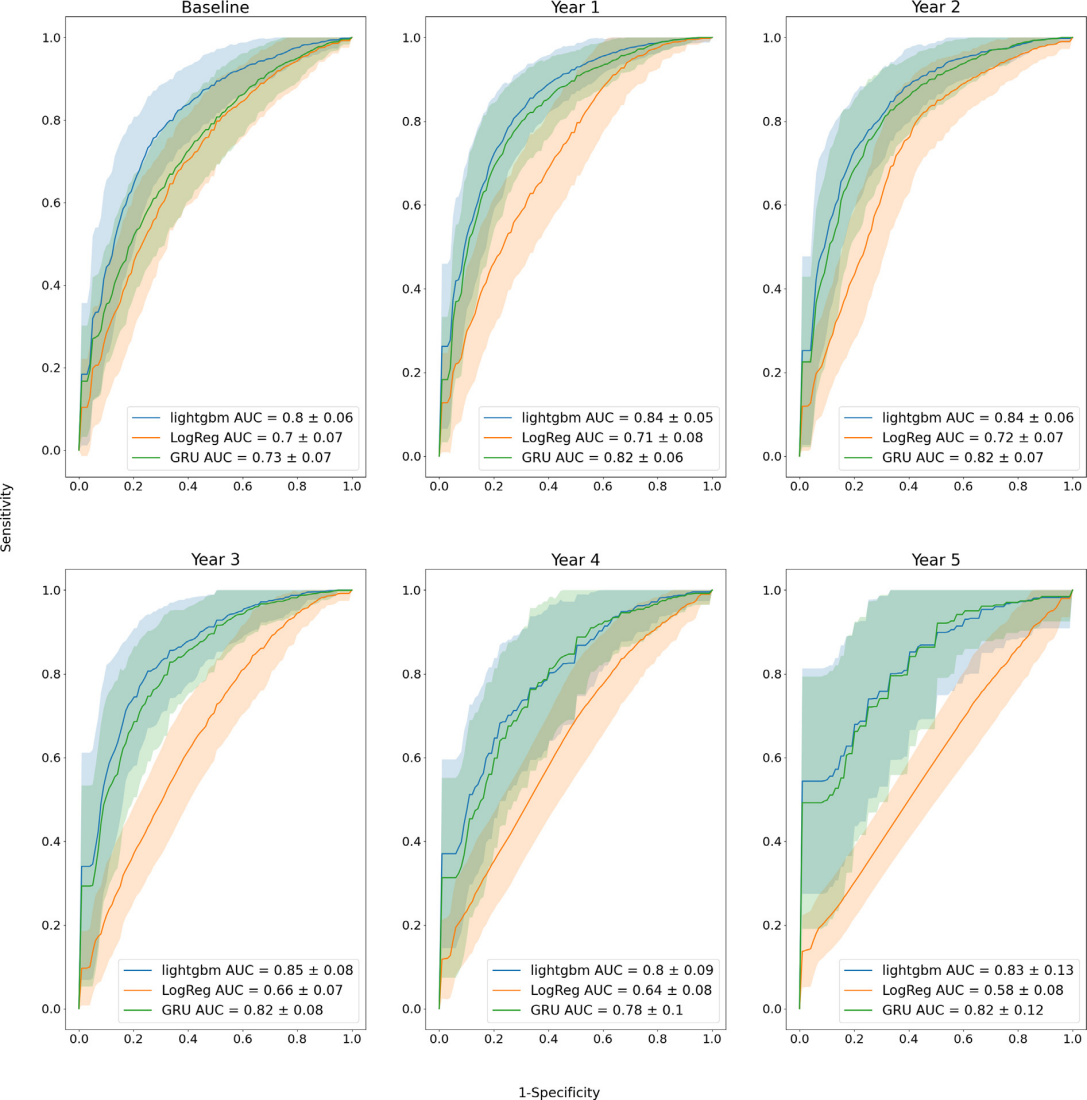
**Performance of the Trained Models Over 5 Years to Predict AS Progression in a 5-Year Term** ROC curve analysis of the lightGBM and GRU models for each visit predicts the final clinical end point within 5 years using all features, compared with logistic regression using the 22 clinical features (ie the clinical model); the orange and blue areas illustrate ROC curves from 100 test sets for all both models. Only 3 models are shown here, other ROC curves for all models are presented in Supplemental Figure 4. AS = aortic valve stenosis; AUC = area under curve; GRU = Gated Recurrent Unit; ROC = receiver operating characteristic.

**Figure 2:**
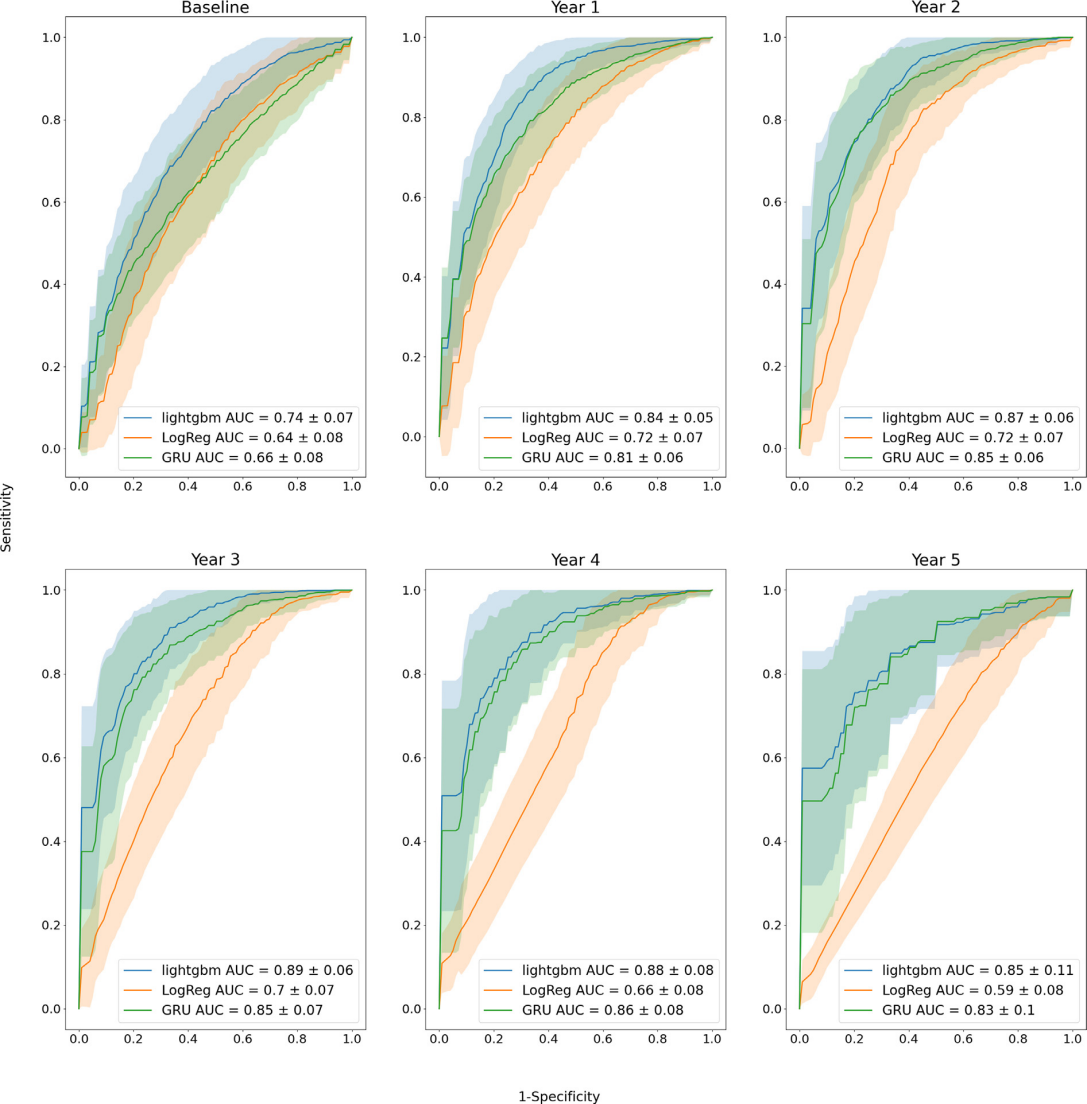
**Performance of the Trained Models Over 5 Years to Predict AS Progression in a 2**-**Year Term** ROC curve analysis of the lightGBM and GRU models for each visit predicts the final clinical end point within 2 years using all features, compared with logistic regression using the 22 clinical features (ie, the clinical model); the orange and blue areas illustrate ROC curves from 100 test sets for all both models. Only 3 models are shown here, other ROC curves for all models are presented in Supplemental Figure 5. Abbreviations as in Figure 1.

The best performance results were obtained with the noncumulative approach, so when all visits have been used for training. For each annual follow-up visit, the LightGBM model consistently provided higher AUCs compared to the clinical model, with an average of 0.83 (95% CI: 0.81-0.84) and 0.67 (95% CI: 0.66-0.69), respectively, across visits (Figure 1, Tables 2 and 3). LightGBM average performance was closely followed by XGBoost (0.82 [95% CI: 0.80-0.83]), GRU (0.80 [95% CI: 0.79-0.81]), LSTM (0.79 [95% CI: 0.78-0.81]), Logistic regression (0.78 [95% CI: 0.77-0.80]), and Naive Bayes (0.76 [95% CI: 0.74-0.77]). The calibration data of the GRU model are presented in Supplemental Figure 3. We obtained equivalent trends when predicting the risk of future clinical events over a 2-year term, which necessitated a class modification based on clinical end point date. As shown in Figure 2 and Table 4, LightGBM model consistently provided higher AUCs compared to the clinical model, with an average of 0.85 (95% CI: 0.83-0.86) and 0.67 (95% CI: 0.66-0.69), respectively, across visits. LightGBM performance was closely followed by XGBoost (0.84 [95% CI: 0.83-0.85]), GRU (0.81 [95% CI: 0.79-0.82]), LSTM (0.81 [95% CI: 0.79-0.82]), Logistic regression (0.78 [95% CI: 0.76-0.80]), and Naive Bayes (0.72 [95% CI: 0.70-0.74]).

**Table 2:** XGBoost, GRU, Logistic Regression, LightGBM, LSTM, Naïve Bayes Models, and the Clinical Model in Predicting Clinical Outcomes at Different Visit Intervals Over a 5-Year Term Using the Noncumulative Approach

Visit	Prediction at 5-Year Term						
	XGboost	GRU	Logistic Regression	LightGBM	LSTM	Naïve Bayes	Clinical Model
Baseline	**0.80 (0.79-0.81)**	0.73 (0.72-0.74)	0.73 (0.72-0.75)	**0.80 (0.79-0.81)**	0.72 (0.71-0.74)	0.72 (0.70-0.73)	0.70 (0.69-0.72)
1 year	0.83 (0.82-0.84)	0.82 (0.81-0.83)	0.80 (0.79-0.81)	**0.84 (0.83-0.85)**	0.81 (0.80-0.82)	0.79 (0.78-0.80)	0.71 (0.70-0.73)
2 years	0.83 (0.82-0.84)	0.82 (0.81-0.83)	0.80 (0.79-0.82)	**0.84 (0.83-0.85)**	0.81 (0.80-0.82)	0.77 (0.76-0.79)	0.72 (0.71-0.74)
3 years	0.84 (0.83-0.86)	0.82 (0.81-0.84)	0.79 (0.78-0.81)	**0.85 (0.83-0.86)**	0.81 (0.80-0.83)	0.77 (0.75-0.78)	0.66 (0.64-0.67)
4 years	0.79 (0.77-0.81)	0.78 (0.76-0.80)	0.75 (0.73-0.78)	**0.80 (0.78-0.81)**	0.78 (0.76-0.80)	0.76 (0.74-0.77)	0.64 (0.62-0.65)
5 years	0.81 (0.78-0.83)	0.82 (0.80-0.84)	0.81 (0.79-0.84)	**0.83 (0.80-0.85)**	0.82 (0.79-0.84)	0.72 (0.69-0.75)	0.58 (0.57-0.60)
Average	0.82 (0.80-0.83)	0.80 (0.79-0.81)	0.78 (0.77-0.80)	**0.83 (0.81-0.84)**	0.79 (0.78-0.81)	0.76 (0.74-0.77)	0.67 (0.66-0.69)

Values are AUC (95% CI). Bold represent the best score of a row. If scores matches, the one with the higher 95% CI is bold. If score and 95% CI match between 2 scores, then both are bold.

AUC = area under curve; GRU = Gated Recurrent Unit; LSTM = Long Short-Term Memory.

**Table 3:** XGBoost, GRU, Logistic Regression, LightGBM, LSTM, Naïve Bayes Models, and the Clinical Model in Predicting Clinical Outcomes at Different Visit Intervals Over a 2-Year Term Using the Noncumulative Approach

Visit	Prediction at 2-Year Term						
	XGboost	GRU	Logistic Regression	LightGBM	LSTM	Naïve Bayes	Clinical Model
Baseline	0.73 (0.72-0.74)	0.66 (0.64-0.67)	0.67 (0.65-0.68)	**0.74 (0.72-0.75)**	0.65 (0.64-0.67)	0.61 (0.60-0.63)	0.64 (0.62-0.65)
1 year	0.83 (0.82-0.84)	0.81 (0.79-0.82)	0.79 (0.78-0.80)	**0.84 (0.83-0.85)**	0.80 (0.79-0.81)	0.75 (0.74-0.77)	0.72 (0.71-0.74)
2 years	0.86 (0.85-0.87)	0.85 (0.83-0.86)	0.82 (0.81-0.83)	**0.87 (0.85-0.88)**	0.84 (0.83-0.85)	0.76 (0.74-0.77)	0.72 (0.71-0.73)
3 years	0.88 (0.87-0.90)	0.85 (0.84-0.87)	0.84 (0.82-0.85)	**0.89 (0.88-0.90)**	0.85 (0.84-0.87)	0.73 (0.71-0.76)	0.70 (0.68-0.71)
4 years	0.88 (0.86-0.89)	0.86 (0.84-0.87)	0.79 (0.77-0.81)	**0.88 (0.87-0.90)**	0.86 (0.84-0.88)	0.73 (0.71-0.75)	0.66 (0.65-0.68)
5 years	0.85 (0.83-0.87)	0.83 (0.81-0.85)	0.77 (0.74-0.80)	**0.85 (0.83-0.87)**	0.83 (0.81-0.85)	0.73 (0.71-0.76)	0.59 (0.58-0.61)
Average	0.84 (0.83-0.85)	0.81 (0.79-0.82)	0.78 (0.76-0.80)	**0.85 (0.83-0.86)**	0.81 (0.79-0.82)	0.72 (0.70-0.74)	0.67 (0.66-0.69)

Values are AUC (95% CI). Bold represent the best score of a row. If scores matches, the one with the higher 95% CI is bold. If score and 95% CI match between 2 scores, then both are bold.

Abbreviations as in Table 2.

**Table 4:** Performance of the LightGBM Model Over Time According to the Median Age of the Sample Population to Predict AS Progression Over the 5-Year Term

Visit	Prediction at 5-Year Term With LightGBM		Prediction at 2-Year Term With LightGBM	
	≤68	>68	≤68	>68
Baseline	**0.82 (0.81-0.83)**	0.77 (0.75-0.79)	**0.75 (0.73-0.77)**	0.73 (0.71-0.75)
1 year	**0.84 (0.83-0.86)**	0.84 (0.82-0.85)	0.84 (0.82-0.86)	**0.85 (0.83-0.86)**
2 years	**0.84 (0.82-0.86)**	**0.84 (0.82-0.86)**	0.86 (0.84-0.87)	**0.87 (0.85-0.89)**
3 years	0.83 (0.81-0.85)	**0.86 (0.84-0.88)**	0.88 (0.86-0.90)	**0.90 (0.88-0.92)**
4 years	**0.83 (0.80-0.86)**	0.76 (0.72-0.79)	**0.92 (0.90-0.94)**	0.83 (0.81-0.86)
5 years	**0.87 (0.83-0.90)**	0.78 (0.73-0.83)	0.84 (0.81-0.87)	**0.85 (0.81-0.88)**
Average	**0.84 (0.82-0.86)**	0.81 (0.78-0.83)	**0.85 (0.83-0.87)**	0.84 (0.82-0.86)

Values are AUC (95% CI). Bold represent the best score of a row. If scores matches, the one with the higher 95% CI is bold. If score and 95% CI match between 2 scores, then both are bold. The patients of the test set were divided according to the median age of the cohort (68 years) and then the performance of the model was analyzed.

Abbreviation as in Table 1.

In a cumulative approach (Supplemental Figures 5 and 6, Table 3), LightGBM and XGBoost performance remained consistently high. GRU and LSTM, which use historical data (past visits), maintained robust performance but did not significantly outperform LightGBM and XGBoost models. Finally, the clinical model had the lowest AUC scores.

We further stratified the analysis based on age and sex. When compared according to the median age of the whole cohort (ie 68 years), the average AUC performance over all visits of the models in predicting the risk of events on a 5-year term was slightly higher for younger (≤68 years) patients (0.84 [95% CI: 0.82-0.86]) compared to older patients (0.81 [95% CI: 0.78-0.83]), but not different on a 2-year term (Table 4). Between women and men, the model provided a better performance to predict women at 5- and 2-year terms at almost every visit (Table 5).

**Table 5:** Performance of the LightGBM Model Over Time According to Sex of the Sample Population to Predict AS Progression Over the 5-Year Term

Visit	Prediction at 5-Year Term With LightGBM		Prediction at 2-Year Term With LightGBM	
	Women	Men	Women	Men
Baseline	**0.84 (0.82-0.87)**	0.79 (0.77-0.80)	0.73 (0.70-0.77)	**0.74 (0.72-0.76)**
1 year	**0.87 (0.85-0.89)**	0.83 (0.82-0.84)	0.83 (0.81-0.85)	**0.85 (0.84-0.86)**
2 years	**0.85 (0.83-0.87)**	0.84 (0.82-0.85)	**0.91 (0.88-0.93)**	0.85 (0.84-0.86)
3 years	**0.86 (0.83-0.90)**	0.84 (0.82-0.86)	**0.89 (0.87-0.92)**	0.89 (0.88-0.90)
4 years	**0.84 (0.80-0.88)**	0.78 (0.76-0.80)	**0.90 (0.86-0.94)**	0.88 (0.86-0.90)
5 years	**0.97 (0.94-1.00)**	0.75 (0.72-0.79)	**0.93 (0.89-0.97)**	0.80 (0.77-0.84)
Average	**0.87 (0.85-0.90)**	0.81 (0.79-0.82)	**0.87 (0.84-0.90)**	0.84 (0.82-0.85)

Values are AUC (95% CI). Bold represent the best score of a row. If scores matches, the one with the higher 95% CI is bold. If score and 95%CI match between 2 scores, then both are bold. The patients of the test set were split according to their sex and then the performance of the model was analyzed in the 2 groups.

Abbreviation as in Table 1.

Finally, a substantial proportion (26%) of the patients included in this cohort had a bicuspid aortic valve. We observed a slight significant performance difference between models trained with tricuspid or with bicuspid samples at a 2-year term (0.78 [95% CI: 0.74-0.83] and 0.86 [95% CI: 0.84-0.88], respectively), but not at 5 years (Supplemental Tables 3A to 3Z).

Finally, while we determined the best models based on AUCs performance, we generally obtained similar observations in terms of Matthew’s Correlation Coefficient (also called Phi Coefficient) performance (Supplemental Tables 4A to 4Z) and sensitivity (Supplemental Tables 5A to 5Z).

### Features importance

We also evaluated the importance of each feature using SHapley Additive exPlanations (SHAP)^25^ used by the 5-year term LightGBM model during the prediction phase (Table 6). This analysis was performed on validation data from each clinical visit. We chose the features based on SHAP values that are important in the prediction, ie greater than 0.1 explainability, for a total of 29 features related to severity of aortic stenosis, hemodynamic stress, left ventricular structure and function, general health status, age, and metabolic milieu.

**Table 6:** Average Importance of Each Feature Across Visits Using SHapley Additive exPlanations (SHAP) Values in the 5-Year Term LightGBM Model

Feature Name	Average SHAP Value
Peak aortic jet velocity	0.070
Aorta time velocity integral	0.058
Transvalvular mean gradient	0.036
Change in aortic peak velocity over tertiles	0.036
Aortic valve area	0.030
Aorta ascending	0.029
Platelets	0.026
Age	0.024
AVA index	0.023
Left ventricular outflow tract diameter	0.022
Cholesterol total	0.020
Moderate AS progression echo	0.018
Serum creatinine	0.017
C-reactive protein	0.016
Echo heart rate	0.015
Left ventricular posterior wall diastole	0.015
Left ventricular mass indexed to body surface area	0.014
Aortic root diameter	0.013
Fasting insulin level	0.013
Interventricular septum diastole	0.013
Body mass index	0.013
White blood cells	0.012
Left ventricular mass	0.012
Red globules	0.012
Collagen-adenosine interaction time	0.011
Serum phosphate	0.011
Mitral valve A wave	0.010
Left ventricular dimension in diastole	0.010
Mean glomerular volume	0.010

Only features with a 0.1 score explainability were retained. The Table with all visits is presented in Supplemental Table 2.

AVA = aortic valve area; other abbreviation as in Table 1.

## Discussion

The present study is, to our knowledge, the first to develop and validate machine and deep learning-based algorithms with multisource data including clinical, laboratory, and imaging features from a prospective longitudinal cohort study of patients with AS. We demonstrated that the tree-based models and RNNs are highly effective in predicting the future risk of rapid disease progression and adverse clinical events in AS (Central Illustration). This supports the clinical utility of an AI-guided approach to improve prediction of disease progression, risk stratification, and therapeutic decision-making in AS.

**Central Illustration:**
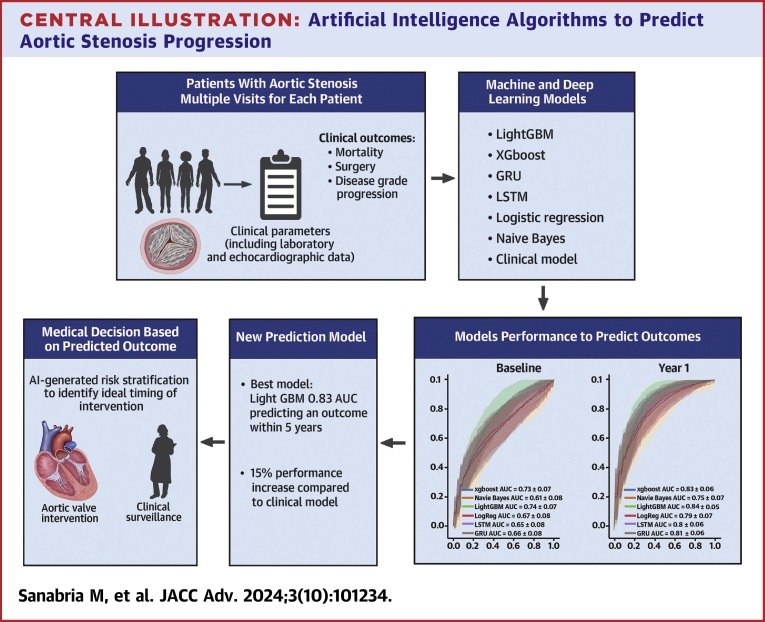
**Artificial Intelligence Algorithms to Predict Aortic Stenosis Progression** Schematic representation of the study, machine and deep learning algorithms chosen and overall performance to predict the risk of aortic valve stenosis progression and related outcome. AI = artificial intelligence; LSTM = Long Short-Term Memory; other abbreviations as in Figure 1.

### AI approach for risk stratification in AS

Previous studies have evaluated the ability of AI to improve detection of AS severity by various diagnostic modalities but also for risk stratification, with better performances than conventional clinical risk scores.^7^, ^8^, ^9^^,^^26^ However, predicting the progression of AS remains an unmet need. Previous studies reported that the AS progression is extremely variable from one patient to the other and difficult to predict.^27^, ^28^, ^29^ The most important predictor of AS progression reported in these previous studies is the baseline hemodynamic/anatomic severity. However, such factors explain only a fraction of the variance in AS progression. Our analysis shows that incorporating a broader array of data sources significantly enhances predictive performance, with the logistic regression model using extensive features substantially outperforming the clinical model with fewer features. Additionally, the integration of historical data generally yielded comparable or slightly reduced performance across most tested models, except for the clinical model, which showed improved accuracy with cumulative data. These observations emphasize the uncertainty in AS progression prediction and suggest potential integration of machine learning algorithms with clinical and echocardiographic assessments for a better monitoring of disease progression.

### Importance of features for risk prediction

Compared to previous studies, we used clinical, metabolic, and echocardiographic data as input for our models, as AS is a complex and multifaceted disease with several risk and/or associated factors for the development and progression of the disease.^22^^,^^30^^,^^31^ Indeed, older age, degree of valve stenosis and/or calcification, lipid-related factors, metabolic syndrome, vitamin K antagonists, renal disease, osteoporosis, or calcium-phosphate dysmetabolism are among the factors identified as predictors for faster progression of AS.^15^^,^^22^^,^^27^, ^28^, ^29^^,^^32^, ^33^, ^34^, ^35^, ^36^, ^37^

In the present study, top features (Table 6) were echocardiographic parameters either related to the degree or change in AS severity, anatomical and functional cardiac damage, including: V_peak_ and AVA, LV posterior and interventricular septal wall thickness, ascending aorta diameter, or A wave velocity of the mitral annulus measured by Doppler tissue imaging. These findings further place Doppler echocardiography as a first-line imaging modality for disease monitoring and risk stratification. Other features reveal the presence of hemodynamic stress in the left ventricular structure and function, including left ventricular mass and outflow tract diameter, interventricular septum diastole, left ventricular posterior wall diastole, and left ventricular dimension in diastole are related to left ventricular structure and function. These findings correlate with previous studies.^38^^,^^39^ Few features related to metabolic milieu have been also identified, including BMI, plasma level of total cholesterol, serum creatinine, C-reactive protein, fasting insulin level, white blood cells, red globules and serum phosphate, which is consistent with prior results.^14^^,^^40^, ^41^, ^42^ These findings provide further support to the contribution of lipid-related biomarkers to the development and/or progression of AS. Other “traditional” metabolic factors previously linked to faster progression rate of AS were not among these top features, suggesting that they may not be major drivers of disease progression and associated outcome. However, further research is needed to determine whether the accuracy of the developed models can be enhanced by incorporating additional imaging biomarkers such as aortic valve calcification measured by computed tomography, along with emerging blood biomarkers.

### Model performance according to sample population

Cohen-Shelly et al^9^ reported better performance of their AI-enabled electrocardiogram for AS screening in older patients as well as women, supported by other findings that confirm that the clinical presentation, pathophysiological responses, and clinical outcomes of AS are different in women versus men, and in older versus younger patients.^30^^,^^43^^,^^44^ In our study, regarding age-related performance, younger patients were slightly easier to predict, though the CIs overlapped, indicating a nonsignificant difference. However, we found that models predicting AS progression provide much better performances in women compared to men at 5 years. Several potential reasons could explain these differences. The features included in the model might capture factors that are more predictive of AS progression in women. Additionally, AS may have different patterns or risk factors in women, which the model may detect more effectively. Variations in clinical presentation, disease progression, or the quality and type of data available for men versus women may also contribute. These insights emphasize the need for demographic-specific adjustments in predictive models to enhance the accuracy and reliability of AS progression predictions and provide personalized and effective clinical interventions.

### Choosing the right learning approach: deep versus machine algorithm

Our study highlights the efficacy of advanced machine learning algorithms, particularly models like LightGBM and XGBoost, in noncumulative or cumulative approaches, demonstrating their strength in handling accumulated data effectively to predict clinical outcomes more accurately than traditional methods. RNN models, which use historical data, maintain robust performance but do not outperform the top noncumulative models. Furthermore, deep learning algorithms generally require large amounts of data, because of their complex architectures and numerous parameters, to effectively learn and generalize patterns. This contrasts with classical machine learning models, which often perform well even with smaller data sets due to their simpler structures and fewer parameters. These approaches hold promise to unravel complex pathophysiological mechanisms implicated in disease progression.

### Study limitations

The cohort used for this analysis was predominantly composed of patients with mild or moderate AS. However, the most important unmet need in terms of risk stratification and individualization of the timing of follow-up and management is more in this subset with mild/moderate AS rather than in severe AS. It is now well known that most patients with severe AS had adverse outcomes in the short term and should be considered for early AVR. Accordingly, the guidelines now include several class IIa indications for early AVR in asymptomatic patients with severe AS.^3^ In the present study, we thus only included the patients with severe AS who had no indication (I or IIa) for AVR at baseline. Early AVA is not yet recommended in patients with moderate AS. However, several trials (TAVR-UNLOAD [Transcatheter Aortic Valve Replacement to UNload the Left Ventricle in Patients With ADvanced Heart Failure], PROGRESS [Management of Moderate Aortic Stenosis by Clinical Surveillance or TAVR], and EXPAND TAVR II Pivotal Trial [NCT05149755]) are currently assessing the timing of AVR in patients with moderate AS. The results of these trials have the potential to change and improve the clinical management of these patients. These trials provide support to the relevance and utility of the AI-based predictive model that we proposed and validated in the present study.

While our study shows promising results, the relatively small sample size may limit the generalizability of our findings, especially considering a new cohort may vary across different clinical settings and samples. In the future, we plan to expand our cohort by including more patients, which will help to confirm the validity of the proposed models.

## Conclusions

This study shows that machine and deep learning algorithms based on multisource comprehensive data provide high accuracy to predict disease progression and clinical outcomes in patients with mild-to-moderate AS at baseline. These findings further support the implementation of the AI-guided approach in clinical routine to enhance risk stratification and help for identifying the best timing for intervention in patients with AS.

Perspectives **COMPETENCY IN MEDICAL KNOWLEDGE:** In patients with mild-to-moderate AS at baseline, machine and deep learning algorithms applied to multisource clinical, laboratory, and imaging data outperform traditional clinical models to predict future AS progression.

**TRANSLATIONAL OUTLOOK:** Future research should validate these models across diverse AS populations and integrate them into clinical workflows. This AI-guided approach could contribute to optimize patient risk stratification and monitoring, paving the way for tailored and personalized clinical management of mild-to-moderate AS.

## Funding support and author disclosures

This work has been supported by MITACS Globalink (IT25650), 10.13039/501100000024Canadian Institutes of Health Research (#FDN-143225 and MOP-114997), Foundation of the Québec Heart and Lung Institute, Fonds de Recherche du Québec en Santé (FRQS), France Health Data Hub (HDH), and institutional research funds held by Drs Droit and Precioso. Dr Pibarot has received funding from 10.13039/100006520Edwards Lifesciences and 10.13039/100004374Medtronic for echocardiography core laboratory analyses in the field of transcatheter and surgical aortic valve replacement with no direct personal compensation. All other authors have reported that they have no relationships relevant to the contents of this paper to disclose.
