# Supplementary material for: AI-Enhanced Prediction of Aortic Stenosis Progression: Insights From the PROGRESSA Study
Source: JACC Adv. 2024 Sep 11;3(10):101234. doi: 10.1016/j.jacadv.2024.101234 (PMC11416525; doi:10.1016/j.jacadv.2024.101234)
Supplement: Supplemental_Material [file mmc1.docx]

[
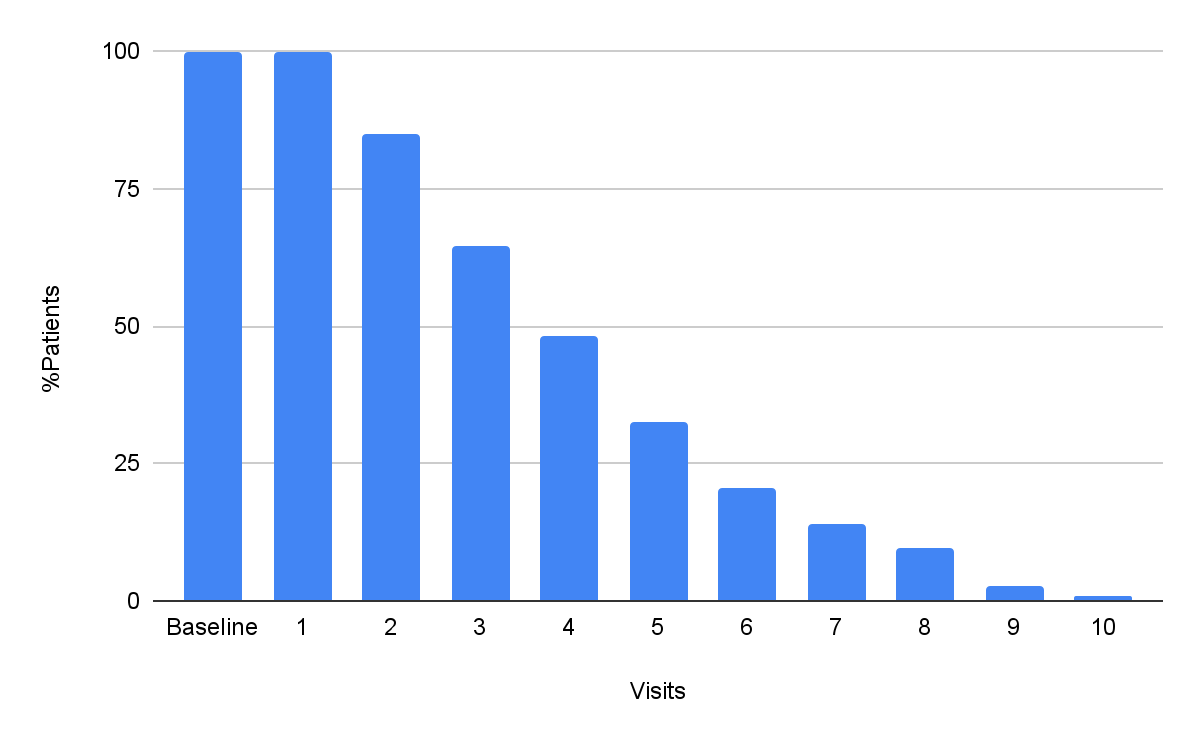
](https://docs.google.com/document/d/112U5u1xdMjqIeKcfCxY3ircntahwepuV5ksXx5H0oAo/edit#D2L_fig_label_Percentage%20of%20patients%20having%20at%20least%20n%20number%20of%20visits)

**Figure S1**: Percentage of patients having at least *n* number of visits. Depending on the progress of the disease, the patients in the dataset have total visits from 1 to 11 visits, with a Baseline visit and up to 10 years of follow-up. Less than 25% of the patients have more than 5 years of follow-up (or 6 visits), therefore only the data for the first 6 visits were used for the generation of our RNN model. Furthermore, the cohort included 13 cardiac-related deaths, 18 non-cardiac-related deaths, and 31 cases with an unknown cause of death.

**Table S1**: Hyperparameter list chosen for each model

| **XGboost** | **lightGBM** | **Naive Bayes** | **Logistic Regression** | **GRU & LSTM** |
| --- | --- | --- | --- | --- |
| booster=gbtree  eta=0.3  gamma=0  max_depth=6  min_child_weight=1  max_delta_step=0  subsample=1  sampling_method=uniform  colsample_bytree=1  colsample_bylevel=1  colsample_bynode=1  lambda=1  alpha=0  tree_method=auto  scale_pos_weight=1  refresh_leaf=1  process_type=default  grow_policy=depthwise  max_leaves=0  max_bin=256  multi_trategy=one_output_per_tree | boosting_type=gbdt  num_leaves=31  max_depth=-1  learning_rate=0.1  n_estimators=100  subsample_for_bin=200000  objective=None  class_weight=None  min_split_gain=0.  min_child_weight=1e-3  min_child_samples=20  subsample=1.  subsample_freq=0  colsample_bytree=1.  reg_alpha=0.  reg_lambda=0.  random_state=None  n_jobs=None  importance_type='split' | priors=None  var_smoothing=1e-9 | penalty=l2  dual=False  tol=1e-4  C=1.0  fit_intercept=True  intercept_scaling=1  class_weight=None  solver=lbfgs  max_iter=100 | units=16  dropout=0.5  recurrent_dropout=0.5  GRU activation=tanh  use_bias=True  kernel_initializer=glorot_uniform  recurrent_initializer=zeros  dense layer activation=sigmoid  loss=binary_crossentropy |


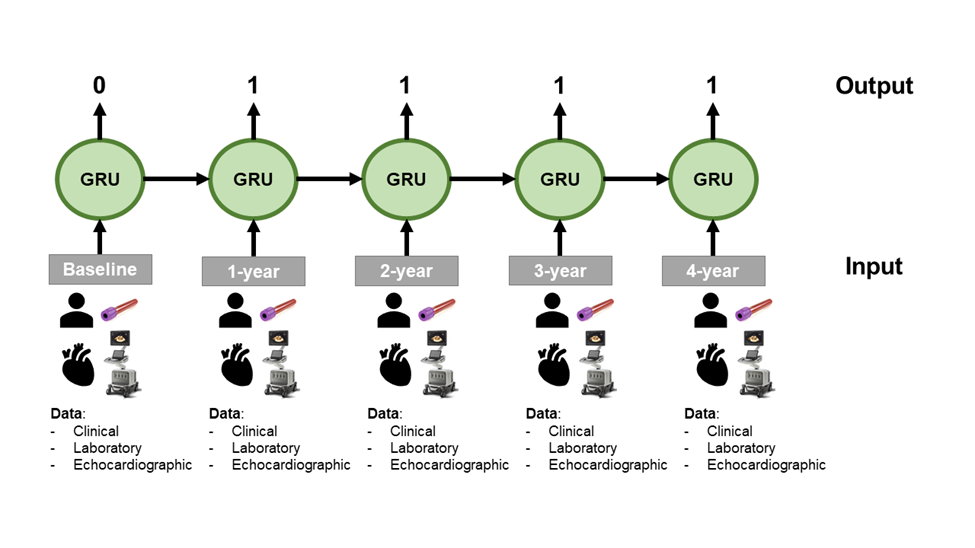


**Figure S2: Schematic Representation of the Recurrent Neural Network model**.

In the model, there are feature vectors as input for each visit and their respective prediction. Input contains clinical laboratory and echocardiographic data. GRU stands for Gated Recurrent Unit, a type of Recurrent Neural Network.


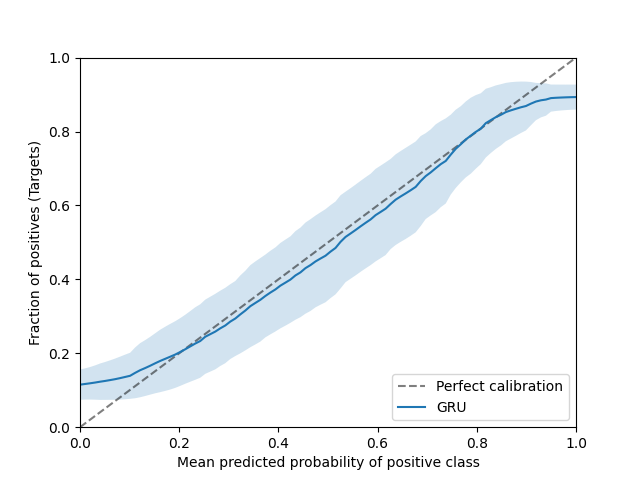


**Figure S3:** Calibration plot of the 100 models that were trained on a 5-year term with GRU.

**
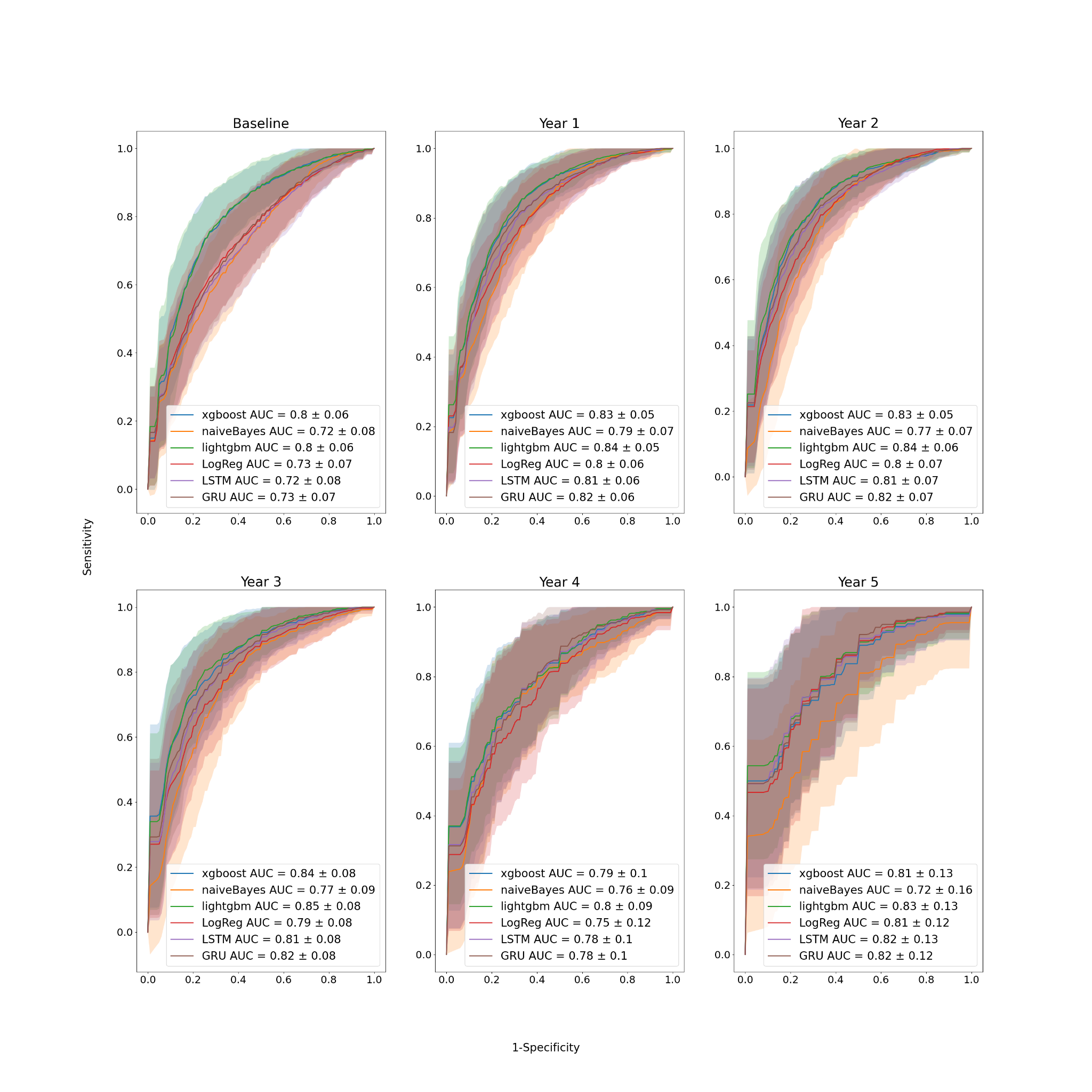
**

**Figure S4:** **Performance of the trained models over 5 years to predict AS progression in a 5 years term.** ROC curve analysis of the XGboost, Naive Bayes, lightGBM, logistic regression, LSTM and GRU models for each visit predicts the final clinical endpoint within five years using all features. The areas illustrate ROC curves from 100 test sets for all models with their respective colors.

**
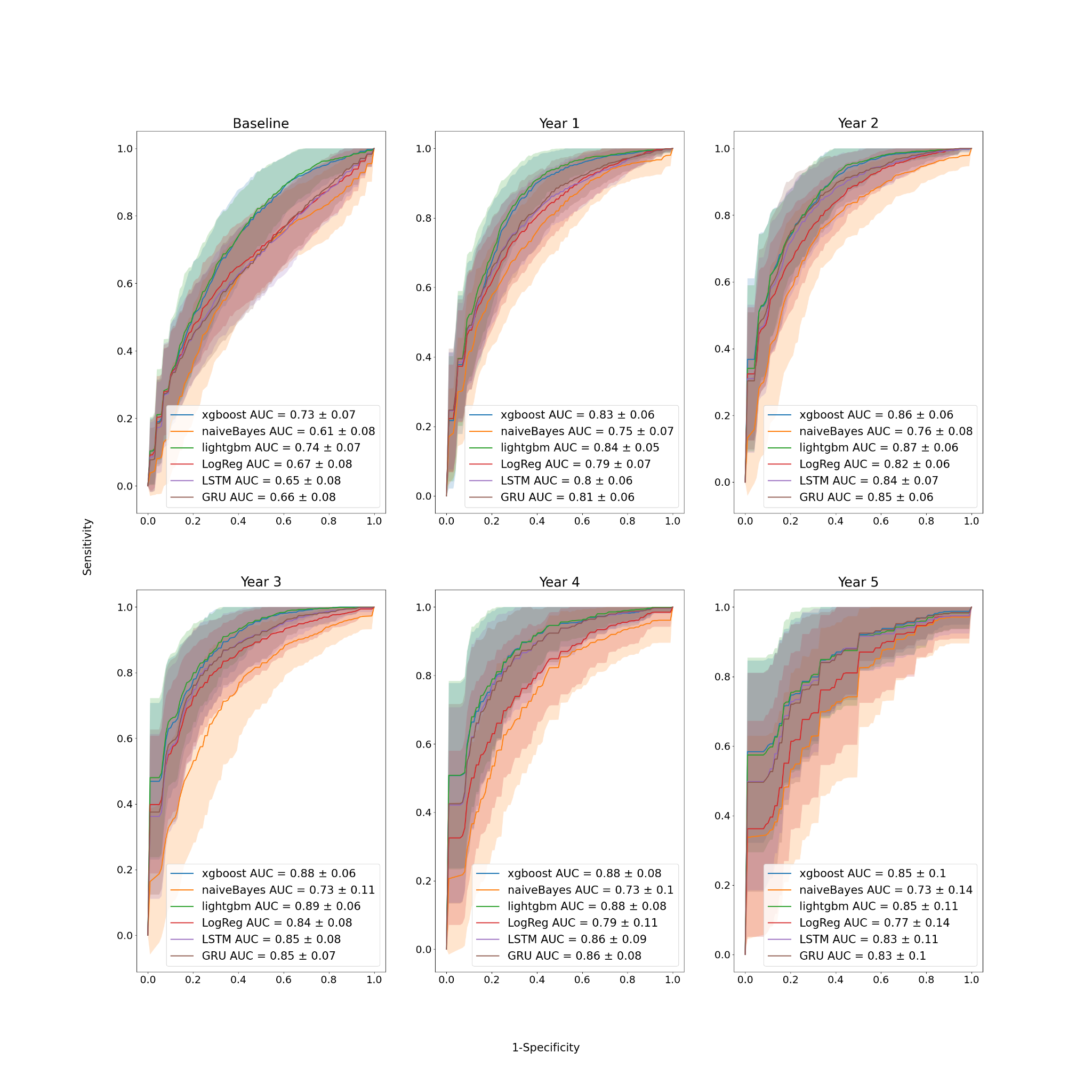
**

**Figure S4:** **Performance of the trained models over 5 years to predict AS progression in a 2 years term.** ROC curve analysis of the XGboost, Naive Bayes, lightGBM, logistic regression, LSTM and GRU models for each visit predicts the final clinical endpoint within five years using all features. The areas illustrate ROC curves from 100 test sets for all models with their respective colors.

**
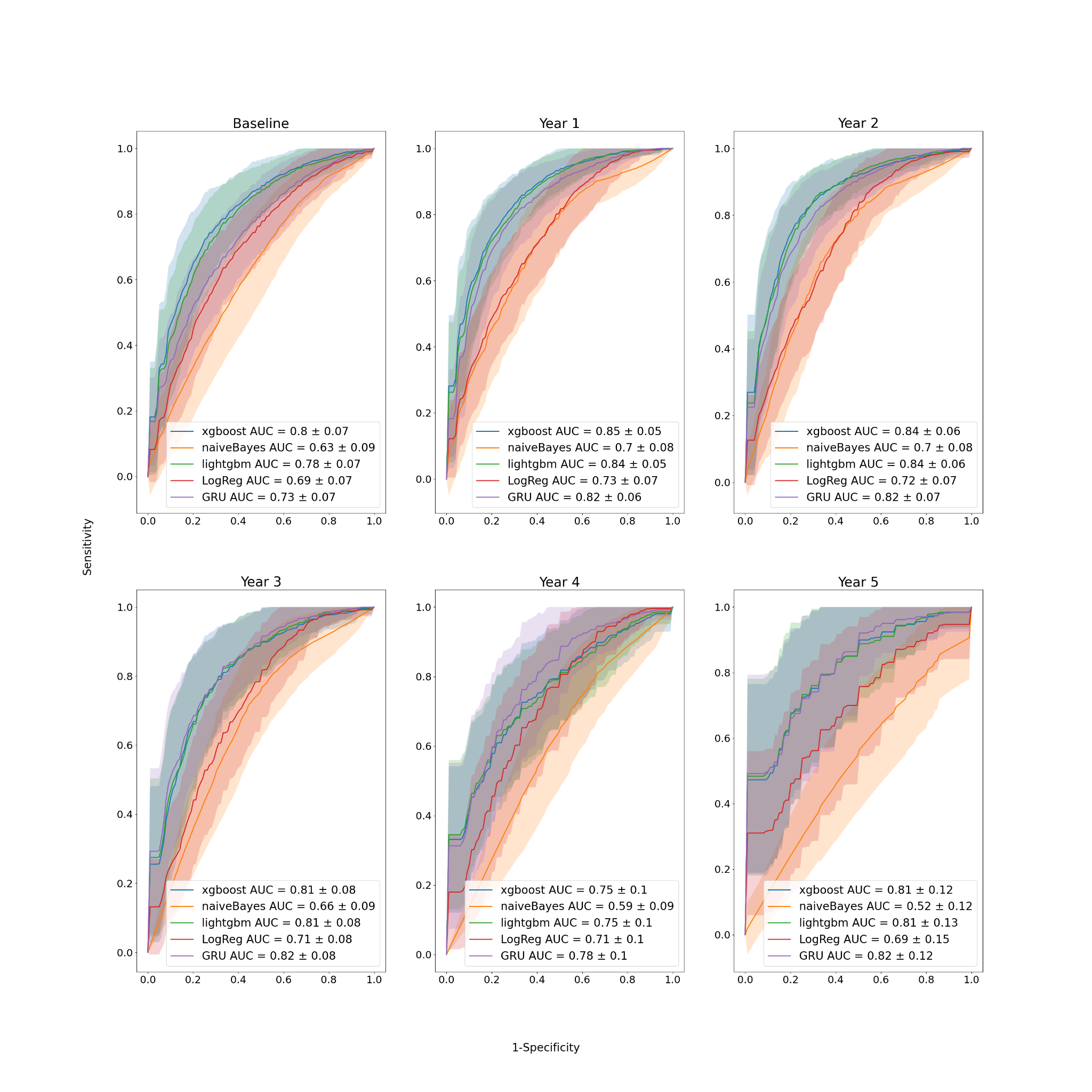
**

**Figure S5:** **Performance of the trained models over 5 years to predict AS progression in a 5 years term using cumulative approach.** ROC curve analysis of the XGboost, Naive Bayes, lightGBM, logistic regression, LSTM and GRU models for each visit in a cumulative approach predicts the final clinical endpoint within five years using all features. The areas illustrate ROC curves from 100 test sets for all models with their respective colors.

**
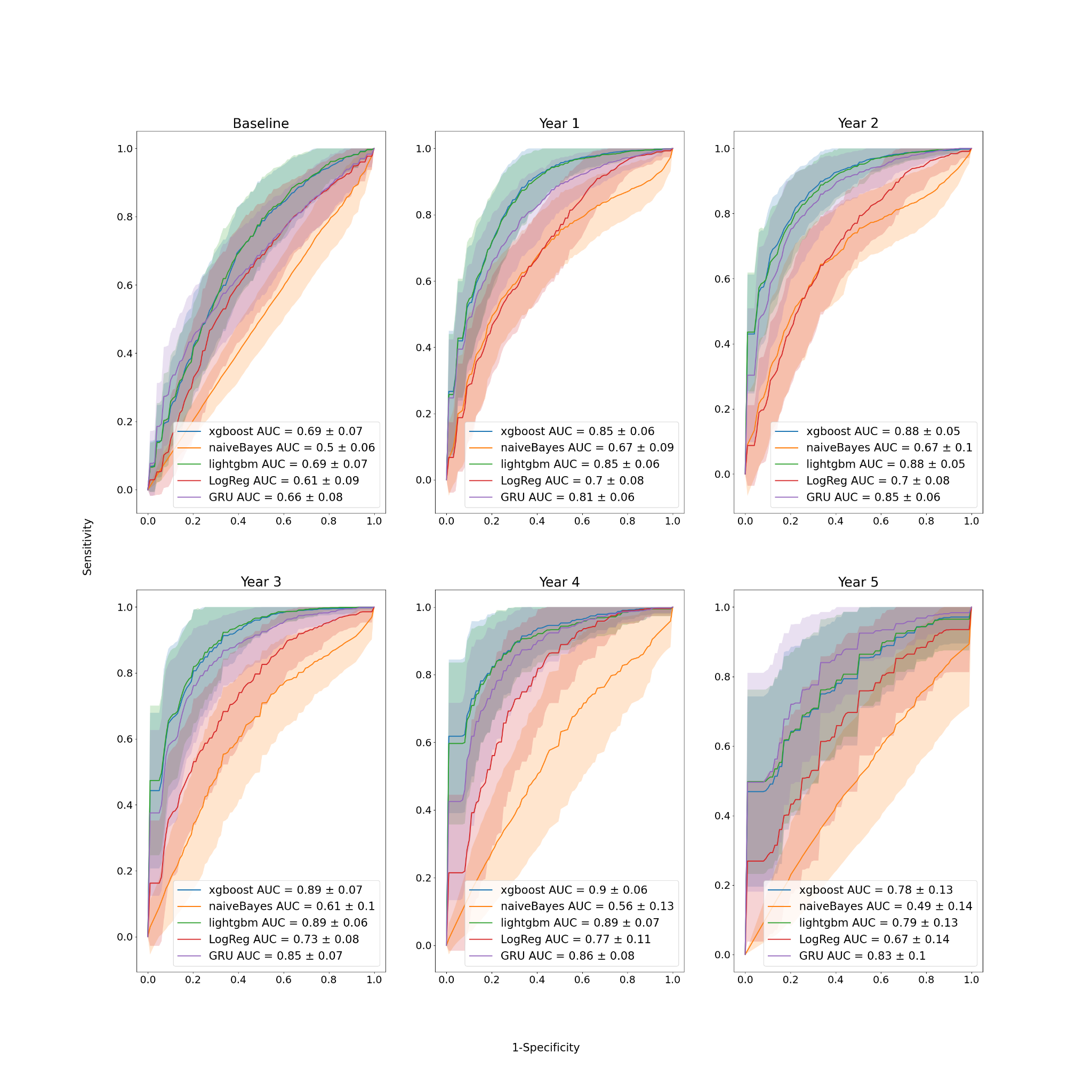
**

**Figure S6:** **Performance of the trained models over 5 years to predict AS progression in a 2 years term using cumulative approach.** ROC curve analysis of the XGboost, Naive Bayes, lightGBM, logistic regression, LSTM and GRU models for each visit in a cumulative approach predicts the final clinical endpoint within five years using all features. The areas illustrate ROC curves from 100 test sets for all models with their respective colors.

**Table S2**: Importance of each feature across visits using SHapley Additive exPlanations (SHAP) values in the 5-years term lightGBM model. Only features with a 0.1 score explainability were retained. The table is ordered using the average of SHAP values over all visits.

|  | **Baseline** | **Visit 1** | **Visit 2** | **Visit 3** | **Visit 4** | **Visit 5** | **Average** |
| --- | --- | --- | --- | --- | --- | --- | --- |
| **Peak aortic jet velocity** | 0.075 | 0.070 | 0.070 | 0.068 | 0.069 | 0.069 | 0.070 |
| **Aorta time velocity integral** | 0.057 | 0.060 | 0.056 | 0.061 | 0.059 | 0.054 | 0.058 |
| **Transvalvular mean gradient** | 0.039 | 0.040 | 0.038 | 0.036 | 0.032 | 0.031 | 0.036 |
| **Change in aortic peak velocity over tertiles** | 0.030 | 0.036 | 0.036 | 0.035 | 0.038 | 0.038 | 0.036 |
| **Aortic valve area** | 0.031 | 0.029 | 0.031 | 0.030 | 0.030 | 0.032 | 0.030 |
| **Aorta ascending** | 0.032 | 0.031 | 0.028 | 0.026 | 0.027 | 0.030 | 0.029 |
| **Platelets** | 0.021 | 0.024 | 0.026 | 0.025 | 0.029 | 0.033 | 0.026 |
| **Age** | 0.023 | 0.024 | 0.024 | 0.026 | 0.024 | 0.022 | 0.024 |
| **AVA index** | 0.025 | 0.021 | 0.022 | 0.022 | 0.022 | 0.023 | 0.023 |
| **Left ventricular outflow tract diameter** | 0.024 | 0.022 | 0.022 | 0.021 | 0.021 | 0.021 | 0.022 |
| **Cholesterol total** | 0.019 | 0.020 | 0.021 | 0.019 | 0.022 | 0.019 | 0.020 |
| **moderate AS progression Echo** | 0.019 | 0.020 | 0.018 | 0.018 | 0.016 | 0.016 | 0.018 |
| **Serum creatinine** | 0.018 | 0.017 | 0.016 | 0.018 | 0.017 | 0.018 | 0.017 |
| **C-reactive protein** | 0.017 | 0.017 | 0.017 | 0.015 | 0.016 | 0.014 | 0.016 |
| **Echo heart rate** | 0.016 | 0.016 | 0.015 | 0.017 | 0.015 | 0.015 | 0.015 |
| **Left ventricular posterior wall diastole** | 0.014 | 0.014 | 0.014 | 0.015 | 0.016 | 0.016 | 0.015 |
| **Left ventricular mass indexed to body surface area** | 0.015 | 0.015 | 0.015 | 0.015 | 0.014 | 0.014 | 0.014 |
| **Aortic root diameter** | 0.013 | 0.013 | 0.013 | 0.013 | 0.013 | 0.013 | 0.013 |
| **Fasting insulin level** | 0.012 | 0.013 | 0.013 | 0.013 | 0.012 | 0.015 | 0.013 |
| **Interventricular septum diastole** | 0.014 | 0.013 | 0.013 | 0.012 | 0.012 | 0.013 | 0.013 |
| **Body mass index** | 0.012 | 0.012 | 0.012 | 0.014 | 0.015 | 0.011 | 0.013 |
| **White blood cells** | 0.008 | 0.010 | 0.011 | 0.011 | 0.014 | 0.017 | 0.012 |
| **Left ventricular mass** | 0.012 | 0.011 | 0.012 | 0.012 | 0.012 | 0.012 | 0.012 |
| **Red globules** | 0.008 | 0.010 | 0.011 | 0.013 | 0.014 | 0.014 | 0.012 |
| **Collagen-adenosine interaction time** | 0.008 | 0.010 | 0.011 | 0.012 | 0.013 | 0.014 | 0.011 |
| **Serum phosphate** | 0.012 | 0.008 | 0.010 | 0.012 | 0.010 | 0.011 | 0.011 |
| **Mitral valve A wave** | 0.012 | 0.011 | 0.010 | 0.009 | 0.009 | 0.010 | 0.010 |
| **Left ventricular dimension in diastole** | 0.009 | 0.010 | 0.010 | 0.011 | 0.009 | 0.008 | 0.010 |
| **Mean glomerular volume** | 0.007 | 0.008 | 0.009 | 0.010 | 0.011 | 0.013 | 0.010 |

**AREA UNDER THE CURVE (AUC) RESULTS**

**Table 2Abis**: AUC values with 95% Confidence Intervals (CI) rounded at 2 digits for XGBoost, GRU, Logistic Regression, LightGBM, LSTM, Naïve Bayes models and the clinical model in predicting clinical outcomes at different visit intervals over a 5-year term using the non-cumulative approach. This table is the table 2A in the main manuscript.

|  | **Prediction at 5 years term** | | | | | | |
| --- | --- | --- | --- | --- | --- | --- | --- |
| **Visit** | **XGboost** | **GRU** | **LogisticRegression** | **LightGBM** | **LSTM** | **Naïve Bayes** | **Clinical model** |
| Baseline | **0.80 (CI: 0.79-0.81)** | 0.73 (CI: 0.72-0.74) | 0.73 (CI: 0.72-0.75) | **0.80 (CI: 0.79-0.81)** | 0.72 (CI: 0.71-0.74) | 0.72 (CI: 0.70-0.73) | 0.70 (CI: 0.69-0.72) |
| 1-year | 0.83 (CI: 0.82-0.84) | 0.82 (CI: 0.81-0.83) | 0.80 (CI: 0.79-0.81) | **0.84 (CI: 0.83-0.85)** | 0.81 (CI: 0.80-0.82) | 0.79 (CI: 0.78-0.80) | 0.71 (CI: 0.70-0.73) |
| 2-year | 0.83 (CI: 0.82-0.84) | 0.82 (CI: 0.81-0.83) | 0.80 (CI: 0.79-0.82) | **0.84 (CI: 0.83-0.85)** | 0.81 (CI: 0.80-0.82) | 0.77 (CI: 0.76-0.79) | 0.72 (CI: 0.71-0.74) |
| 3-year | 0.84 (CI: 0.83-0.86) | 0.82 (CI: 0.81-0.84) | 0.79 (CI: 0.78-0.81) | **0.85 (CI: 0.83-0.86)** | 0.81 (CI: 0.80-0.83) | 0.77 (CI: 0.75-0.78) | 0.66 (CI: 0.64-0.67) |
| 4-year | 0.79 (CI: 0.77-0.81) | 0.78 (CI: 0.76-0.80) | 0.75 (CI: 0.73-0.78) | **0.80 (CI: 0.78-0.81)** | 0.78 (CI: 0.76-0.80) | 0.76 (CI: 0.74-0.77) | 0.64 (CI: 0.62-0.65) |
| 5-year | 0.81 (CI: 0.78-0.83) | 0.82 (CI: 0.80-0.84) | 0.81 (CI: 0.79-0.84) | **0.83 (CI: 0.80-0.85)** | 0.82 (CI: 0.79-0.84) | 0.72 (CI: 0.69-0.75) | 0.58 (CI: 0.57-0.60) |
| Average | 0.82 (CI: 0.80-0.83) | 0.80 (CI: 0.79-0.81) | 0.78 (CI: 0.77-0.80) | **0.83 (CI: 0.81-0.84)** | 0.79 (CI: 0.78-0.81) | 0.76 (CI: 0.74-0.77) | 0.67 (CI: 0.66-0.69) |

**Table 2Abis**: AUC values with 95% Confidence Intervals (CI) rounded at 2 digits for XGBoost, GRU, Logistic Regression, LightGBM, LSTM, Naïve Bayes models and the clinical model in predicting clinical outcomes at different visit intervals over a 2-year term using the non-cumulative approach. This table is the table 2B in the main manuscript.

|  | **Prediction at 2 years term** | | | | | | |
| --- | --- | --- | --- | --- | --- | --- | --- |
| **Visit** | **XGboost** | **GRU** | **LogisticRegression** | **LightGBM** | **LSTM** | **Naïve Bayes** | **Clinical model** |
| Baseline | 0.73 (CI: 0.72-0.74) | 0.66 (CI: 0.64-0.67) | 0.67 (CI: 0.65-0.68) | **0.74 (CI: 0.72-0.75)** | 0.65 (CI: 0.64-0.67) | 0.61 (CI: 0.60-0.63) | 0.64 (CI: 0.62-0.65) |
| 1-year | 0.83 (CI: 0.82-0.84) | 0.81 (CI: 0.79-0.82) | 0.79 (CI: 0.78-0.80) | **0.84 (CI: 0.83-0.85)** | 0.80 (CI: 0.79-0.81) | 0.75 (CI: 0.74-0.77) | 0.72 (CI: 0.71-0.74) |
| 2-year | 0.86 (CI: 0.85-0.87) | 0.85 (CI: 0.83-0.86) | 0.82 (CI: 0.81-0.83) | **0.87 (CI: 0.85-0.88)** | 0.84 (CI: 0.83-0.85) | 0.76 (CI: 0.74-0.77) | 0.72 (CI: 0.71-0.73) |
| 3-year | 0.88 (CI: 0.87-0.90) | 0.85 (CI: 0.84-0.87) | 0.84 (CI: 0.82-0.85) | **0.89 (CI: 0.88-0.90)** | 0.85 (CI: 0.84-0.87) | 0.73 (CI: 0.71-0.76) | 0.70 (CI: 0.68-0.71) |
| 4-year | 0.88 (CI: 0.86-0.89) | 0.86 (CI: 0.84-0.87) | 0.79 (CI: 0.77-0.81) | **0.88 (CI: 0.87-0.90)** | 0.86 (CI: 0.84-0.88) | 0.73 (CI: 0.71-0.75) | 0.66 (CI: 0.65-0.68) |
| 5-year | 0.85 (CI: 0.83-0.87) | 0.83 (CI: 0.81-0.85) | 0.77 (CI: 0.74-0.80) | **0.85 (CI: 0.83-0.87)** | 0.83 (CI: 0.81-0.85) | 0.73 (CI: 0.71-0.76) | 0.59 (CI: 0.58-0.61) |
| Average | 0.84 (CI: 0.83-0.85) | 0.81 (CI: 0.79-0.82) | 0.78 (CI: 0.76-0.80) | **0.85 (CI: 0.83-0.86)** | 0.81 (CI: 0.79-0.82) | 0.72 (CI: 0.70-0.74) | 0.67 (CI: 0.66-0.69) |

**Table S3A**: AUC values with 95% Confidence Intervals (CI) rounded at 2 digits for XGBoost, GRU, Logistic Regression, LightGBM, LSTM, Naïve Bayes models and the clinical model in predicting clinical outcomes at different visit intervals over a 5-year term using the cumulative approach.

|  | **Prediction at 5 years term** | | | | | | |
| --- | --- | --- | --- | --- | --- | --- | --- |
| **Visit** | **XGboost** | **GRU** | **LogisticRegression** | **LightGBM** | **LSTM** | **Naïve Bayes** | **Clinical model** |
| Baseline | **0.80 (CI: 0.79-0.81)** | 0.73 (CI: 0.72-0.74) | 0.72 (CI: 0.71-0.73) | 0.78 (CI: 0.77-0.80) | 0.72 (CI: 0.71-0.74) | 0.63 (CI: 0.61-0.64) | 0.69 (CI: 0.68-0.71) |
| 1-year | **0.85 (CI: 0.84-0.86)** | 0.82 (CI: 0.81-0.83) | 0.79 (CI: 0.78-0.80) | 0.84 (CI: 0.83-0.85) | 0.81 (CI: 0.80-0.82) | 0.70 (CI: 0.69-0.72) | 0.73 (CI: 0.71-0.74) |
| 2-year | **0.84 (CI: 0.83-0.85)** | 0.82 (CI: 0.81-0.83) | 0.81 (CI: 0.79-0.82) | **0.84 (CI: 0.83-0.85)** | 0.81 (CI: 0.80-0.82) | 0.70 (CI: 0.68-0.71) | 0.72 (CI: 0.71-0.73) |
| 3-year | 0.81 (CI: 0.79-0.82) | 0.82 (CI: 0.81-0.84) | 0.76 (CI: 0.75-0.78) | **0.81 (CI: 0.80-0.83)** | 0.81 (CI: 0.80-0.83) | 0.66 (CI: 0.64-0.68) | 0.71 (CI: 0.69-0.73) |
| 4-year | 0.75 (CI: 0.73-0.77) | **0.78 (CI: 0.76-0.80)** | 0.76 (CI: 0.74-0.78) | 0.75 (CI: 0.73-0.77) | **0.78 (CI: 0.76-0.80)** | 0.59 (CI: 0.57-0.61) | 0.71 (CI: 0.69-0.73) |
| 5-year | 0.81 (CI: 0.79-0.84) | 0.82 (CI: 0.80-0.84) | **0.84 (CI: 0.81-0.86)** | 0.81 (CI: 0.79-0.84) | 0.82 (CI: 0.79-0.84) | 0.52 (CI: 0.50-0.55) | 0.69 (CI: 0.66-0.72) |
| Average | **0.81 (CI: 0.80-0.83)** | 0.80 (CI: 0.79-0.81) | 0.78 (CI: 0.76-0.80) | 0.81 (CI: 0.79-0.82) | 0.79 (CI: 0.78-0.81) | 0.63 (CI: 0.62-0.65) | 0.71 (CI: 0.69-0.73) |

**Table S3B**: AUC values with 95% Confidence Intervals (CI) rounded at 2 digits for XGBoost, GRU, Logistic Regression, LightGBM, LSTM, Naïve Bayes models and the clinical model in predicting clinical outcomes at different visit intervals over a 2-year term using the cumulative approach.

|  | **Prediction at 2 years term** | | | | | | |
| --- | --- | --- | --- | --- | --- | --- | --- |
| **Visit** | **XGboost** | **GRU** | **LogisticRegression** | **LightGBM** | **LSTM** | **Naïve Bayes** | **Clinical model** |
| Baseline | 0.69 (CI: 0.67-0.70) | 0.66 (CI: 0.64-0.67) | 0.64 (CI: 0.62-0.65) | **0.69 (CI: 0.68-0.70)** | 0.65 (CI: 0.64-0.67) | 0.50 (CI: 0.48-0.51) | 0.61 (CI: 0.60-0.63) |
| 1-year | **0.85 (CI: 0.84-0.86)** | 0.81 (CI: 0.79-0.82) | 0.82 (CI: 0.80-0.83) | **0.85 (CI: 0.84-0.86)** | 0.80 (CI: 0.79-0.81) | 0.67 (CI: 0.66-0.69) | 0.70 (CI: 0.68-0.71) |
| 2-year | **0.88 (CI: 0.87-0.89)** | 0.85 (CI: 0.83-0.86) | 0.85 (CI: 0.84-0.87) | **0.88 (CI: 0.87-0.89)** | 0.84 (CI: 0.83-0.85) | 0.67 (CI: 0.65-0.69) | 0.70 (CI: 0.68-0.71) |
| 3-year | 0.89 (CI: 0.87-0.90) | 0.85 (CI: 0.84-0.87) | 0.86 (CI: 0.85-0.87) | **0.89 (CI: 0.88-0.91)** | 0.85 (CI: 0.84-0.87) | 0.61 (CI: 0.59-0.63) | 0.73 (CI: 0.72-0.75) |
| 4-year | **0.90 (CI: 0.89-0.91)** | 0.86 (CI: 0.84-0.87) | 0.82 (CI: 0.80-0.84) | 0.89 (CI: 0.88-0.91) | 0.86 (CI: 0.84-0.88) | 0.56 (CI: 0.54-0.59) | 0.77 (CI: 0.75-0.79) |
| 5-year | 0.78 (CI: 0.76-0.81) | 0.83 (CI: 0.81-0.85) | 0.83 (CI: 0.80-0.85) | 0.79 (CI: 0.77-0.82) | **0.83 (CI: 0.81-0.85)** | 0.49 (CI: 0.47-0.52) | 0.67 (CI: 0.64-0.70) |
| Average | **0.83 (CI: 0.82-0.85)** | 0.81 (CI: 0.79-0.82) | 0.80 (CI: 0.79-0.82) | **0.83 (CI: 0.82-0.85)** | 0.81 (CI: 0.79-0.82) | 0.58 (CI: 0.57-0.61) | 0.70 (CI: 0.68-0.72) |

**Table S3C**: AUC values with 95% Confidence Intervals (CI) rounded at 2 digits for XGBoost, GRU, Logistic Regression, LightGBM, LSTM, Naïve Bayes models and the clinical model in predicting clinical outcomes of the population strictly above 68 years old (median age of the cohort) at different visit intervals over a 5-year term using the non-cumulative approach.

|  | **Prediction at 5 years term** | | | | | | |
| --- | --- | --- | --- | --- | --- | --- | --- |
| **Visit** | **XGboost** | **GRU** | **LogisticRegression** | **LightGBM** | **LSTM** | **Naïve Bayes** | **Clinical model** |
| Baseline | **0.77 (CI: 0.75-0.80)** | 0.70 (CI: 0.67-0.72) | 0.69 (CI: 0.67-0.72) | 0.77 (CI: 0.75-0.79) | 0.69 (CI: 0.67-0.71) | 0.65 (CI: 0.63-0.68) | 0.67 (CI: 0.64-0.69) |
| 1-year | 0.83 (CI: 0.81-0.85) | 0.81 (CI: 0.79-0.82) | 0.78 (CI: 0.76-0.80) | **0.84 (CI: 0.82-0.85)** | 0.79 (CI: 0.77-0.81) | 0.77 (CI: 0.75-0.79) | 0.71 (CI: 0.69-0.73) |
| 2-year | 0.82 (CI: 0.81-0.84) | 0.80 (CI: 0.78-0.82) | 0.78 (CI: 0.76-0.81) | **0.84 (CI: 0.82-0.86)** | 0.80 (CI: 0.78-0.81) | 0.75 (CI: 0.72-0.77) | 0.69 (CI: 0.67-0.72) |
| 3-year | **0.86 (CI: 0.84-0.88)** | 0.81 (CI: 0.79-0.83) | 0.78 (CI: 0.75-0.81) | **0.86 (CI: 0.84-0.88)** | 0.80 (CI: 0.78-0.83) | 0.74 (CI: 0.71-0.77) | 0.65 (CI: 0.62-0.69) |
| 4-year | 0.75 (CI: 0.72-0.79) | 0.72 (CI: 0.69-0.76) | 0.69 (CI: 0.65-0.73) | **0.76 (CI: 0.72-0.79)** | 0.72 (CI: 0.69-0.76) | 0.66 (CI: 0.62-0.69) | 0.68 (CI: 0.65-0.71) |
| 5-year | **0.78 (CI: 0.73-0.83)** | 0.73 (CI: 0.68-0.79) | 0.74 (CI: 0.69-0.79) | **0.78 (CI: 0.73-0.83)** | 0.73 (CI: 0.67-0.78) | 0.62 (CI: 0.57-0.68) | 0.71 (CI: 0.66-0.75) |
| Average | 0.80 (CI: 0.78-0.83) | 0.76 (CI: 0.73-0.79) | 0.74 (CI: 0.71-0.78) | **0.81 (CI: 0.78-0.83)** | 0.76 (CI: 0.73-0.78) | 0.70 (CI: 0.67-0.73) | 0.69 (CI: 0.66-0.72) |

**Table S3D**: AUC values with 95% Confidence Intervals (CI) rounded at 2 digits for XGBoost, GRU, Logistic Regression, LightGBM, LSTM, Naïve Bayes models and the clinical model in predicting clinical outcomes of the population strictly above 68 years old (median age of the cohort) at different visit intervals over a 2-year term using the non-cumulative approach.

|  | **Prediction at 2 years term** | | | | | | |
| --- | --- | --- | --- | --- | --- | --- | --- |
| **Visit** | **XGboost** | **GRU** | **LogisticRegression** | **LightGBM** | **LSTM** | **Naïve Bayes** | **Clinical model** |
| Baseline | 0.73 (CI: 0.70-0.75) | 0.67 (CI: 0.65-0.70) | 0.65 (CI: 0.63-0.68) | **0.73 (CI: 0.71-0.75)** | 0.68 (CI: 0.66-0.70) | 0.59 (CI: 0.57-0.62) | 0.60 (CI: 0.57-0.63) |
| 1-year | 0.83 (CI: 0.81-0.85) | 0.80 (CI: 0.78-0.82) | 0.76 (CI: 0.74-0.78) | **0.85 (CI: 0.83-0.86)** | 0.80 (CI: 0.78-0.82) | 0.76 (CI: 0.74-0.78) | 0.73 (CI: 0.71-0.75) |
| 2-year | **0.88 (CI: 0.86-0.90)** | 0.85 (CI: 0.83-0.87) | 0.80 (CI: 0.77-0.82) | 0.87 (CI: 0.85-0.89) | 0.84 (CI: 0.83-0.86) | 0.76 (CI: 0.74-0.78) | 0.72 (CI: 0.70-0.74) |
| 3-year | 0.89 (CI: 0.87-0.91) | 0.86 (CI: 0.84-0.88) | 0.84 (CI: 0.82-0.86) | **0.90 (CI: 0.88-0.92)** | 0.86 (CI: 0.84-0.88) | 0.76 (CI: 0.73-0.78) | 0.75 (CI: 0.72-0.78) |
| 4-year | **0.84 (CI: 0.81-0.86)** | 0.81 (CI: 0.78-0.85) | 0.68 (CI: 0.64-0.72) | 0.83 (CI: 0.81-0.86) | 0.81 (CI: 0.78-0.85) | 0.65 (CI: 0.62-0.69) | 0.75 (CI: 0.70-0.79) |
| 5-year | 0.84 (CI: 0.81-0.88) | 0.79 (CI: 0.75-0.84) | 0.72 (CI: 0.67-0.77) | **0.85 (CI: 0.81-0.88)** | 0.81 (CI: 0.76-0.85) | 0.65 (CI: 0.59-0.70) | 0.72 (CI: 0.67-0.79) |
| Average | 0.84 (CI: 0.81-0.86) | 0.80 (CI: 0.77-0.83) | 0.74 (CI: 0.71-0.77) | **0.84 (CI: 0.82-0.86)** | 0.80 (CI: 0.78-0.83) | 0.70 (CI: 0.67-0.73) | 0.71 (CI: 0.68-0.75) |

**Table S3E**: AUC values with 95% Confidence Intervals (CI) rounded at 2 digits for XGBoost, GRU, Logistic Regression, LightGBM, LSTM, Naïve Bayes models and the clinical model in predicting clinical outcomes of the population under or equal to 68 years old (median age of the cohort) at different visit intervals over a 5-year term using the non-cumulative approach.

|  | **Prediction at 5 years term** | | | | | | |
| --- | --- | --- | --- | --- | --- | --- | --- |
| **Visit** | **XGboost** | **GRU** | **LogisticRegression** | **LightGBM** | **LSTM** | **Naïve Bayes** | **Clinical model** |
| Baseline | 0.81 (CI: 0.80-0.83) | 0.76 (CI: 0.74-0.77) | 0.76 (CI: 0.74-0.78) | **0.82 (CI: 0.81-0.83)** | 0.75 (CI: 0.73-0.77) | 0.77 (CI: 0.75-0.79) | 0.73 (CI: 0.72-0.75) |
| 1-year | 0.83 (CI: 0.82-0.85) | 0.83 (CI: 0.82-0.85) | 0.82 (CI: 0.81-0.84) | **0.84 (CI: 0.83-0.86)** | 0.83 (CI: 0.81-0.85) | 0.82 (CI: 0.80-0.84) | 0.71 (CI: 0.69-0.73) |
| 2-year | 0.83 (CI: 0.82-0.85) | 0.83 (CI: 0.82-0.85) | 0.82 (CI: 0.81-0.84) | **0.84 (CI: 0.82-0.86)** | 0.83 (CI: 0.81-0.85) | 0.81 (CI: 0.79-0.83) | 0.71 (CI: 0.69-0.74) |
| 3-year | 0.82 (CI: 0.80-0.85) | **0.83 (CI: 0.81-0.86)** | 0.81 (CI: 0.78-0.83) | 0.83 (CI: 0.81-0.85) | 0.83 (CI: 0.81-0.85) | 0.80 (CI: 0.77-0.83) | 0.60 (CI: 0.56-0.64) |
| 4-year | 0.83 (CI: 0.81-0.86) | 0.84 (CI: 0.81-0.87) | 0.81 (CI: 0.78-0.84) | 0.83 (CI: 0.80-0.86) | 0.83 (CI: 0.81-0.86) | **0.86 (CI: 0.84-0.89)** | 0.68 (CI: 0.63-0.72) |
| 5-year | 0.83 (CI: 0.80-0.87) | **0.89 (CI: 0.86-0.92)** | 0.87 (CI: 0.84-0.90) | 0.87 (CI: 0.83-0.90) | 0.88 (CI: 0.85-0.91) | 0.82 (CI: 0.78-0.86) | 0.67 (CI: 0.62-0.72) |
| Average | 0.83 (CI: 0.81-0.85) | 0.83 (CI: 0.81-0.85) | 0.82 (CI: 0.79-0.84) | **0.84 (CI: 0.82-0.86)** | 0.83 (CI: 0.80-0.85) | 0.81 (CI: 0.79-0.84) | 0.68 (CI: 0.65-0.72) |

**Table S3F**: AUC values with 95% Confidence Intervals (CI) rounded at 2 digits for XGBoost, GRU, Logistic Regression, LightGBM, LSTM, Naïve Bayes models and the clinical model in predicting clinical outcomes of the population under or equal to 68 years old (median age of the cohort) at different visit intervals over a 2-year term using the non-cumulative approach.

|  | **Prediction at 2 years term** | | | | | | |
| --- | --- | --- | --- | --- | --- | --- | --- |
| **Visit** | **XGboost** | **GRU** | **LogisticRegression** | **LightGBM** | **LSTM** | **Naïve Bayes** | **Clinical model** |
| Baseline | 0.74 (CI: 0.72-0.76) | 0.65 (CI: 0.62-0.67) | 0.68 (CI: 0.66-0.70) | **0.75 (CI: 0.73-0.77)** | 0.64 (CI: 0.61-0.66) | 0.63 (CI: 0.61-0.65) | 0.67 (CI: 0.65-0.69) |
| 1-year | 0.83 (CI: 0.82-0.85) | 0.81 (CI: 0.79-0.82) | 0.82 (CI: 0.80-0.84) | **0.84 (CI: 0.82-0.86)** | 0.80 (CI: 0.78-0.82) | 0.75 (CI: 0.72-0.77) | 0.71 (CI: 0.69-0.73) |
| 2-year | 0.85 (CI: 0.83-0.87) | 0.85 (CI: 0.83-0.87) | 0.84 (CI: 0.82-0.86) | **0.86 (CI: 0.84-0.87)** | 0.84 (CI: 0.82-0.86) | 0.76 (CI: 0.74-0.79) | 0.74 (CI: 0.71-0.77) |
| 3-year | 0.87 (CI: 0.86-0.89) | 0.86 (CI: 0.83-0.88) | 0.83 (CI: 0.81-0.86) | **0.88 (CI: 0.86-0.90)** | 0.85 (CI: 0.82-0.87) | 0.73 (CI: 0.70-0.76) | 0.74 (CI: 0.71-0.77) |
| 4-year | 0.91 (CI: 0.89-0.93) | 0.91 (CI: 0.89-0.93) | 0.86 (CI: 0.84-0.89) | **0.92 (CI: 0.90-0.94)** | 0.90 (CI: 0.88-0.92) | 0.82 (CI: 0.79-0.85) | 0.78 (CI: 0.74-0.82) |
| 5-year | 0.85 (CI: 0.82-0.88) | **0.87 (CI: 0.84-0.90)** | 0.82 (CI: 0.79-0.86) | 0.84 (CI: 0.81-0.87) | **0.87 (CI: 0.84-0.90)** | 0.83 (CI: 0.80-0.86) | 0.70 (CI: 0.64-0.77) |
| Average | **0.84 (CI: 0.82-0.86)** | 0.83 (CI: 0.80-0.85) | 0.81 (CI: 0.79-0.84) | **0.85 (CI: 0.83-0.87)** | 0.82 (CI: 0.79-0.84) | 0.75 (CI: 0.73-0.78) | 0.72 (CI: 0.69-0.76) |

**Table S3G**: AUC values with 95% Confidence Intervals (CI) rounded at 2 digits for XGBoost, GRU, Logistic Regression, LightGBM, LSTM, Naïve Bayes models and the clinical model in predicting clinical outcomes of the population strictly above 68 years old (median age of the cohort) at different visit intervals over a 5-year term using the cumulative approach.

|  | **Prediction at 5 years term** | | | | | | |
| --- | --- | --- | --- | --- | --- | --- | --- |
| **Visit** | **XGboost** | **GRU** | **LogisticRegression** | **LightGBM** | **LSTM** | **Naïve Bayes** | **Clinical model** |
| Baseline | **0.76 (CI: 0.74-0.78)** | 0.70 (CI: 0.67-0.72) | 0.68 (CI: 0.66-0.70) | 0.74 (CI: 0.72-0.76) | 0.69 (CI: 0.67-0.71) | 0.57 (CI: 0.55-0.59) | 0.66 (CI: 0.64-0.68) |
| 1-year | **0.83 (CI: 0.81-0.85)** | 0.81 (CI: 0.79-0.82) | 0.76 (CI: 0.74-0.78) | 0.82 (CI: 0.80-0.84) | 0.79 (CI: 0.77-0.81) | 0.66 (CI: 0.64-0.69) | 0.70 (CI: 0.68-0.72) |
| 2-year | **0.81 (CI: 0.79-0.83)** | 0.80 (CI: 0.78-0.82) | 0.79 (CI: 0.76-0.81) | **0.81 (CI: 0.79-0.83)** | 0.80 (CI: 0.78-0.81) | 0.66 (CI: 0.64-0.69) | 0.71 (CI: 0.69-0.74) |
| 3-year | 0.81 (CI: 0.78-0.83) | **0.81 (CI: 0.79-0.83)** | 0.73 (CI: 0.70-0.76) | **0.81 (CI: 0.79-0.83)** | 0.80 (CI: 0.78-0.83) | 0.62 (CI: 0.59-0.65) | 0.71 (CI: 0.68-0.73) |
| 4-year | 0.71 (CI: 0.67-0.74) | **0.72 (CI: 0.69-0.76)** | 0.68 (CI: 0.64-0.72) | 0.71 (CI: 0.67-0.75) | **0.72 (CI: 0.69-0.76)** | 0.54 (CI: 0.52-0.57) | 0.66 (CI: 0.62-0.69) |
| 5-year | **0.77 (CI: 0.72-0.81)** | 0.73 (CI: 0.68-0.79) | 0.73 (CI: 0.68-0.79) | 0.74 (CI: 0.69-0.80) | 0.73 (CI: 0.67-0.78) | 0.49 (CI: 0.45-0.52) | 0.66 (CI: 0.61-0.71) |
| Average | **0.78 (CI: 0.75-0.81)** | 0.76 (CI: 0.73-0.79) | 0.73 (CI: 0.70-0.76) | 0.77 (CI: 0.74-0.80) | 0.76 (CI: 0.73-0.78) | 0.59 (CI: 0.57-0.62) | 0.68 (CI: 0.65-0.71) |

**Table S3H**: AUC values with 95% Confidence Intervals (CI) rounded at 2 digits for XGBoost, GRU, Logistic Regression, LightGBM, LSTM, Naïve Bayes models and the clinical model in predicting clinical outcomes of the population strictly above 68 years old (median age of the cohort) at different visit intervals over a 2-year term using the cumulative approach.

|  | **Prediction at 2 years term** | | | | | | |
| --- | --- | --- | --- | --- | --- | --- | --- |
| **Visit** | **XGboost** | **GRU** | **LogisticRegression** | **LightGBM** | **LSTM** | **Naïve Bayes** | **Clinical model** |
| Baseline | 0.67 (CI: 0.64-0.69) | 0.67 (CI: 0.65-0.70) | 0.61 (CI: 0.59-0.64) | 0.67 (CI: 0.65-0.70) | **0.68 (CI: 0.66-0.70)** | 0.48 (CI: 0.46-0.50) | 0.59 (CI: 0.56-0.62) |
| 1-year | 0.84 (CI: 0.82-0.86) | 0.80 (CI: 0.78-0.82) | 0.79 (CI: 0.78-0.81) | **0.84 (CI: 0.83-0.86)** | 0.80 (CI: 0.78-0.82) | 0.65 (CI: 0.63-0.67) | 0.70 (CI: 0.68-0.72) |
| 2-year | **0.88 (CI: 0.87-0.90)** | 0.85 (CI: 0.83-0.87) | 0.87 (CI: 0.85-0.89) | 0.88 (CI: 0.86-0.90) | 0.84 (CI: 0.83-0.86) | 0.64 (CI: 0.61-0.66) | 0.74 (CI: 0.72-0.76) |
| 3-year | 0.90 (CI: 0.88-0.91) | 0.86 (CI: 0.84-0.88) | 0.85 (CI: 0.83-0.87) | **0.90 (CI: 0.88-0.92)** | 0.86 (CI: 0.84-0.88) | 0.62 (CI: 0.59-0.65) | 0.74 (CI: 0.71-0.77) |
| 4-year | **0.86 (CI: 0.83-0.89)** | 0.81 (CI: 0.78-0.85) | 0.77 (CI: 0.74-0.81) | 0.84 (CI: 0.82-0.87) | 0.81 (CI: 0.78-0.85) | 0.51 (CI: 0.47-0.54) | 0.65 (CI: 0.61-0.69) |
| 5-year | 0.76 (CI: 0.72-0.81) | 0.79 (CI: 0.75-0.84) | 0.78 (CI: 0.73-0.82) | 0.77 (CI: 0.72-0.82) | **0.81 (CI: 0.76-0.85)** | 0.48 (CI: 0.45-0.52) | 0.58 (CI: 0.53-0.63) |
| Average | 0.82 (CI: 0.79-0.84) | 0.80 (CI: 0.77-0.83) | 0.78 (CI: 0.75-0.81) | **0.82 (CI: 0.79-0.85)** | 0.80 (CI: 0.78-0.83) | 0.56 (CI: 0.54-0.59) | 0.67 (CI: 0.64-0.70) |

**Table S3I**: AUC values with 95% Confidence Intervals (CI) rounded at 2 digits for XGBoost, GRU, Logistic Regression, LightGBM, LSTM, Naïve Bayes models and the clinical model in predicting clinical outcomes of the population under or equal to 68 years old (median age of the cohort) at different visit intervals over a 5-year term using the cumulative approach.

|  | **Prediction at 5 years term** | | | | | | |
| --- | --- | --- | --- | --- | --- | --- | --- |
| **Visit** | **XGboost** | **GRU** | **LogisticRegression** | **LightGBM** | **LSTM** | **Naïve Bayes** | **Clinical model** |
| Baseline | **0.83 (CI: 0.81-0.84)** | 0.76 (CI: 0.74-0.77) | 0.75 (CI: 0.73-0.77) | 0.81 (CI: 0.80-0.83) | 0.75 (CI: 0.73-0.77) | 0.66 (CI: 0.64-0.69) | 0.73 (CI: 0.71-0.75) |
| 1-year | **0.86 (CI: 0.85-0.88)** | 0.83 (CI: 0.82-0.85) | 0.82 (CI: 0.80-0.84) | 0.86 (CI: 0.84-0.87) | 0.83 (CI: 0.81-0.85) | 0.74 (CI: 0.72-0.76) | 0.74 (CI: 0.72-0.77) |
| 2-year | **0.87 (CI: 0.85-0.89)** | 0.83 (CI: 0.82-0.85) | 0.83 (CI: 0.81-0.84) | **0.87 (CI: 0.85-0.89)** | 0.83 (CI: 0.81-0.85) | 0.73 (CI: 0.71-0.76) | 0.72 (CI: 0.70-0.75) |
| 3-year | 0.80 (CI: 0.77-0.82) | **0.83 (CI: 0.81-0.86)** | 0.79 (CI: 0.77-0.82) | 0.81 (CI: 0.78-0.83) | 0.83 (CI: 0.81-0.85) | 0.69 (CI: 0.66-0.72) | 0.71 (CI: 0.69-0.74) |
| 4-year | 0.79 (CI: 0.76-0.83) | 0.84 (CI: 0.81-0.87) | **0.84 (CI: 0.82-0.87)** | 0.79 (CI: 0.75-0.82) | 0.83 (CI: 0.81-0.86) | 0.63 (CI: 0.60-0.66) | 0.77 (CI: 0.74-0.80) |
| 5-year | 0.83 (CI: 0.79-0.87) | 0.89 (CI: 0.86-0.92) | **0.90 (CI: 0.88-0.93)** | 0.83 (CI: 0.79-0.87) | 0.88 (CI: 0.85-0.91) | 0.56 (CI: 0.53-0.59) | 0.73 (CI: 0.69-0.77) |
| Average | **0.83 (CI: 0.81-0.86)** | 0.83 (CI: 0.81-0.85) | 0.82 (CI: 0.80-0.85) | 0.83 (CI: 0.80-0.85) | 0.83 (CI: 0.80-0.85) | 0.67 (CI: 0.64-0.70) | 0.73 (CI: 0.71-0.76) |

**Table S3J**: AUC values with 95% Confidence Intervals (CI) rounded at 2 digits for XGBoost, GRU, Logistic Regression, LightGBM, LSTM, Naïve Bayes models and the clinical model in predicting clinical outcomes of the population under or equal to 68 years old (median age of the cohort) at different visit intervals over a 2-year term using the cumulative approach.

|  | **Prediction at 2 years term** | | | | | | |
| --- | --- | --- | --- | --- | --- | --- | --- |
| **Visit** | **XGboost** | **GRU** | **LogisticRegression** | **LightGBM** | **LSTM** | **Naïve Bayes** | **Clinical model** |
| Baseline | **0.71 (CI: 0.69-0.73)** | 0.65 (CI: 0.62-0.67) | 0.66 (CI: 0.64-0.69) | **0.71 (CI: 0.69-0.73)** | 0.64 (CI: 0.61-0.66) | 0.50 (CI: 0.48-0.52) | 0.64 (CI: 0.62-0.66) |
| 1-year | **0.86 (CI: 0.84-0.87)** | 0.81 (CI: 0.79-0.82) | 0.83 (CI: 0.81-0.84) | 0.85 (CI: 0.84-0.87) | 0.80 (CI: 0.78-0.82) | 0.68 (CI: 0.66-0.70) | 0.69 (CI: 0.67-0.71) |
| 2-year | **0.88 (CI: 0.87-0.90)** | 0.85 (CI: 0.83-0.87) | 0.84 (CI: 0.82-0.86) | 0.88 (CI: 0.86-0.89) | 0.84 (CI: 0.82-0.86) | 0.70 (CI: 0.68-0.73) | 0.65 (CI: 0.63-0.68) |
| 3-year | 0.87 (CI: 0.84-0.89) | 0.86 (CI: 0.83-0.88) | 0.87 (CI: 0.85-0.89) | **0.88 (CI: 0.86-0.91)** | 0.85 (CI: 0.82-0.87) | 0.60 (CI: 0.57-0.64) | 0.73 (CI: 0.71-0.76) |
| 4-year | **0.93 (CI: 0.92-0.95)** | 0.91 (CI: 0.89-0.93) | 0.87 (CI: 0.84-0.89) | 0.93 (CI: 0.91-0.95) | 0.90 (CI: 0.88-0.92) | 0.62 (CI: 0.58-0.66) | 0.89 (CI: 0.87-0.92) |
| 5-year | 0.80 (CI: 0.77-0.84) | **0.87 (CI: 0.84-0.90)** | **0.87 (CI: 0.84-0.90)** | 0.82 (CI: 0.78-0.85) | **0.87 (CI: 0.84-0.90)** | 0.50 (CI: 0.46-0.54) | 0.74 (CI: 0.70-0.79) |
| Average | **0.84 (CI: 0.82-0.86)** | 0.83 (CI: 0.80-0.85) | 0.82 (CI: 0.80-0.85) | **0.85 (CI: 0.82-0.87)** | 0.82 (CI: 0.79-0.84) | 0.60 (CI: 0.57-0.63) | 0.72 (CI: 0.70-0.75) |

**Table S3K**: AUC values with 95% Confidence Intervals (CI) rounded at 2 digits for XGBoost, GRU, Logistic Regression, LightGBM, LSTM, Naïve Bayes models and the clinical model in predicting clinical outcomes of the men population at different visit intervals over a 5-year term using the non-cumulative approach.

|  | **Prediction at 5 years term** | | | | | | |
| --- | --- | --- | --- | --- | --- | --- | --- |
| **Visit** | **XGboost** | **GRU** | **LogisticRegression** | **LightGBM** | **LSTM** | **Naïve Bayes** | **Clinical model** |
| Baseline | 0.78 (CI: 0.77-0.80) | 0.73 (CI: 0.71-0.74) | 0.72 (CI: 0.70-0.74) | **0.79 (CI: 0.77-0.80)** | 0.72 (CI: 0.71-0.74) | 0.72 (CI: 0.70-0.74) | 0.74 (CI: 0.73-0.76) |
| 1-year | 0.82 (CI: 0.81-0.84) | 0.82 (CI: 0.80-0.83) | 0.80 (CI: 0.78-0.81) | **0.83 (CI: 0.82-0.84)** | 0.81 (CI: 0.79-0.83) | 0.79 (CI: 0.78-0.81) | 0.74 (CI: 0.72-0.76) |
| 2-year | 0.83 (CI: 0.81-0.84) | 0.82 (CI: 0.80-0.83) | 0.80 (CI: 0.79-0.82) | **0.84 (CI: 0.82-0.85)** | 0.81 (CI: 0.79-0.82) | 0.79 (CI: 0.77-0.80) | 0.70 (CI: 0.68-0.72) |
| 3-year | 0.83 (CI: 0.81-0.85) | 0.82 (CI: 0.80-0.84) | 0.80 (CI: 0.78-0.82) | **0.84 (CI: 0.82-0.86)** | 0.81 (CI: 0.79-0.83) | 0.74 (CI: 0.72-0.76) | 0.65 (CI: 0.62-0.67) |
| 4-year | 0.77 (CI: 0.75-0.79) | 0.77 (CI: 0.74-0.79) | 0.74 (CI: 0.72-0.77) | **0.78 (CI: 0.76-0.80)** | 0.77 (CI: 0.74-0.79) | 0.74 (CI: 0.72-0.77) | 0.69 (CI: 0.66-0.71) |
| 5-year | 0.73 (CI: 0.70-0.77) | 0.76 (CI: 0.72-0.79) | 0.75 (CI: 0.71-0.79) | 0.75 (CI: 0.72-0.79) | **0.77 (CI: 0.73-0.81)** | 0.66 (CI: 0.61-0.71) | 0.71 (CI: 0.68-0.75) |
| Average | 0.79 (CI: 0.78-0.82) | 0.79 (CI: 0.76-0.80) | 0.77 (CI: 0.75-0.79) | **0.81 (CI: 0.79-0.82)** | 0.78 (CI: 0.76-0.80) | 0.74 (CI: 0.72-0.77) | 0.71 (CI: 0.68-0.73) |

**Table S3L**: AUC values with 95% Confidence Intervals (CI) rounded at 2 digits for XGBoost, GRU, Logistic Regression, LightGBM, LSTM, Naïve Bayes models and the clinical model in predicting clinical outcomes of the men population at different visit intervals over a 2-year term using the non-cumulative approach.

|  | **Prediction at 2 years term** | | | | | | |
| --- | --- | --- | --- | --- | --- | --- | --- |
| **Visit** | **XGboost** | **GRU** | **LogisticRegression** | **LightGBM** | **LSTM** | **Naïve Bayes** | **Clinical model** |
| Baseline | 0.73 (CI: 0.71-0.75) | 0.66 (CI: 0.64-0.68) | 0.66 (CI: 0.64-0.68) | **0.74 (CI: 0.72-0.76)** | 0.66 (CI: 0.64-0.68) | 0.63 (CI: 0.61-0.65) | 0.66 (CI: 0.64-0.68) |
| 1-year | 0.84 (CI: 0.82-0.85) | 0.81 (CI: 0.80-0.83) | 0.78 (CI: 0.77-0.80) | **0.85 (CI: 0.84-0.86)** | 0.81 (CI: 0.79-0.82) | 0.75 (CI: 0.74-0.77) | 0.73 (CI: 0.71-0.74) |
| 2-year | **0.85 (CI: 0.84-0.87)** | **0.85 (CI: 0.84-0.87)** | 0.81 (CI: 0.80-0.83) | 0.85 (CI: 0.84-0.86) | 0.84 (CI: 0.82-0.85) | 0.76 (CI: 0.74-0.78) | 0.72 (CI: 0.70-0.74) |
| 3-year | 0.88 (CI: 0.87-0.90) | 0.87 (CI: 0.85-0.88) | 0.84 (CI: 0.83-0.86) | **0.89 (CI: 0.88-0.90)** | 0.86 (CI: 0.84-0.88) | 0.72 (CI: 0.69-0.74) | 0.75 (CI: 0.73-0.77) |
| 4-year | 0.87 (CI: 0.85-0.89) | 0.86 (CI: 0.84-0.88) | 0.80 (CI: 0.78-0.82) | **0.88 (CI: 0.86-0.90)** | 0.86 (CI: 0.84-0.88) | 0.72 (CI: 0.70-0.75) | 0.78 (CI: 0.75-0.81) |
| 5-year | **0.81 (CI: 0.78-0.84)** | 0.79 (CI: 0.76-0.82) | 0.72 (CI: 0.68-0.76) | 0.80 (CI: 0.77-0.84) | 0.79 (CI: 0.76-0.82) | 0.65 (CI: 0.62-0.69) | 0.72 (CI: 0.67-0.77) |
| Average | 0.83 (CI: 0.81-0.85) | 0.81 (CI: 0.79-0.83) | 0.77 (CI: 0.75-0.79) | **0.84 (CI: 0.82-0.85)** | 0.80 (CI: 0.78-0.82) | 0.71 (CI: 0.68-0.73) | 0.73 (CI: 0.70-0.75) |

**Table S3M**: AUC values with 95% Confidence Intervals (CI) rounded at 2 digits for XGBoost, GRU, Logistic Regression, LightGBM, LSTM, Naïve Bayes models and the clinical model in predicting clinical outcomes of the women population at different visit intervals over a 5-year term using the non-cumulative approach.

|  | **Prediction at 5 years term** | | | | | | |
| --- | --- | --- | --- | --- | --- | --- | --- |
| **Visit** | **XGboost** | **GRU** | **LogisticRegression** | **LightGBM** | **LSTM** | **Naïve Bayes** | **Clinical model** |
| Baseline | **0.84 (CI: 0.82-0.87)** | 0.75 (CI: 0.71-0.78) | 0.75 (CI: 0.72-0.78) | **0.84 (CI: 0.82-0.87)** | 0.74 (CI: 0.71-0.77) | 0.71 (CI: 0.68-0.74) | 0.61 (CI: 0.58-0.65) |
| 1-year | **0.87 (CI: 0.85-0.89)** | 0.83 (CI: 0.80-0.86) | 0.82 (CI: 0.79-0.85) | **0.87 (CI: 0.85-0.89)** | 0.81 (CI: 0.79-0.84) | 0.78 (CI: 0.76-0.81) | 0.67 (CI: 0.63-0.70) |
| 2-year | 0.84 (CI: 0.82-0.87) | 0.84 (CI: 0.81-0.87) | 0.80 (CI: 0.76-0.83) | **0.85 (CI: 0.83-0.87)** | 0.83 (CI: 0.81-0.86) | 0.73 (CI: 0.70-0.77) | 0.69 (CI: 0.65-0.74) |
| 3-year | 0.85 (CI: 0.82-0.89) | 0.83 (CI: 0.80-0.87) | 0.77 (CI: 0.73-0.81) | **0.86 (CI: 0.83-0.90)** | 0.82 (CI: 0.79-0.85) | 0.82 (CI: 0.78-0.86) | 0.61 (CI: 0.55-0.67) |
| 4-year | **0.86 (CI: 0.82-0.90)** | 0.82 (CI: 0.78-0.87) | 0.79 (CI: 0.75-0.84) | 0.84 (CI: 0.80-0.88) | 0.81 (CI: 0.76-0.86) | 0.84 (CI: 0.80-0.88) | 0.63 (CI: 0.58-0.69) |
| 5-year | **0.97 (CI: 0.95-1.00)** | 0.91 (CI: 0.87-0.95) | 0.91 (CI: 0.87-0.95) | 0.97 (CI: 0.94-1.00) | 0.91 (CI: 0.87-0.96) | 0.83 (CI: 0.78-0.89) | 0.66 (CI: 0.59-0.74) |
| Average | **0.87 (CI: 0.85-0.90)** | 0.83 (CI: 0.80-0.87) | 0.81 (CI: 0.77-0.84) | **0.87 (CI: 0.85-0.90)** | 0.82 (CI: 0.79-0.86) | 0.79 (CI: 0.75-0.83) | 0.65 (CI: 0.60-0.70) |

**Table S3N**: AUC values with 95% Confidence Intervals (CI) rounded at 2 digits for XGBoost, GRU, Logistic Regression, LightGBM, LSTM, Naïve Bayes models and the clinical model in predicting clinical outcomes of the women population at different visit intervals over a 2-year term using the non-cumulative approach.

|  | **Prediction at 2 years term** | | | | | | |
| --- | --- | --- | --- | --- | --- | --- | --- |
| **Visit** | **XGboost** | **GRU** | **LogisticRegression** | **LightGBM** | **LSTM** | **Naïve Bayes** | **Clinical model** |
| Baseline | 0.73 (CI: 0.70-0.76) | 0.65 (CI: 0.61-0.69) | 0.69 (CI: 0.65-0.72) | **0.73 (CI: 0.70-0.77)** | 0.64 (CI: 0.60-0.68) | 0.54 (CI: 0.50-0.58) | 0.60 (CI: 0.57-0.64) |
| 1-year | 0.82 (CI: 0.80-0.85) | 0.79 (CI: 0.76-0.82) | 0.80 (CI: 0.78-0.83) | **0.83 (CI: 0.81-0.85)** | 0.78 (CI: 0.75-0.81) | 0.75 (CI: 0.72-0.78) | 0.77 (CI: 0.74-0.80) |
| 2-year | 0.90 (CI: 0.88-0.92) | 0.84 (CI: 0.80-0.87) | 0.83 (CI: 0.80-0.86) | **0.91 (CI: 0.88-0.93)** | 0.83 (CI: 0.79-0.86) | 0.75 (CI: 0.71-0.78) | 0.75 (CI: 0.71-0.79) |
| 3-year | 0.88 (CI: 0.86-0.91) | 0.81 (CI: 0.78-0.85) | 0.80 (CI: 0.77-0.84) | **0.89 (CI: 0.87-0.92)** | 0.83 (CI: 0.80-0.87) | 0.80 (CI: 0.76-0.84) | 0.75 (CI: 0.70-0.79) |
| 4-year | **0.90 (CI: 0.87-0.94)** | 0.86 (CI: 0.82-0.90) | 0.79 (CI: 0.74-0.84) | 0.90 (CI: 0.86-0.94) | 0.87 (CI: 0.84-0.91) | 0.80 (CI: 0.76-0.85) | 0.69 (CI: 0.63-0.75) |
| 5-year | 0.92 (CI: 0.88-0.97) | 0.92 (CI: 0.88-0.96) | 0.88 (CI: 0.84-0.93) | **0.93 (CI: 0.89-0.97)** | 0.90 (CI: 0.86-0.95) | 0.87 (CI: 0.82-0.93) | 0.68 (CI: 0.60-0.77) |
| Average | **0.86 (CI: 0.83-0.89)** | 0.81 (CI: 0.78-0.85) | 0.80 (CI: 0.76-0.84) | **0.87 (CI: 0.84-0.90)** | 0.81 (CI: 0.77-0.85) | 0.75 (CI: 0.71-0.79) | 0.71 (CI: 0.66-0.76) |

**Table S3O**: AUC values with 95% Confidence Intervals (CI) rounded at 2 digits for XGBoost, GRU, Logistic Regression, LightGBM, LSTM, Naïve Bayes models and the clinical model in predicting clinical outcomes of the men population at different visit intervals over a 5-year term using the cumulative approach.

|  | **Prediction at 5 years term** | | | | | | |
| --- | --- | --- | --- | --- | --- | --- | --- |
| **Visit** | **XGboost** | **GRU** | **LogisticRegression** | **LightGBM** | **LSTM** | **Naïve Bayes** | **Clinical model** |
| Baseline | **0.80 (CI: 0.78-0.81)** | 0.73 (CI: 0.71-0.74) | 0.71 (CI: 0.70-0.73) | 0.78 (CI: 0.77-0.80) | 0.72 (CI: 0.71-0.74) | 0.63 (CI: 0.61-0.65) | 0.74 (CI: 0.72-0.75) |
| 1-year | **0.85 (CI: 0.83-0.86)** | 0.82 (CI: 0.80-0.83) | 0.79 (CI: 0.78-0.81) | 0.84 (CI: 0.83-0.86) | 0.81 (CI: 0.79-0.83) | 0.71 (CI: 0.69-0.72) | 0.76 (CI: 0.75-0.77) |
| 2-year | **0.84 (CI: 0.83-0.86)** | 0.82 (CI: 0.80-0.83) | 0.83 (CI: 0.81-0.84) | **0.84 (CI: 0.83-0.86)** | 0.81 (CI: 0.79-0.82) | 0.71 (CI: 0.69-0.73) | 0.74 (CI: 0.72-0.75) |
| 3-year | 0.81 (CI: 0.79-0.83) | **0.82 (CI: 0.80-0.84)** | 0.77 (CI: 0.75-0.79) | **0.82 (CI: 0.80-0.84)** | 0.81 (CI: 0.79-0.83) | 0.68 (CI: 0.66-0.70) | 0.74 (CI: 0.72-0.76) |
| 4-year | 0.74 (CI: 0.72-0.77) | **0.77 (CI: 0.74-0.79)** | **0.77 (CI: 0.74-0.79)** | 0.74 (CI: 0.72-0.77) | **0.77 (CI: 0.74-0.79)** | 0.61 (CI: 0.58-0.63) | 0.72 (CI: 0.70-0.75) |
| 5-year | 0.77 (CI: 0.74-0.80) | 0.76 (CI: 0.72-0.79) | **0.80 (CI: 0.76-0.83)** | 0.76 (CI: 0.73-0.80) | 0.77 (CI: 0.73-0.81) | 0.50 (CI: 0.47-0.53) | 0.64 (CI: 0.59-0.68) |
| Average | **0.80 (CI: 0.78-0.82)** | 0.79 (CI: 0.76-0.80) | 0.78 (CI: 0.76-0.80) | **0.80 (CI: 0.78-0.82)** | 0.78 (CI: 0.76-0.80) | 0.64 (CI: 0.62-0.66) | 0.72 (CI: 0.70-0.74) |

**Table S3P**: AUC values with 95% Confidence Intervals (CI) rounded at 2 digits for XGBoost, GRU, Logistic Regression, LightGBM, LSTM, Naïve Bayes models and the clinical model in predicting clinical outcomes of the men population at different visit intervals over a 2-year term using the cumulative approach.

|  | **Prediction at 2 years term** | | | | | | |
| --- | --- | --- | --- | --- | --- | --- | --- |
| **Visit** | **XGboost** | **GRU** | **LogisticRegression** | **LightGBM** | **LSTM** | **Naïve Bayes** | **Clinical model** |
| Baseline | 0.69 (CI: 0.67-0.70) | 0.66 (CI: 0.64-0.68) | 0.62 (CI: 0.60-0.64) | **0.69 (CI: 0.67-0.71)** | 0.66 (CI: 0.64-0.68) | 0.50 (CI: 0.48-0.51) | 0.64 (CI: 0.62-0.66) |
| 1-year | **0.86 (CI: 0.85-0.87)** | 0.81 (CI: 0.80-0.83) | 0.84 (CI: 0.82-0.85) | 0.86 (CI: 0.84-0.87) | 0.81 (CI: 0.79-0.82) | 0.66 (CI: 0.64-0.68) | 0.71 (CI: 0.70-0.73) |
| 2-year | **0.88 (CI: 0.87-0.90)** | 0.85 (CI: 0.84-0.87) | 0.87 (CI: 0.86-0.88) | 0.88 (CI: 0.86-0.89) | 0.84 (CI: 0.82-0.85) | 0.69 (CI: 0.67-0.71) | 0.70 (CI: 0.68-0.72) |
| 3-year | 0.89 (CI: 0.88-0.91) | 0.87 (CI: 0.85-0.88) | 0.87 (CI: 0.86-0.89) | **0.90 (CI: 0.88-0.91)** | 0.86 (CI: 0.84-0.88) | 0.61 (CI: 0.59-0.64) | 0.75 (CI: 0.73-0.77) |
| 4-year | **0.90 (CI: 0.88-0.91)** | 0.86 (CI: 0.84-0.88) | 0.84 (CI: 0.82-0.86) | 0.89 (CI: 0.87-0.91) | 0.86 (CI: 0.84-0.88) | 0.55 (CI: 0.52-0.58) | 0.76 (CI: 0.73-0.78) |
| 5-year | 0.76 (CI: 0.73-0.79) | 0.79 (CI: 0.76-0.82) | **0.80 (CI: 0.77-0.83)** | 0.77 (CI: 0.74-0.80) | 0.79 (CI: 0.76-0.82) | 0.47 (CI: 0.43-0.50) | 0.63 (CI: 0.59-0.67) |
| Average | **0.83 (CI: 0.81-0.85)** | 0.81 (CI: 0.79-0.83) | 0.81 (CI: 0.79-0.83) | **0.83 (CI: 0.81-0.85)** | 0.80 (CI: 0.78-0.82) | 0.58 (CI: 0.56-0.60) | 0.70 (CI: 0.68-0.72) |

**Table S3Q**: AUC values with 95% Confidence Intervals (CI) rounded at 2 digits for XGBoost, GRU, Logistic Regression, LightGBM, LSTM, Naïve Bayes models and the clinical model in predicting clinical outcomes of the women population at different visit intervals over a 5-year term using the cumulative approach.

|  | **Prediction at 5 years term** | | | | | | |
| --- | --- | --- | --- | --- | --- | --- | --- |
| **Visit** | **XGboost** | **GRU** | **LogisticRegression** | **LightGBM** | **LSTM** | **Naïve Bayes** | **Clinical model** |
| Baseline | **0.80 (CI: 0.77-0.82)** | 0.75 (CI: 0.71-0.78) | 0.74 (CI: 0.71-0.77) | 0.79 (CI: 0.76-0.81) | 0.74 (CI: 0.71-0.77) | 0.63 (CI: 0.60-0.66) | 0.60 (CI: 0.57-0.64) |
| 1-year | **0.84 (CI: 0.82-0.87)** | 0.83 (CI: 0.80-0.86) | 0.79 (CI: 0.77-0.82) | 0.83 (CI: 0.81-0.86) | 0.81 (CI: 0.79-0.84) | 0.72 (CI: 0.69-0.75) | 0.67 (CI: 0.63-0.70) |
| 2-year | 0.83 (CI: 0.80-0.86) | **0.84 (CI: 0.81-0.87)** | 0.76 (CI: 0.72-0.79) | 0.82 (CI: 0.79-0.85) | 0.83 (CI: 0.81-0.86) | 0.67 (CI: 0.64-0.71) | 0.71 (CI: 0.68-0.76) |
| 3-year | 0.77 (CI: 0.73-0.81) | **0.83 (CI: 0.80-0.87)** | 0.75 (CI: 0.71-0.78) | 0.78 (CI: 0.74-0.82) | 0.82 (CI: 0.79-0.85) | 0.61 (CI: 0.57-0.65) | 0.68 (CI: 0.63-0.73) |
| 4-year | 0.77 (CI: 0.72-0.83) | **0.82 (CI: 0.78-0.87)** | 0.75 (CI: 0.70-0.81) | 0.78 (CI: 0.73-0.83) | 0.81 (CI: 0.76-0.86) | 0.58 (CI: 0.53-0.62) | 0.73 (CI: 0.68-0.78) |
| 5-year | 0.89 (CI: 0.84-0.94) | 0.91 (CI: 0.87-0.95) | 0.88 (CI: 0.83-0.93) | 0.88 (CI: 0.84-0.92) | **0.91 (CI: 0.87-0.96)** | 0.57 (CI: 0.53-0.61) | 0.83 (CI: 0.78-0.89) |
| Average | 0.82 (CI: 0.78-0.86) | **0.83 (CI: 0.80-0.87)** | 0.78 (CI: 0.74-0.82) | 0.81 (CI: 0.78-0.85) | 0.82 (CI: 0.79-0.86) | 0.63 (CI: 0.59-0.67) | 0.70 (CI: 0.66-0.75) |

**Table S3R**: AUC values with 95% Confidence Intervals (CI) rounded at 2 digits for XGBoost, GRU, Logistic Regression, LightGBM, LSTM, Naïve Bayes models and the clinical model in predicting clinical outcomes of the women population at different visit intervals over a 2-year term using the cumulative approach.

|  | **Prediction at 2 years term** | | | | | | |
| --- | --- | --- | --- | --- | --- | --- | --- |
| **Visit** | **XGboost** | **GRU** | **LogisticRegression** | **LightGBM** | **LSTM** | **Naïve Bayes** | **Clinical model** |
| Baseline | 0.70 (CI: 0.66-0.73) | 0.65 (CI: 0.61-0.69) | 0.67 (CI: 0.64-0.71) | **0.70 (CI: 0.67-0.73)** | 0.64 (CI: 0.60-0.68) | 0.48 (CI: 0.44-0.51) | 0.57 (CI: 0.54-0.61) |
| 1-year | **0.83 (CI: 0.81-0.86)** | 0.79 (CI: 0.76-0.82) | 0.76 (CI: 0.73-0.79) | 0.83 (CI: 0.81-0.85) | 0.78 (CI: 0.75-0.81) | 0.70 (CI: 0.67-0.74) | 0.72 (CI: 0.69-0.75) |
| 2-year | **0.89 (CI: 0.87-0.91)** | 0.84 (CI: 0.80-0.87) | 0.80 (CI: 0.77-0.83) | 0.88 (CI: 0.86-0.90) | 0.83 (CI: 0.79-0.86) | 0.60 (CI: 0.56-0.65) | 0.75 (CI: 0.71-0.78) |
| 3-year | 0.88 (CI: 0.85-0.90) | 0.81 (CI: 0.78-0.85) | 0.83 (CI: 0.80-0.86) | **0.90 (CI: 0.87-0.92)** | 0.83 (CI: 0.80-0.87) | 0.60 (CI: 0.55-0.65) | 0.73 (CI: 0.70-0.77) |
| 4-year | 0.90 (CI: 0.86-0.94) | 0.86 (CI: 0.82-0.90) | 0.78 (CI: 0.73-0.83) | **0.90 (CI: 0.87-0.95)** | 0.87 (CI: 0.84-0.91) | 0.58 (CI: 0.52-0.63) | 0.81 (CI: 0.76-0.86) |
| 5-year | 0.81 (CI: 0.76-0.87) | **0.92 (CI: 0.88-0.96)** | 0.85 (CI: 0.80-0.91) | 0.82 (CI: 0.77-0.87) | 0.90 (CI: 0.86-0.95) | 0.54 (CI: 0.49-0.59) | 0.79 (CI: 0.74-0.85) |
| Average | **0.84 (CI: 0.80-0.87)** | 0.81 (CI: 0.78-0.85) | 0.78 (CI: 0.75-0.82) | **0.84 (CI: 0.81-0.87)** | 0.81 (CI: 0.77-0.85) | 0.58 (CI: 0.54-0.63) | 0.73 (CI: 0.69-0.77) |

**Table S3S**: AUC values with 95% Confidence Intervals (CI) rounded at 2 digits for XGBoost, GRU, Logistic Regression, LightGBM, LSTM, Naïve Bayes models and the clinical model in predicting clinical outcomes of the bicuspid population at different visit intervals over a 5-year term using the non-cumulative approach.

|  | **Prediction at 5 years term** | | | | | | |
| --- | --- | --- | --- | --- | --- | --- | --- |
| **Visit** | **XGboost** | **GRU** | **LogisticRegression** | **LightGBM** | **LSTM** | **Naïve Bayes** | **Clinical model** |
| Baseline | **0.80 (CI: 0.79-0.82)** | 0.73 (CI: 0.71-0.75) | 0.72 (CI: 0.71-0.74) | **0.80 (CI: 0.79-0.82)** | 0.72 (CI: 0.71-0.74) | 0.71 (CI: 0.70-0.73) | 0.68 (CI: 0.67-0.70) |
| 1-year | 0.84 (CI: 0.83-0.86) | 0.82 (CI: 0.81-0.84) | 0.79 (CI: 0.78-0.81) | **0.85 (CI: 0.83-0.86)** | 0.82 (CI: 0.80-0.83) | 0.77 (CI: 0.75-0.79) | 0.75 (CI: 0.73-0.76) |
| 2-year | 0.81 (CI: 0.80-0.83) | 0.79 (CI: 0.78-0.81) | 0.78 (CI: 0.76-0.79) | **0.83 (CI: 0.81-0.84)** | 0.78 (CI: 0.77-0.80) | 0.74 (CI: 0.72-0.77) | 0.69 (CI: 0.67-0.72) |
| 3-year | **0.85 (CI: 0.83-0.87)** | 0.80 (CI: 0.78-0.82) | 0.77 (CI: 0.75-0.80) | **0.85 (CI: 0.83-0.87)** | 0.79 (CI: 0.77-0.81) | 0.75 (CI: 0.72-0.77) | 0.64 (CI: 0.61-0.67) |
| 4-year | 0.78 (CI: 0.76-0.81) | 0.74 (CI: 0.72-0.77) | 0.73 (CI: 0.70-0.76) | **0.79 (CI: 0.76-0.81)** | 0.74 (CI: 0.71-0.77) | 0.70 (CI: 0.67-0.72) | 0.68 (CI: 0.65-0.71) |
| 5-year | 0.81 (CI: 0.77-0.84) | 0.81 (CI: 0.77-0.85) | 0.81 (CI: 0.77-0.85) | **0.82 (CI: 0.79-0.86)** | 0.82 (CI: 0.78-0.85) | 0.68 (CI: 0.64-0.72) | 0.71 (CI: 0.67-0.75) |
| Average | **0.82 (CI: 0.80-0.84)** | 0.78 (CI: 0.76-0.81) | 0.77 (CI: 0.75-0.79) | **0.82 (CI: 0.80-0.84)** | 0.78 (CI: 0.76-0.80) | 0.73 (CI: 0.70-0.75) | 0.69 (CI: 0.67-0.72) |

**Table S3T**: AUC values with 95% Confidence Intervals (CI) rounded at 2 digits for XGBoost, GRU, Logistic Regression, LightGBM, LSTM, Naïve Bayes models and the clinical model in predicting clinical outcomes of the bicuspid population at different visit intervals over a 2-year term using the non-cumulative approach.

|  | **Prediction at 2 years term** | | | | | | |
| --- | --- | --- | --- | --- | --- | --- | --- |
| **Visit** | **XGboost** | **GRU** | **LogisticRegression** | **LightGBM** | **LSTM** | **Naïve Bayes** | **Clinical model** |
| Baseline | 0.76 (CI: 0.75-0.78) | 0.69 (CI: 0.67-0.71) | 0.68 (CI: 0.66-0.70) | **0.77 (CI: 0.75-0.78)** | 0.69 (CI: 0.67-0.71) | 0.64 (CI: 0.62-0.66) | 0.65 (CI: 0.63-0.67) |
| 1-year | 0.85 (CI: 0.83-0.86) | 0.82 (CI: 0.81-0.84) | 0.81 (CI: 0.79-0.82) | **0.86 (CI: 0.85-0.87)** | 0.82 (CI: 0.80-0.83) | 0.77 (CI: 0.76-0.79) | 0.75 (CI: 0.73-0.77) |
| 2-year | **0.88 (CI: 0.86-0.89)** | 0.86 (CI: 0.84-0.87) | 0.81 (CI: 0.80-0.83) | 0.87 (CI: 0.86-0.89) | 0.85 (CI: 0.84-0.87) | 0.77 (CI: 0.75-0.79) | 0.73 (CI: 0.71-0.74) |
| 3-year | 0.91 (CI: 0.89-0.92) | 0.88 (CI: 0.87-0.90) | 0.88 (CI: 0.86-0.90) | **0.91 (CI: 0.90-0.92)** | 0.88 (CI: 0.86-0.90) | 0.77 (CI: 0.74-0.79) | 0.75 (CI: 0.72-0.77) |
| 4-year | 0.89 (CI: 0.88-0.91) | 0.87 (CI: 0.85-0.89) | 0.80 (CI: 0.78-0.83) | **0.90 (CI: 0.88-0.92)** | 0.87 (CI: 0.85-0.89) | 0.74 (CI: 0.71-0.76) | 0.77 (CI: 0.73-0.80) |
| 5-year | **0.85 (CI: 0.83-0.88)** | 0.82 (CI: 0.79-0.85) | 0.76 (CI: 0.72-0.79) | 0.84 (CI: 0.81-0.87) | 0.83 (CI: 0.80-0.86) | 0.70 (CI: 0.66-0.74) | 0.73 (CI: 0.68-0.79) |
| Average | 0.86 (CI: 0.84-0.87) | 0.82 (CI: 0.81-0.84) | 0.79 (CI: 0.77-0.81) | **0.86 (CI: 0.84-0.88)** | 0.82 (CI: 0.80-0.84) | 0.73 (CI: 0.71-0.76) | 0.73 (CI: 0.70-0.76) |

**Table S3U**: AUC values with 95% Confidence Intervals (CI) rounded at 2 digits for XGBoost, GRU, Logistic Regression, LightGBM, LSTM, Naïve Bayes models and the clinical model in predicting clinical outcomes of the tricuspid population at different visit intervals over a 5-year term using the non-cumulative approach.

|  | **Prediction at 5 years term** | | | | | | |
| --- | --- | --- | --- | --- | --- | --- | --- |
| **Visit** | **XGboost** | **GRU** | **LogisticRegression** | **LightGBM** | **LSTM** | **Naïve Bayes** | **Clinical model** |
| Baseline | 0.79 (CI: 0.77-0.82) | 0.77 (CI: 0.74-0.80) | 0.77 (CI: 0.73-0.80) | 0.80 (CI: 0.78-0.83) | 0.76 (CI: 0.73-0.79) | **0.82 (CI: 0.80-0.85)** | 0.74 (CI: 0.71-0.78) |
| 1-year | 0.84 (CI: 0.82-0.86) | 0.84 (CI: 0.82-0.87) | 0.84 (CI: 0.81-0.86) | 0.84 (CI: 0.81-0.86) | 0.84 (CI: 0.81-0.86) | **0.88 (CI: 0.86-0.90)** | 0.65 (CI: 0.62-0.69) |
| 2-year | 0.87 (CI: 0.84-0.90) | **0.89 (CI: 0.87-0.92)** | 0.89 (CI: 0.87-0.91) | 0.87 (CI: 0.84-0.90) | 0.89 (CI: 0.86-0.92) | 0.89 (CI: 0.86-0.91) | 0.73 (CI: 0.68-0.77) |
| 3-year | 0.86 (CI: 0.83-0.90) | **0.91 (CI: 0.88-0.94)** | 0.85 (CI: 0.81-0.89) | 0.88 (CI: 0.85-0.91) | 0.89 (CI: 0.86-0.92) | 0.89 (CI: 0.86-0.92) | 0.65 (CI: 0.59-0.72) |
| 4-year | 0.84 (CI: 0.79-0.90) | **0.85 (CI: 0.80-0.90)** | 0.77 (CI: 0.71-0.83) | 0.82 (CI: 0.77-0.88) | 0.83 (CI: 0.78-0.89) | **0.85 (CI: 0.80-0.90)** | 0.67 (CI: 0.60-0.74) |
| 5-year | 0.86 (CI: 0.80-0.93) | 0.87 (CI: 0.81-0.94) | 0.84 (CI: 0.76-0.92) | 0.84 (CI: 0.78-0.92) | 0.87 (CI: 0.81-0.94) | **0.88 (CI: 0.82-0.95)** | 0.69 (CI: 0.61-0.78) |
| Average | 0.84 (CI: 0.81-0.89) | 0.86 (CI: 0.82-0.90) | 0.83 (CI: 0.78-0.87) | 0.84 (CI: 0.81-0.88) | 0.85 (CI: 0.81-0.89) | **0.87 (CI: 0.83-0.91)** | 0.69 (CI: 0.64-0.75) |

**Table S3V**: AUC values with 95% Confidence Intervals (CI) rounded at 2 digits for XGBoost, GRU, Logistic Regression, LightGBM, LSTM, Naïve Bayes models and the clinical model in predicting clinical outcomes of the tricuspid population at different visit intervals over a 2-year term using the non-cumulative approach.

|  | **Prediction at 2 years term** | | | | | | |
| --- | --- | --- | --- | --- | --- | --- | --- |
| **Visit** | **XGboost** | **GRU** | **LogisticRegression** | **LightGBM** | **LSTM** | **Naïve Bayes** | **Clinical model** |
| Baseline | 0.64 (CI: 0.60-0.67) | 0.58 (CI: 0.55-0.62) | 0.60 (CI: 0.56-0.63) | **0.64 (CI: 0.61-0.68)** | 0.55 (CI: 0.51-0.59) | 0.54 (CI: 0.50-0.58) | 0.57 (CI: 0.53-0.61) |
| 1-year | 0.75 (CI: 0.72-0.79) | 0.75 (CI: 0.72-0.79) | 0.75 (CI: 0.72-0.79) | **0.76 (CI: 0.73-0.80)** | 0.73 (CI: 0.69-0.76) | 0.70 (CI: 0.66-0.73) | 0.58 (CI: 0.54-0.61) |
| 2-year | 0.82 (CI: 0.79-0.85) | **0.83 (CI: 0.80-0.87)** | 0.81 (CI: 0.78-0.85) | 0.83 (CI: 0.80-0.86) | 0.83 (CI: 0.80-0.86) | 0.77 (CI: 0.73-0.80) | 0.75 (CI: 0.71-0.80) |
| 3-year | 0.76 (CI: 0.71-0.81) | **0.79 (CI: 0.74-0.83)** | 0.70 (CI: 0.66-0.75) | 0.78 (CI: 0.73-0.82) | 0.76 (CI: 0.72-0.81) | 0.71 (CI: 0.66-0.76) | 0.75 (CI: 0.70-0.81) |
| 4-year | 0.84 (CI: 0.78-0.90) | **0.85 (CI: 0.80-0.90)** | 0.73 (CI: 0.66-0.80) | **0.85 (CI: 0.80-0.90)** | 0.84 (CI: 0.79-0.90) | 0.77 (CI: 0.71-0.84) | 0.74 (CI: 0.68-0.81) |
| 5-year | 0.83 (CI: 0.75-0.92) | **0.88 (CI: 0.82-0.96)** | 0.80 (CI: 0.72-0.89) | 0.83 (CI: 0.76-0.92) | 0.88 (CI: 0.81-0.95) | 0.88 (CI: 0.81-0.95) | 0.69 (CI: 0.59-0.79) |
| Average | 0.77 (CI: 0.73-0.82) | **0.78 (CI: 0.74-0.83)** | 0.73 (CI: 0.68-0.79) | **0.78 (CI: 0.74-0.83)** | 0.77 (CI: 0.72-0.81) | 0.73 (CI: 0.68-0.78) | 0.68 (CI: 0.63-0.74) |

**Table S3W**: AUC values with 95% Confidence Intervals (CI) rounded at 2 digits for XGBoost, GRU, Logistic Regression, LightGBM, LSTM, Naïve Bayes models and the clinical model in predicting clinical outcomes of the bicuspid population at different visit intervals over a 5-year term using the cumulative approach.

|  | **Prediction at 5 years term** | | | | | | |
| --- | --- | --- | --- | --- | --- | --- | --- |
| **Visit** | **XGboost** | **GRU** | **LogisticRegression** | **LightGBM** | **LSTM** | **Naïve Bayes** | **Clinical model** |
| Baseline | **0.80 (CI: 0.78-0.81)** | 0.73 (CI: 0.71-0.75) | 0.71 (CI: 0.70-0.73) | 0.78 (CI: 0.76-0.79) | 0.72 (CI: 0.71-0.74) | 0.61 (CI: 0.59-0.63) | 0.68 (CI: 0.66-0.69) |
| 1-year | **0.86 (CI: 0.85-0.88)** | 0.82 (CI: 0.81-0.84) | 0.80 (CI: 0.78-0.81) | 0.85 (CI: 0.84-0.87) | 0.82 (CI: 0.80-0.83) | 0.69 (CI: 0.67-0.71) | 0.74 (CI: 0.72-0.75) |
| 2-year | **0.82 (CI: 0.81-0.84)** | 0.79 (CI: 0.78-0.81) | 0.78 (CI: 0.76-0.80) | 0.82 (CI: 0.80-0.84) | 0.78 (CI: 0.77-0.80) | 0.66 (CI: 0.64-0.68) | 0.71 (CI: 0.69-0.73) |
| 3-year | 0.82 (CI: 0.80-0.84) | 0.80 (CI: 0.78-0.82) | 0.73 (CI: 0.71-0.76) | **0.82 (CI: 0.81-0.84)** | 0.79 (CI: 0.77-0.81) | 0.62 (CI: 0.59-0.64) | 0.71 (CI: 0.69-0.73) |
| 4-year | 0.75 (CI: 0.72-0.77) | 0.74 (CI: 0.72-0.77) | 0.72 (CI: 0.69-0.75) | **0.76 (CI: 0.73-0.78)** | 0.74 (CI: 0.71-0.77) | 0.55 (CI: 0.52-0.57) | 0.68 (CI: 0.65-0.71) |
| 5-year | 0.80 (CI: 0.77-0.83) | 0.81 (CI: 0.77-0.85) | 0.81 (CI: 0.78-0.84) | 0.80 (CI: 0.77-0.84) | **0.82 (CI: 0.78-0.85)** | 0.46 (CI: 0.42-0.49) | 0.66 (CI: 0.62-0.70) |
| Average | **0.81 (CI: 0.79-0.83)** | 0.78 (CI: 0.76-0.81) | 0.76 (CI: 0.74-0.78) | **0.81 (CI: 0.79-0.83)** | 0.78 (CI: 0.76-0.80) | 0.60 (CI: 0.57-0.62) | 0.70 (CI: 0.67-0.72) |

**Table S3X**: AUC values with 95% Confidence Intervals (CI) rounded at 2 digits for XGBoost, GRU, Logistic Regression, LightGBM, LSTM, Naïve Bayes models and the clinical model in predicting clinical outcomes of the bicuspid population at different visit intervals over a 2-year term using the cumulative approach.

|  | **Prediction at 2 years term** | | | | | | |
| --- | --- | --- | --- | --- | --- | --- | --- |
| **Visit** | **XGboost** | **GRU** | **LogisticRegression** | **LightGBM** | **LSTM** | **Naïve Bayes** | **Clinical model** |
| Baseline | 0.69 (CI: 0.68-0.71) | 0.69 (CI: 0.67-0.71) | 0.65 (CI: 0.63-0.67) | **0.70 (CI: 0.68-0.72)** | 0.69 (CI: 0.67-0.71) | 0.52 (CI: 0.50-0.53) | 0.62 (CI: 0.60-0.64) |
| 1-year | 0.85 (CI: 0.84-0.87) | 0.82 (CI: 0.81-0.84) | 0.82 (CI: 0.80-0.83) | **0.86 (CI: 0.84-0.87)** | 0.82 (CI: 0.80-0.83) | 0.69 (CI: 0.67-0.71) | 0.72 (CI: 0.70-0.74) |
| 2-year | **0.89 (CI: 0.88-0.91)** | 0.86 (CI: 0.84-0.87) | 0.87 (CI: 0.85-0.88) | 0.89 (CI: 0.87-0.90) | 0.85 (CI: 0.84-0.87) | 0.68 (CI: 0.66-0.70) | 0.73 (CI: 0.71-0.75) |
| 3-year | 0.90 (CI: 0.89-0.92) | 0.88 (CI: 0.87-0.90) | 0.88 (CI: 0.86-0.89) | **0.91 (CI: 0.90-0.93)** | 0.88 (CI: 0.86-0.90) | 0.64 (CI: 0.61-0.66) | 0.77 (CI: 0.75-0.79) |
| 4-year | **0.92 (CI: 0.90-0.93)** | 0.87 (CI: 0.85-0.89) | 0.82 (CI: 0.80-0.85) | 0.90 (CI: 0.89-0.92) | 0.87 (CI: 0.85-0.89) | 0.54 (CI: 0.51-0.57) | 0.75 (CI: 0.72-0.78) |
| 5-year | 0.79 (CI: 0.76-0.82) | 0.82 (CI: 0.79-0.85) | 0.79 (CI: 0.76-0.82) | 0.79 (CI: 0.76-0.83) | **0.83 (CI: 0.80-0.86)** | 0.48 (CI: 0.44-0.51) | 0.65 (CI: 0.60-0.69) |
| Average | **0.84 (CI: 0.83-0.86)** | 0.82 (CI: 0.81-0.84) | 0.81 (CI: 0.78-0.82) | 0.84 (CI: 0.82-0.86) | 0.82 (CI: 0.80-0.84) | 0.59 (CI: 0.57-0.61) | 0.71 (CI: 0.68-0.73) |

**Table S3Y**: AUC values with 95% Confidence Intervals (CI) rounded at 2 digits for XGBoost, GRU, Logistic Regression, LightGBM, LSTM, Naïve Bayes models and the clinical model in predicting clinical outcomes of the tricuspid population at different visit intervals over a 5-year term using the cumulative approach.

|  | **Prediction at 5 years term** | | | | | | |
| --- | --- | --- | --- | --- | --- | --- | --- |
| **Visit** | **XGboost** | **GRU** | **LogisticRegression** | **LightGBM** | **LSTM** | **Naïve Bayes** | **Clinical model** |
| Baseline | **0.82 (CI: 0.80-0.85)** | 0.77 (CI: 0.74-0.80) | 0.78 (CI: 0.76-0.81) | 0.81 (CI: 0.79-0.84) | 0.76 (CI: 0.73-0.79) | 0.75 (CI: 0.72-0.79) | 0.73 (CI: 0.70-0.77) |
| 1-year | 0.84 (CI: 0.81-0.87) | **0.84 (CI: 0.82-0.87)** | 0.79 (CI: 0.76-0.82) | 0.84 (CI: 0.81-0.86) | 0.84 (CI: 0.81-0.86) | 0.80 (CI: 0.77-0.83) | 0.69 (CI: 0.66-0.73) |
| 2-year | **0.90 (CI: 0.88-0.93)** | 0.89 (CI: 0.87-0.92) | 0.88 (CI: 0.86-0.91) | **0.90 (CI: 0.88-0.93)** | 0.89 (CI: 0.86-0.92) | 0.85 (CI: 0.83-0.88) | 0.78 (CI: 0.75-0.82) |
| 3-year | 0.82 (CI: 0.78-0.87) | **0.91 (CI: 0.88-0.94)** | 0.87 (CI: 0.84-0.91) | 0.81 (CI: 0.77-0.86) | 0.89 (CI: 0.86-0.92) | 0.75 (CI: 0.72-0.79) | 0.77 (CI: 0.72-0.81) |
| 4-year | 0.74 (CI: 0.67-0.82) | **0.85 (CI: 0.80-0.90)** | 0.83 (CI: 0.78-0.89) | 0.74 (CI: 0.67-0.81) | 0.83 (CI: 0.78-0.89) | 0.70 (CI: 0.65-0.75) | 0.74 (CI: 0.67-0.80) |
| 5-year | 0.80 (CI: 0.73-0.88) | 0.87 (CI: 0.81-0.94) | **0.88 (CI: 0.82-0.95)** | 0.77 (CI: 0.69-0.86) | 0.87 (CI: 0.81-0.94) | 0.69 (CI: 0.61-0.76) | 0.74 (CI: 0.66-0.82) |
| Average | 0.82 (CI: 0.78-0.87) | **0.86 (CI: 0.82-0.90)** | 0.84 (CI: 0.80-0.88) | 0.81 (CI: 0.77-0.86) | 0.85 (CI: 0.81-0.89) | 0.76 (CI: 0.72-0.80) | 0.74 (CI: 0.69-0.79) |

**Table S3Z**: AUC values with 95% Confidence Intervals (CI) rounded at 2 digits for XGBoost, GRU, Logistic Regression, LightGBM, LSTM, Naïve Bayes models and the clinical model in predicting clinical outcomes of the tricuspid population at different visit intervals over a 2-year term using the cumulative approach.

|  | **Prediction at 2 years term** | | | | | | |
| --- | --- | --- | --- | --- | --- | --- | --- |
| **Visit** | **XGboost** | **GRU** | **LogisticRegression** | **LightGBM** | **LSTM** | **Naïve Bayes** | **Clinical model** |
| Baseline | 0.66 (CI: 0.63-0.69) | 0.58 (CI: 0.55-0.62) | 0.60 (CI: 0.56-0.64) | **0.67 (CI: 0.64-0.70)** | 0.55 (CI: 0.51-0.59) | 0.47 (CI: 0.44-0.51) | 0.58 (CI: 0.55-0.62) |
| 1-year | **0.81 (CI: 0.78-0.83)** | 0.75 (CI: 0.72-0.79) | 0.80 (CI: 0.76-0.83) | 0.79 (CI: 0.76-0.82) | 0.73 (CI: 0.69-0.76) | 0.64 (CI: 0.60-0.68) | 0.58 (CI: 0.55-0.62) |
| 2-year | **0.85 (CI: 0.82-0.87)** | 0.83 (CI: 0.80-0.87) | 0.83 (CI: 0.80-0.86) | 0.84 (CI: 0.81-0.87) | 0.83 (CI: 0.80-0.86) | 0.69 (CI: 0.65-0.74) | 0.68 (CI: 0.65-0.72) |
| 3-year | 0.78 (CI: 0.73-0.83) | 0.79 (CI: 0.74-0.83) | **0.83 (CI: 0.80-0.87)** | 0.80 (CI: 0.76-0.85) | 0.76 (CI: 0.72-0.81) | 0.56 (CI: 0.50-0.61) | 0.70 (CI: 0.65-0.75) |
| 4-year | **0.89 (CI: 0.85-0.94)** | 0.85 (CI: 0.80-0.90) | 0.82 (CI: 0.77-0.88) | 0.88 (CI: 0.84-0.93) | 0.84 (CI: 0.79-0.90) | 0.71 (CI: 0.65-0.77) | **0.89 (CI: 0.85-0.94)** |
| 5-year | 0.71 (CI: 0.62-0.80) | **0.88 (CI: 0.82-0.96)** | 0.86 (CI: 0.80-0.94) | 0.75 (CI: 0.66-0.84) | 0.88 (CI: 0.81-0.95) | 0.59 (CI: 0.51-0.68) | 0.76 (CI: 0.67-0.85) |
| Average | 0.78 (CI: 0.74-0.83) | 0.78 (CI: 0.74-0.83) | **0.79 (CI: 0.75-0.84)** | **0.79 (CI: 0.75-0.84)** | 0.77 (CI: 0.72-0.81) | 0.61 (CI: 0.56-0.67) | 0.70 (CI: 0.65-0.75) |

**MATTHEWS CORRELATION COEFFICIENT (MCC) RESULTS**

**Table S4alpha**: MCC values with 95% Confidence Intervals (CI) rounded at 2 digits for XGBoost, GRU, Logistic Regression, LightGBM, LSTM, Naïve Bayes models and the clinical model in predicting clinical outcomes at different visit intervals over a 5-year term using the non-cumulative approach. This table represent the MCC values of the AUC values presented in table 2A in the main manuscript and has been called alpha to maintain the A-Z hierarchy of the of other tables S4.

|  | **Prediction at 5 years term** | | | | | | |
| --- | --- | --- | --- | --- | --- | --- | --- |
| **Visit** | **XGboost** | **GRU** | **LogisticRegression** | **LightGBM** | **LSTM** | **Naïve Bayes** | **Clinical model** |
| Baseline | **0.48 (CI: 0.46-0.50)** | 0.33 (CI: 0.31-0.35) | 0.34 (CI: 0.32-0.37) | 0.47 (CI: 0.45-0.49) | 0.31 (CI: 0.28-0.34) | 0.03 (CI: 0.01-0.06) | 0.29 (CI: 0.27-0.32) |
| 1-year | 0.53 (CI: 0.51-0.55) | 0.52 (CI: 0.49-0.54) | 0.45 (CI: 0.42-0.47) | **0.54 (CI: 0.52-0.56)** | 0.49 (CI: 0.47-0.52) | 0.17 (CI: 0.15-0.20) | 0.29 (CI: 0.27-0.32) |
| 2-year | **0.54 (CI: 0.52-0.56)** | 0.50 (CI: 0.47-0.52) | 0.46 (CI: 0.43-0.49) | 0.53 (CI: 0.51-0.55) | 0.49 (CI: 0.46-0.51) | 0.07 (CI: 0.04-0.10) | 0.39 (CI: 0.37-0.42) |
| 3-year | 0.55 (CI: 0.52-0.58) | 0.49 (CI: 0.46-0.52) | 0.44 (CI: 0.41-0.47) | **0.56 (CI: 0.52-0.59)** | 0.49 (CI: 0.46-0.52) | 0.10 (CI: 0.06-0.14) | 0.23 (CI: 0.20-0.25) |
| 4-year | **0.44 (CI: 0.41-0.48)** | 0.42 (CI: 0.38-0.46) | 0.39 (CI: 0.35-0.44) | 0.43 (CI: 0.40-0.47) | 0.41 (CI: 0.37-0.46) | 0.12 (CI: 0.08-0.15) | 0.21 (CI: 0.18-0.24) |
| 5-year | 0.44 (CI: 0.39-0.49) | **0.50 (CI: 0.46-0.55)** | 0.43 (CI: 0.39-0.48) | 0.46 (CI: 0.41-0.51) | 0.48 (CI: 0.43-0.53) | 0.10 (CI: 0.06-0.14) | 0.12 (CI: 0.09-0.15) |
| Average | 0.50 (CI: 0.47-0.53) | 0.46 (CI: 0.43-0.49) | 0.42 (CI: 0.39-0.45) | **0.50 (CI: 0.47-0.53)** | 0.45 (CI: 0.41-0.48) | 0.10 (CI: 0.07-0.13) | 0.26 (CI: 0.23-0.28) |

**Table S4beta**: MCC values with 95% Confidence Intervals (CI) rounded at 2 digits for XGBoost, GRU, Logistic Regression, LightGBM, LSTM, Naïve Bayes models and the clinical model in predicting clinical outcomes at different visit intervals over a 2-year term using the non-cumulative approach. This table represent the MCC values of the AUC values presented in table 2A in the main manuscript and has been called beta to maintain the A-Z hierarchy of the of other tables S4.

|  | **Prediction at 2 years term** | | | | | | |
| --- | --- | --- | --- | --- | --- | --- | --- |
| **Visit** | **XGboost** | **GRU** | **LogisticRegression** | **LightGBM** | **LSTM** | **Naïve Bayes** | **Clinical model** |
| Baseline | 0.30 (CI: 0.27-0.33) | 0.26 (CI: 0.23-0.28) | 0.26 (CI: 0.24-0.29) | **0.32 (CI: 0.29-0.34)** | 0.27 (CI: 0.24-0.30) | -0.01 (CI: -0.03-0.01) | 0.21 (CI: 0.18-0.23) |
| 1-year | 0.52 (CI: 0.50-0.54) | 0.46 (CI: 0.43-0.48) | 0.44 (CI: 0.41-0.46) | **0.55 (CI: 0.53-0.57)** | 0.46 (CI: 0.44-0.49) | 0.18 (CI: 0.15-0.20) | 0.32 (CI: 0.29-0.34) |
| 2-year | **0.56 (CI: 0.54-0.59)** | 0.56 (CI: 0.53-0.58) | 0.49 (CI: 0.46-0.51) | 0.56 (CI: 0.53-0.58) | 0.55 (CI: 0.52-0.58) | 0.13 (CI: 0.09-0.16) | 0.37 (CI: 0.35-0.40) |
| 3-year | 0.59 (CI: 0.56-0.62) | 0.58 (CI: 0.55-0.61) | 0.51 (CI: 0.48-0.54) | **0.60 (CI: 0.57-0.63)** | 0.57 (CI: 0.54-0.61) | 0.10 (CI: 0.06-0.14) | 0.29 (CI: 0.27-0.32) |
| 4-year | **0.63 (CI: 0.59-0.66)** | 0.56 (CI: 0.53-0.59) | 0.43 (CI: 0.39-0.47) | 0.62 (CI: 0.59-0.66) | 0.59 (CI: 0.56-0.63) | 0.08 (CI: 0.04-0.12) | 0.34 (CI: 0.32-0.37) |
| 5-year | 0.52 (CI: 0.48-0.57) | 0.53 (CI: 0.50-0.57) | 0.40 (CI: 0.35-0.45) | 0.53 (CI: 0.49-0.58) | **0.55 (CI: 0.50-0.59)** | 0.08 (CI: 0.03-0.12) | 0.21 (CI: 0.18-0.24) |
| Average | 0.52 (CI: 0.49-0.55) | 0.49 (CI: 0.46-0.52) | 0.42 (CI: 0.39-0.45) | **0.53 (CI: 0.50-0.56)** | 0.50 (CI: 0.47-0.53) | 0.09 (CI: 0.06-0.13) | 0.29 (CI: 0.27-0.32) |

**Table S4A**: MCC values with 95% Confidence Intervals (CI) rounded at 2 digits for XGBoost, GRU, Logistic Regression, LightGBM, LSTM, Naïve Bayes models and the clinical model in predicting clinical outcomes at different visit intervals over a 5-year term using the cumulative approach.

|  | **Prediction at 5 years term** | | | | | | |
| --- | --- | --- | --- | --- | --- | --- | --- |
| **Visit** | **XGboost** | **GRU** | **LogisticRegression** | **LightGBM** | **LSTM** | **Naïve Bayes** | **Clinical model** |
| Baseline | **0.48 (CI: 0.45-0.50)** | 0.33 (CI: 0.31-0.35) | 0.33 (CI: 0.30-0.36) | 0.44 (CI: 0.42-0.47) | 0.31 (CI: 0.28-0.34) | 0.20 (CI: 0.17-0.23) | 0.28 (CI: 0.26-0.31) |
| 1-year | **0.55 (CI: 0.53-0.57)** | 0.52 (CI: 0.49-0.54) | 0.45 (CI: 0.42-0.47) | 0.53 (CI: 0.51-0.55) | 0.49 (CI: 0.47-0.52) | 0.15 (CI: 0.12-0.18) | 0.32 (CI: 0.29-0.35) |
| 2-year | **0.57 (CI: 0.55-0.60)** | 0.50 (CI: 0.47-0.52) | 0.48 (CI: 0.45-0.51) | 0.55 (CI: 0.53-0.57) | 0.49 (CI: 0.46-0.51) | 0.24 (CI: 0.20-0.28) | 0.30 (CI: 0.27-0.33) |
| 3-year | 0.50 (CI: 0.47-0.53) | 0.49 (CI: 0.46-0.52) | 0.41 (CI: 0.38-0.44) | **0.51 (CI: 0.48-0.53)** | 0.49 (CI: 0.46-0.52) | 0.30 (CI: 0.26-0.33) | 0.29 (CI: 0.26-0.32) |
| 4-year | 0.36 (CI: 0.32-0.40) | **0.42 (CI: 0.38-0.46)** | 0.41 (CI: 0.37-0.45) | 0.33 (CI: 0.29-0.37) | 0.41 (CI: 0.37-0.46) | 0.18 (CI: 0.14-0.22) | 0.29 (CI: 0.26-0.33) |
| 5-year | 0.52 (CI: 0.48-0.57) | 0.50 (CI: 0.46-0.55) | **0.57 (CI: 0.52-0.61)** | 0.50 (CI: 0.46-0.55) | 0.48 (CI: 0.43-0.53) | 0.04 (CI: -0.01-0.09) | 0.23 (CI: 0.18-0.28) |
| Average | **0.50 (CI: 0.47-0.53)** | 0.46 (CI: 0.43-0.49) | 0.44 (CI: 0.41-0.47) | 0.48 (CI: 0.45-0.51) | 0.45 (CI: 0.41-0.48) | 0.19 (CI: 0.15-0.21) | 0.29 (CI: 0.25-0.32) |

**Table S4B**: MCC values with 95% Confidence Intervals (CI) rounded at 2 digits for XGBoost, GRU, Logistic Regression, LightGBM, LSTM, Naïve Bayes models and the clinical model in predicting clinical outcomes at different visit intervals over a 2-year term using the cumulative approach.

|  | **Prediction at 2 years term** | | | | | | |
| --- | --- | --- | --- | --- | --- | --- | --- |
| **Visit** | **XGboost** | **GRU** | **LogisticRegression** | **LightGBM** | **LSTM** | **Naïve Bayes** | **Clinical model** |
| Baseline | 0.20 (CI: 0.17-0.22) | 0.26 (CI: 0.23-0.28) | 0.19 (CI: 0.16-0.22) | 0.20 (CI: 0.17-0.23) | **0.27 (CI: 0.24-0.30)** | -0.03 (CI: -0.06--0.00) | -0.02 (CI: -0.05-0.00) |
| 1-year | **0.54 (CI: 0.52-0.57)** | 0.46 (CI: 0.43-0.48) | 0.49 (CI: 0.47-0.52) | **0.54 (CI: 0.52-0.57)** | 0.46 (CI: 0.44-0.49) | 0.10 (CI: 0.07-0.13) | 0.29 (CI: 0.26-0.32) |
| 2-year | **0.59 (CI: 0.57-0.61)** | 0.56 (CI: 0.53-0.58) | 0.55 (CI: 0.52-0.57) | 0.57 (CI: 0.55-0.60) | 0.55 (CI: 0.52-0.58) | 0.11 (CI: 0.08-0.14) | 0.31 (CI: 0.28-0.34) |
| 3-year | 0.62 (CI: 0.59-0.65) | 0.58 (CI: 0.55-0.61) | 0.56 (CI: 0.52-0.59) | **0.63 (CI: 0.60-0.65)** | 0.57 (CI: 0.54-0.61) | 0.07 (CI: 0.04-0.11) | 0.34 (CI: 0.31-0.37) |
| 4-year | **0.63 (CI: 0.60-0.67)** | 0.56 (CI: 0.53-0.59) | 0.51 (CI: 0.48-0.55) | 0.63 (CI: 0.60-0.66) | 0.59 (CI: 0.56-0.63) | 0.08 (CI: 0.03-0.13) | 0.43 (CI: 0.39-0.47) |
| 5-year | 0.40 (CI: 0.35-0.45) | 0.53 (CI: 0.50-0.57) | 0.49 (CI: 0.45-0.54) | 0.41 (CI: 0.36-0.47) | **0.55 (CI: 0.50-0.59)** | -0.02 (CI: -0.08-0.04) | 0.22 (CI: 0.17-0.27) |
| Average | **0.50 (CI: 0.47-0.53)** | 0.49 (CI: 0.46-0.52) | 0.47 (CI: 0.43-0.50) | **0.50 (CI: 0.47-0.53)** | **0.50 (CI: 0.47-0.53)** | 0.05 (CI: 0.04-0.09) | 0.26 (CI: 0.24-0.30) |

**Table S4C**: MCC values with 95% Confidence Intervals (CI) rounded at 2 digits for XGBoost, GRU, Logistic Regression, LightGBM, LSTM, Naïve Bayes models and the clinical model in predicting clinical outcomes of the population strictly above 68 years old (median age of the cohort) at different visit intervals over a 5-year term using the non-cumulative approach.

|  | **Prediction at 5 years term** | | | | | | |
| --- | --- | --- | --- | --- | --- | --- | --- |
| **Visit** | **XGboost** | **GRU** | **LogisticRegression** | **LightGBM** | **LSTM** | **Naïve Bayes** | **Clinical model** |
| Baseline | **0.45 (CI: 0.41-0.49)** | 0.23 (CI: 0.19-0.27) | 0.27 (CI: 0.23-0.31) | 0.45 (CI: 0.40-0.49) | 0.23 (CI: 0.19-0.27) | 0.01 (CI: -0.02-0.04) | 0.20 (CI: 0.17-0.24) |
| 1-year | **0.54 (CI: 0.51-0.58)** | 0.46 (CI: 0.43-0.50) | 0.40 (CI: 0.36-0.44) | 0.54 (CI: 0.50-0.57) | 0.44 (CI: 0.40-0.47) | 0.14 (CI: 0.11-0.17) | 0.31 (CI: 0.28-0.35) |
| 2-year | **0.54 (CI: 0.51-0.58)** | 0.47 (CI: 0.43-0.50) | 0.43 (CI: 0.39-0.48) | **0.54 (CI: 0.51-0.58)** | 0.44 (CI: 0.40-0.48) | 0.09 (CI: 0.05-0.13) | 0.37 (CI: 0.33-0.42) |
| 3-year | 0.57 (CI: 0.53-0.62) | 0.48 (CI: 0.43-0.52) | 0.41 (CI: 0.36-0.46) | **0.59 (CI: 0.55-0.63)** | 0.46 (CI: 0.42-0.51) | 0.13 (CI: 0.09-0.18) | 0.20 (CI: 0.14-0.26) |
| 4-year | 0.39 (CI: 0.32-0.45) | 0.33 (CI: 0.27-0.40) | 0.31 (CI: 0.24-0.37) | **0.40 (CI: 0.34-0.47)** | 0.32 (CI: 0.25-0.38) | 0.10 (CI: 0.06-0.15) | 0.27 (CI: 0.20-0.33) |
| 5-year | 0.42 (CI: 0.34-0.51) | 0.40 (CI: 0.32-0.48) | 0.35 (CI: 0.27-0.43) | **0.44 (CI: 0.36-0.52)** | 0.34 (CI: 0.26-0.41) | 0.08 (CI: 0.02-0.16) | 0.32 (CI: 0.24-0.39) |
| Average | **0.49 (CI: 0.44-0.54)** | 0.40 (CI: 0.35-0.45) | 0.36 (CI: 0.31-0.42) | **0.49 (CI: 0.44-0.54)** | 0.37 (CI: 0.32-0.42) | 0.09 (CI: 0.06-0.14) | 0.28 (CI: 0.23-0.33) |

**Table S4D**: MCC values with 95% Confidence Intervals (CI) rounded at 2 digits for XGBoost, GRU, Logistic Regression, LightGBM, LSTM, Naïve Bayes models and the clinical model in predicting clinical outcomes of the population strictly above 68 years old (median age of the cohort) at different visit intervals over a 2-year term using the non-cumulative approach.

|  | **Prediction at 2 years term** | | | | | | |
| --- | --- | --- | --- | --- | --- | --- | --- |
| **Visit** | **XGboost** | **GRU** | **LogisticRegression** | **LightGBM** | **LSTM** | **Naïve Bayes** | **Clinical model** |
| Baseline | 0.30 (CI: 0.26-0.34) | 0.26 (CI: 0.22-0.29) | 0.23 (CI: 0.19-0.26) | **0.33 (CI: 0.29-0.36)** | 0.28 (CI: 0.24-0.32) | -0.02 (CI: -0.04-0.00) | 0.18 (CI: 0.14-0.22) |
| 1-year | 0.54 (CI: 0.50-0.57) | 0.45 (CI: 0.41-0.48) | 0.40 (CI: 0.36-0.44) | **0.58 (CI: 0.55-0.61)** | 0.43 (CI: 0.40-0.47) | 0.15 (CI: 0.11-0.18) | 0.37 (CI: 0.33-0.41) |
| 2-year | **0.62 (CI: 0.58-0.66)** | 0.56 (CI: 0.53-0.59) | 0.46 (CI: 0.42-0.49) | 0.62 (CI: 0.58-0.65) | 0.56 (CI: 0.52-0.59) | 0.19 (CI: 0.15-0.22) | 0.36 (CI: 0.32-0.40) |
| 3-year | 0.64 (CI: 0.60-0.68) | 0.60 (CI: 0.56-0.64) | 0.51 (CI: 0.46-0.56) | **0.66 (CI: 0.62-0.70)** | 0.58 (CI: 0.53-0.62) | 0.16 (CI: 0.11-0.21) | 0.35 (CI: 0.30-0.40) |
| 4-year | **0.57 (CI: 0.52-0.63)** | 0.50 (CI: 0.44-0.56) | 0.29 (CI: 0.24-0.36) | 0.55 (CI: 0.50-0.61) | 0.52 (CI: 0.46-0.58) | 0.08 (CI: 0.04-0.12) | 0.47 (CI: 0.40-0.53) |
| 5-year | **0.49 (CI: 0.42-0.56)** | 0.51 (CI: 0.43-0.58) | 0.36 (CI: 0.28-0.43) | 0.49 (CI: 0.42-0.55) | 0.50 (CI: 0.43-0.57) | 0.07 (CI: -0.00-0.14) | 0.38 (CI: 0.30-0.46) |
| Average | 0.53 (CI: 0.48-0.57) | 0.48 (CI: 0.43-0.52) | 0.38 (CI: 0.33-0.42) | **0.54 (CI: 0.49-0.58)** | 0.48 (CI: 0.43-0.53) | 0.11 (CI: 0.07-0.13) | 0.35 (CI: 0.30-0.40) |

**Table S4E**: MCC values with 95% Confidence Intervals (CI) rounded at 2 digits for XGBoost, GRU, Logistic Regression, LightGBM, LSTM, Naïve Bayes models and the clinical model in predicting clinical outcomes of the population under or equal to 68 years old (median age of the cohort) at different visit intervals over a 5-year term using the non-cumulative approach.

|  | **Prediction at 5 years term** | | | | | | |
| --- | --- | --- | --- | --- | --- | --- | --- |
| **Visit** | **XGboost** | **GRU** | **LogisticRegression** | **LightGBM** | **LSTM** | **Naïve Bayes** | **Clinical model** |
| Baseline | **0.49 (CI: 0.45-0.52)** | 0.40 (CI: 0.37-0.43) | 0.39 (CI: 0.35-0.43) | 0.48 (CI: 0.45-0.51) | 0.37 (CI: 0.33-0.40) | 0.04 (CI: 0.02-0.06) | 0.35 (CI: 0.32-0.39) |
| 1-year | 0.51 (CI: 0.47-0.54) | **0.56 (CI: 0.53-0.60)** | 0.48 (CI: 0.44-0.51) | 0.52 (CI: 0.49-0.56) | 0.53 (CI: 0.49-0.57) | 0.18 (CI: 0.14-0.21) | 0.25 (CI: 0.21-0.30) |
| 2-year | **0.53 (CI: 0.50-0.57)** | 0.52 (CI: 0.48-0.57) | 0.48 (CI: 0.44-0.52) | 0.51 (CI: 0.47-0.55) | 0.52 (CI: 0.48-0.56) | 0.04 (CI: -0.00-0.08) | 0.40 (CI: 0.36-0.45) |
| 3-year | **0.51 (CI: 0.47-0.56)** | 0.50 (CI: 0.46-0.54) | 0.48 (CI: 0.43-0.52) | 0.51 (CI: 0.46-0.56) | **0.51 (CI: 0.47-0.56)** | 0.02 (CI: -0.02-0.07) | 0.16 (CI: 0.10-0.22) |
| 4-year | **0.50 (CI: 0.45-0.56)** | 0.48 (CI: 0.42-0.54) | 0.47 (CI: 0.41-0.53) | 0.47 (CI: 0.41-0.53) | 0.49 (CI: 0.43-0.56) | 0.08 (CI: 0.05-0.11) | 0.32 (CI: 0.25-0.39) |
| 5-year | 0.42 (CI: 0.35-0.50) | 0.55 (CI: 0.49-0.61) | 0.46 (CI: 0.40-0.53) | 0.44 (CI: 0.37-0.51) | **0.55 (CI: 0.49-0.62)** | 0.05 (CI: 0.02-0.07) | 0.27 (CI: 0.20-0.34) |
| Average | 0.49 (CI: 0.45-0.54) | **0.50 (CI: 0.46-0.55)** | 0.46 (CI: 0.41-0.51) | 0.49 (CI: 0.44-0.54) | 0.50 (CI: 0.45-0.55) | 0.07 (CI: 0.04-0.08) | 0.29 (CI: 0.24-0.35) |

**Table S4F**: MCC values with 95% Confidence Intervals (CI) rounded at 2 digits for XGBoost, GRU, Logistic Regression, LightGBM, LSTM, Naïve Bayes models and the clinical model in predicting clinical outcomes of the population under or equal to 68 years old (median age of the cohort) at different visit intervals over a 2-year term using the non-cumulative approach.

|  | **Prediction at 2 years term** | | | | | | |
| --- | --- | --- | --- | --- | --- | --- | --- |
| **Visit** | **XGboost** | **GRU** | **LogisticRegression** | **LightGBM** | **LSTM** | **Naïve Bayes** | **Clinical model** |
| Baseline | **0.31 (CI: 0.27-0.35)** | 0.27 (CI: 0.23-0.31) | 0.29 (CI: 0.25-0.33) | **0.31 (CI: 0.27-0.35)** | 0.26 (CI: 0.22-0.31) | 0.01 (CI: -0.01-0.03) | 0.23 (CI: 0.20-0.27) |
| 1-year | 0.51 (CI: 0.48-0.54) | 0.45 (CI: 0.42-0.49) | 0.47 (CI: 0.44-0.51) | **0.51 (CI: 0.48-0.55)** | 0.48 (CI: 0.44-0.51) | 0.17 (CI: 0.14-0.21) | 0.25 (CI: 0.22-0.29) |
| 2-year | 0.52 (CI: 0.48-0.56) | **0.56 (CI: 0.52-0.59)** | 0.51 (CI: 0.48-0.55) | 0.50 (CI: 0.47-0.54) | 0.54 (CI: 0.50-0.58) | 0.06 (CI: 0.02-0.10) | 0.42 (CI: 0.38-0.46) |
| 3-year | 0.54 (CI: 0.50-0.58) | **0.58 (CI: 0.54-0.63)** | 0.51 (CI: 0.46-0.55) | 0.55 (CI: 0.51-0.59) | 0.57 (CI: 0.53-0.62) | 0.02 (CI: -0.03-0.06) | 0.33 (CI: 0.28-0.39) |
| 4-year | 0.68 (CI: 0.63-0.73) | 0.63 (CI: 0.57-0.68) | 0.56 (CI: 0.51-0.61) | **0.69 (CI: 0.64-0.74)** | 0.64 (CI: 0.59-0.69) | 0.05 (CI: 0.01-0.09) | 0.53 (CI: 0.46-0.60) |
| 5-year | 0.55 (CI: 0.49-0.62) | 0.54 (CI: 0.48-0.61) | 0.44 (CI: 0.37-0.51) | **0.56 (CI: 0.50-0.63)** | 0.56 (CI: 0.50-0.62) | 0.05 (CI: 0.03-0.08) | 0.40 (CI: 0.31-0.48) |
| Average | 0.52 (CI: 0.48-0.56) | 0.51 (CI: 0.46-0.55) | 0.46 (CI: 0.42-0.51) | **0.52 (CI: 0.48-0.57)** | 0.51 (CI: 0.46-0.56) | 0.06 (CI: 0.03-0.09) | 0.36 (CI: 0.31-0.42) |

**Table S4G**: MCC values with 95% Confidence Intervals (CI) rounded at 2 digits for XGBoost, GRU, Logistic Regression, LightGBM, LSTM, Naïve Bayes models and the clinical model in predicting clinical outcomes of the population strictly above 68 years old (median age of the cohort) at different visit intervals over a 5-year term using the cumulative approach.

|  | **Prediction at 5 years term** | | | | | | |
| --- | --- | --- | --- | --- | --- | --- | --- |
| **Visit** | **XGboost** | **GRU** | **LogisticRegression** | **LightGBM** | **LSTM** | **Naïve Bayes** | **Clinical model** |
| Baseline | **0.45 (CI: 0.41-0.49)** | 0.23 (CI: 0.19-0.27) | 0.28 (CI: 0.24-0.31) | 0.38 (CI: 0.34-0.42) | 0.23 (CI: 0.19-0.27) | 0.12 (CI: 0.08-0.16) | 0.19 (CI: 0.15-0.22) |
| 1-year | **0.53 (CI: 0.50-0.56)** | 0.46 (CI: 0.43-0.50) | 0.40 (CI: 0.37-0.44) | 0.52 (CI: 0.49-0.56) | 0.44 (CI: 0.40-0.47) | 0.12 (CI: 0.09-0.16) | 0.28 (CI: 0.24-0.32) |
| 2-year | **0.54 (CI: 0.50-0.57)** | 0.47 (CI: 0.43-0.50) | 0.45 (CI: 0.41-0.50) | 0.51 (CI: 0.47-0.55) | 0.44 (CI: 0.40-0.48) | 0.21 (CI: 0.16-0.26) | 0.26 (CI: 0.22-0.30) |
| 3-year | **0.52 (CI: 0.48-0.56)** | 0.48 (CI: 0.43-0.52) | 0.38 (CI: 0.33-0.43) | 0.51 (CI: 0.47-0.56) | 0.46 (CI: 0.42-0.51) | 0.24 (CI: 0.18-0.30) | 0.26 (CI: 0.21-0.31) |
| 4-year | 0.31 (CI: 0.25-0.37) | **0.33 (CI: 0.27-0.40)** | 0.28 (CI: 0.22-0.34) | 0.26 (CI: 0.19-0.33) | 0.32 (CI: 0.25-0.38) | 0.10 (CI: 0.04-0.16) | 0.24 (CI: 0.19-0.30) |
| 5-year | 0.45 (CI: 0.38-0.52) | 0.40 (CI: 0.32-0.48) | **0.46 (CI: 0.39-0.54)** | **0.46 (CI: 0.39-0.54)** | 0.34 (CI: 0.26-0.41) | -0.04 (CI: -0.11-0.02) | 0.12 (CI: 0.05-0.19) |
| Average | **0.47 (CI: 0.42-0.51)** | 0.40 (CI: 0.35-0.45) | 0.38 (CI: 0.33-0.43) | 0.44 (CI: 0.39-0.49) | 0.37 (CI: 0.32-0.42) | 0.13 (CI: 0.08-0.18) | 0.23 (CI: 0.18-0.27) |

**Table S4H**: MCC values with 95% Confidence Intervals (CI) rounded at 2 digits for XGBoost, GRU, Logistic Regression, LightGBM, LSTM, Naïve Bayes models and the clinical model in predicting clinical outcomes of the population strictly above 68 years old (median age of the cohort) at different visit intervals over a 2-year term using the cumulative approach.

|  | **Prediction at 2 years term** | | | | | | |
| --- | --- | --- | --- | --- | --- | --- | --- |
| **Visit** | **XGboost** | **GRU** | **LogisticRegression** | **LightGBM** | **LSTM** | **Naïve Bayes** | **Clinical model** |
| Baseline | 0.17 (CI: 0.13-0.22) | 0.26 (CI: 0.22-0.29) | 0.14 (CI: 0.09-0.18) | 0.20 (CI: 0.15-0.25) | **0.28 (CI: 0.24-0.32)** | -0.06 (CI: -0.10--0.01) | 0.00 (CI: -0.03-0.04) |
| 1-year | 0.53 (CI: 0.49-0.57) | 0.45 (CI: 0.41-0.48) | 0.47 (CI: 0.43-0.52) | **0.54 (CI: 0.50-0.57)** | 0.43 (CI: 0.40-0.47) | 0.08 (CI: 0.04-0.12) | 0.30 (CI: 0.26-0.34) |
| 2-year | **0.57 (CI: 0.54-0.61)** | 0.56 (CI: 0.53-0.59) | 0.54 (CI: 0.51-0.58) | 0.56 (CI: 0.52-0.60) | 0.56 (CI: 0.52-0.59) | 0.08 (CI: 0.03-0.12) | 0.36 (CI: 0.32-0.40) |
| 3-year | **0.65 (CI: 0.62-0.69)** | 0.60 (CI: 0.56-0.64) | 0.53 (CI: 0.48-0.58) | 0.65 (CI: 0.61-0.69) | 0.58 (CI: 0.53-0.62) | 0.13 (CI: 0.07-0.18) | 0.37 (CI: 0.32-0.42) |
| 4-year | **0.56 (CI: 0.50-0.62)** | 0.50 (CI: 0.44-0.56) | 0.44 (CI: 0.38-0.50) | 0.53 (CI: 0.48-0.59) | 0.52 (CI: 0.46-0.58) | 0.03 (CI: -0.04-0.09) | 0.23 (CI: 0.17-0.29) |
| 5-year | 0.41 (CI: 0.33-0.49) | **0.51 (CI: 0.43-0.58)** | 0.44 (CI: 0.36-0.52) | 0.42 (CI: 0.34-0.50) | 0.50 (CI: 0.43-0.57) | -0.03 (CI: -0.11-0.05) | 0.07 (CI: -0.00-0.15) |
| Average | **0.48 (CI: 0.44-0.53)** | 0.48 (CI: 0.43-0.52) | 0.43 (CI: 0.38-0.48) | 0.48 (CI: 0.43-0.53) | 0.48 (CI: 0.43-0.53) | 0.04 (CI: -0.01-0.09) | 0.22 (CI: 0.18-0.25) |

**Table S4I**: MCC values with 95% Confidence Intervals (CI) rounded at 2 digits for XGBoost, GRU, Logistic Regression, LightGBM, LSTM, Naïve Bayes models and the clinical model in predicting clinical outcomes of the population under or equal to 68 years old (median age of the cohort) at different visit intervals over a 5-year term using the cumulative approach.

|  | **Prediction at 5 years term** | | | | | | |
| --- | --- | --- | --- | --- | --- | --- | --- |
| **Visit** | **XGboost** | **GRU** | **LogisticRegression** | **LightGBM** | **LSTM** | **Naïve Bayes** | **Clinical model** |
| Baseline | 0.48 (CI: 0.44-0.51) | 0.40 (CI: 0.37-0.43) | 0.37 (CI: 0.33-0.41) | **0.48 (CI: 0.45-0.51)** | 0.37 (CI: 0.33-0.40) | 0.23 (CI: 0.20-0.27) | 0.35 (CI: 0.31-0.39) |
| 1-year | 0.56 (CI: 0.53-0.58) | **0.56 (CI: 0.53-0.60)** | 0.48 (CI: 0.44-0.52) | 0.53 (CI: 0.50-0.56) | 0.53 (CI: 0.49-0.57) | 0.16 (CI: 0.11-0.20) | 0.34 (CI: 0.30-0.38) |
| 2-year | **0.61 (CI: 0.57-0.64)** | 0.52 (CI: 0.48-0.57) | 0.50 (CI: 0.46-0.53) | 0.59 (CI: 0.55-0.62) | 0.52 (CI: 0.48-0.56) | 0.25 (CI: 0.20-0.31) | 0.31 (CI: 0.27-0.36) |
| 3-year | 0.47 (CI: 0.43-0.52) | 0.50 (CI: 0.46-0.54) | 0.43 (CI: 0.39-0.48) | 0.49 (CI: 0.45-0.54) | **0.51 (CI: 0.47-0.56)** | 0.33 (CI: 0.29-0.38) | 0.33 (CI: 0.28-0.38) |
| 4-year | 0.41 (CI: 0.36-0.47) | 0.48 (CI: 0.42-0.54) | **0.53 (CI: 0.47-0.58)** | 0.39 (CI: 0.33-0.44) | 0.49 (CI: 0.43-0.56) | 0.25 (CI: 0.19-0.31) | 0.35 (CI: 0.29-0.42) |
| 5-year | 0.51 (CI: 0.44-0.58) | 0.55 (CI: 0.49-0.61) | **0.60 (CI: 0.54-0.66)** | 0.46 (CI: 0.39-0.53) | 0.55 (CI: 0.49-0.62) | 0.11 (CI: 0.04-0.18) | 0.27 (CI: 0.19-0.34) |
| Average | **0.51 (CI: 0.46-0.55)** | 0.50 (CI: 0.46-0.55) | 0.49 (CI: 0.44-0.53) | 0.49 (CI: 0.45-0.53) | 0.50 (CI: 0.45-0.55) | 0.22 (CI: 0.17-0.28) | 0.33 (CI: 0.27-0.38) |

**Table S4J**: MCC values with 95% Confidence Intervals (CI) rounded at 2 digits for XGBoost, GRU, Logistic Regression, LightGBM, LSTM, Naïve Bayes models and the clinical model in predicting clinical outcomes of the population under or equal to 68 years old (median age of the cohort) at different visit intervals over a 2-year term using the cumulative approach.

|  | **Prediction at 2 years term** | | | | | | |
| --- | --- | --- | --- | --- | --- | --- | --- |
| **Visit** | **XGboost** | **GRU** | **LogisticRegression** | **LightGBM** | **LSTM** | **Naïve Bayes** | **Clinical model** |
| Baseline | 0.22 (CI: 0.18-0.26) | **0.27 (CI: 0.23-0.31)** | 0.24 (CI: 0.20-0.28) | 0.21 (CI: 0.17-0.25) | 0.26 (CI: 0.22-0.31) | -0.01 (CI: -0.05-0.02) | -0.05 (CI: -0.07--0.03) |
| 1-year | **0.55 (CI: 0.51-0.58)** | 0.45 (CI: 0.42-0.49) | 0.50 (CI: 0.47-0.54) | 0.54 (CI: 0.50-0.57) | 0.48 (CI: 0.44-0.51) | 0.11 (CI: 0.07-0.15) | 0.26 (CI: 0.23-0.30) |
| 2-year | **0.60 (CI: 0.56-0.63)** | 0.56 (CI: 0.52-0.59) | 0.55 (CI: 0.52-0.59) | 0.58 (CI: 0.54-0.62) | 0.54 (CI: 0.50-0.58) | 0.14 (CI: 0.10-0.19) | 0.25 (CI: 0.21-0.28) |
| 3-year | 0.58 (CI: 0.54-0.63) | 0.58 (CI: 0.54-0.63) | 0.57 (CI: 0.53-0.62) | **0.60 (CI: 0.56-0.65)** | 0.57 (CI: 0.53-0.62) | 0.01 (CI: -0.05-0.06) | 0.31 (CI: 0.26-0.37) |
| 4-year | 0.69 (CI: 0.64-0.74) | 0.63 (CI: 0.57-0.68) | 0.57 (CI: 0.52-0.62) | **0.72 (CI: 0.68-0.76)** | 0.64 (CI: 0.59-0.69) | 0.12 (CI: 0.05-0.19) | 0.63 (CI: 0.58-0.67) |
| 5-year | 0.39 (CI: 0.32-0.46) | 0.54 (CI: 0.48-0.61) | 0.53 (CI: 0.47-0.60) | 0.40 (CI: 0.33-0.48) | **0.56 (CI: 0.50-0.62)** | -0.00 (CI: -0.08-0.07) | 0.30 (CI: 0.23-0.37) |
| Average | 0.51 (CI: 0.46-0.55) | 0.51 (CI: 0.46-0.55) | 0.49 (CI: 0.45-0.54) | **0.51 (CI: 0.46-0.56)** | **0.51 (CI: 0.46-0.56)** | 0.06 (CI: 0.04-0.10) | 0.28 (CI: 0.25-0.34) |

**Table S4K**: MCC values with 95% Confidence Intervals (CI) rounded at 2 digits for XGBoost, GRU, Logistic Regression, LightGBM, LSTM, Naïve Bayes models and the clinical model in predicting clinical outcomes of the men population at different visit intervals over a 5-year term using the non-cumulative approach.

|  | **Prediction at 5 years term** | | | | | | |
| --- | --- | --- | --- | --- | --- | --- | --- |
| **Visit** | **XGboost** | **GRU** | **LogisticRegression** | **LightGBM** | **LSTM** | **Naïve Bayes** | **Clinical model** |
| Baseline | **0.46 (CI: 0.43-0.48)** | 0.34 (CI: 0.31-0.37) | 0.33 (CI: 0.30-0.36) | 0.45 (CI: 0.43-0.48) | 0.31 (CI: 0.28-0.34) | 0.02 (CI: -0.00-0.05) | 0.35 (CI: 0.32-0.37) |
| 1-year | 0.51 (CI: 0.48-0.54) | **0.52 (CI: 0.49-0.55)** | 0.46 (CI: 0.43-0.50) | **0.52 (CI: 0.49-0.55)** | 0.49 (CI: 0.46-0.52) | 0.15 (CI: 0.12-0.18) | 0.33 (CI: 0.30-0.37) |
| 2-year | **0.54 (CI: 0.51-0.57)** | 0.51 (CI: 0.48-0.54) | 0.48 (CI: 0.44-0.51) | **0.54 (CI: 0.51-0.57)** | 0.49 (CI: 0.46-0.52) | 0.07 (CI: 0.03-0.10) | 0.38 (CI: 0.35-0.42) |
| 3-year | 0.52 (CI: 0.48-0.56) | 0.49 (CI: 0.46-0.53) | 0.45 (CI: 0.42-0.49) | **0.54 (CI: 0.50-0.58)** | 0.49 (CI: 0.45-0.52) | 0.03 (CI: -0.02-0.07) | 0.17 (CI: 0.13-0.21) |
| 4-year | 0.39 (CI: 0.34-0.44) | **0.40 (CI: 0.36-0.45)** | 0.37 (CI: 0.32-0.42) | 0.39 (CI: 0.35-0.44) | **0.40 (CI: 0.36-0.45)** | 0.06 (CI: 0.03-0.10) | 0.31 (CI: 0.25-0.36) |
| 5-year | 0.27 (CI: 0.21-0.34) | **0.39 (CI: 0.33-0.45)** | 0.33 (CI: 0.27-0.40) | 0.28 (CI: 0.22-0.34) | 0.37 (CI: 0.31-0.42) | 0.10 (CI: 0.05-0.16) | 0.35 (CI: 0.29-0.41) |
| Average | 0.45 (CI: 0.41-0.49) | 0.44 (CI: 0.41-0.48) | 0.40 (CI: 0.36-0.45) | **0.45 (CI: 0.42-0.49)** | 0.43 (CI: 0.39-0.46) | 0.07 (CI: 0.04-0.09) | 0.32 (CI: 0.27-0.36) |

**Table S4L**: MCC values with 95% Confidence Intervals (CI) rounded at 2 digits for XGBoost, GRU, Logistic Regression, LightGBM, LSTM, Naïve Bayes models and the clinical model in predicting clinical outcomes of the men population at different visit intervals over a 2-year term using the non-cumulative approach.

|  | **Prediction at 2 years term** | | | | | | |
| --- | --- | --- | --- | --- | --- | --- | --- |
| **Visit** | **XGboost** | **GRU** | **LogisticRegression** | **LightGBM** | **LSTM** | **Naïve Bayes** | **Clinical model** |
| Baseline | 0.32 (CI: 0.29-0.35) | 0.26 (CI: 0.23-0.29) | 0.29 (CI: 0.26-0.32) | **0.34 (CI: 0.31-0.37)** | 0.27 (CI: 0.24-0.30) | -0.01 (CI: -0.04-0.01) | 0.23 (CI: 0.21-0.26) |
| 1-year | 0.51 (CI: 0.49-0.54) | 0.47 (CI: 0.44-0.51) | 0.44 (CI: 0.41-0.47) | **0.54 (CI: 0.51-0.57)** | 0.48 (CI: 0.45-0.51) | 0.15 (CI: 0.12-0.18) | 0.29 (CI: 0.26-0.33) |
| 2-year | 0.54 (CI: 0.51-0.57) | **0.57 (CI: 0.55-0.60)** | 0.48 (CI: 0.45-0.51) | 0.53 (CI: 0.50-0.56) | 0.56 (CI: 0.53-0.59) | 0.12 (CI: 0.09-0.15) | 0.38 (CI: 0.35-0.41) |
| 3-year | 0.60 (CI: 0.57-0.64) | 0.60 (CI: 0.57-0.63) | 0.51 (CI: 0.47-0.54) | **0.61 (CI: 0.58-0.65)** | 0.59 (CI: 0.56-0.62) | 0.07 (CI: 0.02-0.11) | 0.35 (CI: 0.31-0.39) |
| 4-year | **0.62 (CI: 0.58-0.66)** | 0.58 (CI: 0.55-0.62) | 0.46 (CI: 0.42-0.50) | **0.62 (CI: 0.58-0.66)** | 0.61 (CI: 0.58-0.65) | 0.05 (CI: 0.01-0.09) | 0.53 (CI: 0.48-0.57) |
| 5-year | 0.42 (CI: 0.36-0.49) | 0.49 (CI: 0.44-0.54) | 0.34 (CI: 0.28-0.39) | 0.44 (CI: 0.38-0.50) | **0.49 (CI: 0.44-0.55)** | 0.09 (CI: 0.03-0.14) | 0.42 (CI: 0.36-0.49) |
| Average | 0.50 (CI: 0.47-0.54) | 0.50 (CI: 0.46-0.53) | 0.42 (CI: 0.38-0.46) | **0.51 (CI: 0.48-0.55)** | 0.50 (CI: 0.47-0.54) | 0.08 (CI: 0.05-0.11) | 0.37 (CI: 0.33-0.41) |

**Table S4M**: MCC values with 95% Confidence Intervals (CI) rounded at 2 digits for XGBoost, GRU, Logistic Regression, LightGBM, LSTM, Naïve Bayes models and the clinical model in predicting clinical outcomes of the women population at different visit intervals over a 5-year term using the non-cumulative approach.

|  | **Prediction at 5 years term** | | | | | | |
| --- | --- | --- | --- | --- | --- | --- | --- |
| **Visit** | **XGboost** | **GRU** | **LogisticRegression** | **LightGBM** | **LSTM** | **Naïve Bayes** | **Clinical model** |
| Baseline | **0.54 (CI: 0.49-0.59)** | 0.31 (CI: 0.25-0.37) | 0.35 (CI: 0.30-0.41) | 0.51 (CI: 0.46-0.56) | 0.31 (CI: 0.26-0.37) | 0.03 (CI: 0.01-0.05) | 0.20 (CI: 0.15-0.26) |
| 1-year | **0.58 (CI: 0.54-0.62)** | 0.51 (CI: 0.46-0.56) | 0.40 (CI: 0.35-0.46) | 0.56 (CI: 0.52-0.61) | 0.48 (CI: 0.43-0.53) | 0.17 (CI: 0.13-0.21) | 0.21 (CI: 0.15-0.26) |
| 2-year | **0.54 (CI: 0.49-0.59)** | 0.48 (CI: 0.42-0.53) | 0.42 (CI: 0.36-0.48) | 0.50 (CI: 0.45-0.55) | 0.45 (CI: 0.40-0.51) | 0.07 (CI: 0.02-0.12) | 0.36 (CI: 0.30-0.43) |
| 3-year | **0.57 (CI: 0.50-0.63)** | 0.48 (CI: 0.41-0.54) | 0.39 (CI: 0.33-0.46) | **0.57 (CI: 0.50-0.63)** | 0.47 (CI: 0.41-0.53) | 0.20 (CI: 0.15-0.25) | 0.20 (CI: 0.13-0.27) |
| 4-year | **0.53 (CI: 0.45-0.61)** | 0.44 (CI: 0.37-0.51) | 0.42 (CI: 0.35-0.50) | 0.50 (CI: 0.43-0.58) | 0.41 (CI: 0.34-0.49) | 0.16 (CI: 0.10-0.21) | 0.20 (CI: 0.12-0.28) |
| 5-year | 0.66 (CI: 0.58-0.74) | 0.61 (CI: 0.53-0.70) | 0.55 (CI: 0.46-0.64) | **0.69 (CI: 0.62-0.77)** | 0.58 (CI: 0.50-0.67) | 0.02 (CI: -0.01-0.04) | 0.21 (CI: 0.12-0.29) |
| Average | **0.57 (CI: 0.51-0.63)** | 0.47 (CI: 0.41-0.54) | 0.42 (CI: 0.36-0.49) | 0.56 (CI: 0.50-0.62) | 0.45 (CI: 0.39-0.52) | 0.11 (CI: 0.07-0.14) | 0.23 (CI: 0.16-0.30) |

**Table S4N**: MCC values with 95% Confidence Intervals (CI) rounded at 2 digits for XGBoost, GRU, Logistic Regression, LightGBM, LSTM, Naïve Bayes models and the clinical model in predicting clinical outcomes of the women population at different visit intervals over a 2-year term using the non-cumulative approach.

|  | **Prediction at 2 years term** | | | | | | |
| --- | --- | --- | --- | --- | --- | --- | --- |
| **Visit** | **XGboost** | **GRU** | **LogisticRegression** | **LightGBM** | **LSTM** | **Naïve Bayes** | **Clinical model** |
| Baseline | 0.22 (CI: 0.16-0.28) | 0.21 (CI: 0.15-0.27) | 0.17 (CI: 0.12-0.23) | **0.24 (CI: 0.18-0.29)** | 0.24 (CI: 0.17-0.30) | -0.01 (CI: -0.02--0.00) | 0.16 (CI: 0.11-0.22) |
| 1-year | 0.54 (CI: 0.50-0.58) | 0.40 (CI: 0.34-0.45) | 0.42 (CI: 0.37-0.47) | **0.56 (CI: 0.52-0.60)** | 0.41 (CI: 0.36-0.46) | 0.19 (CI: 0.14-0.23) | 0.43 (CI: 0.38-0.48) |
| 2-year | 0.59 (CI: 0.54-0.65) | 0.50 (CI: 0.44-0.56) | 0.46 (CI: 0.40-0.52) | **0.60 (CI: 0.55-0.66)** | 0.50 (CI: 0.44-0.56) | 0.12 (CI: 0.07-0.17) | 0.42 (CI: 0.35-0.48) |
| 3-year | 0.54 (CI: 0.49-0.60) | 0.50 (CI: 0.45-0.56) | 0.50 (CI: 0.44-0.56) | **0.55 (CI: 0.49-0.61)** | 0.52 (CI: 0.46-0.58) | 0.12 (CI: 0.07-0.17) | 0.32 (CI: 0.25-0.40) |
| 4-year | 0.56 (CI: 0.49-0.64) | 0.49 (CI: 0.42-0.56) | 0.34 (CI: 0.26-0.42) | **0.58 (CI: 0.50-0.66)** | 0.49 (CI: 0.42-0.57) | 0.08 (CI: 0.05-0.11) | 0.29 (CI: 0.21-0.37) |
| 5-year | **0.60 (CI: 0.52-0.68)** | 0.55 (CI: 0.47-0.62) | 0.45 (CI: 0.37-0.54) | **0.60 (CI: 0.52-0.68)** | 0.54 (CI: 0.46-0.62) | 0.01 (CI: -0.00-0.02) | 0.31 (CI: 0.22-0.39) |
| Average | 0.51 (CI: 0.45-0.57) | 0.44 (CI: 0.38-0.50) | 0.39 (CI: 0.33-0.46) | **0.52 (CI: 0.46-0.58)** | 0.45 (CI: 0.39-0.52) | 0.09 (CI: 0.06-0.12) | 0.32 (CI: 0.25-0.39) |

**Table S4O**: MCC values with 95% Confidence Intervals (CI) rounded at 2 digits for XGBoost, GRU, Logistic Regression, LightGBM, LSTM, Naïve Bayes models and the clinical model in predicting clinical outcomes of the men population at different visit intervals over a 5-year term using the cumulative approach.

|  | **Prediction at 5 years term** | | | | | | |
| --- | --- | --- | --- | --- | --- | --- | --- |
| **Visit** | **XGboost** | **GRU** | **LogisticRegression** | **LightGBM** | **LSTM** | **Naïve Bayes** | **Clinical model** |
| Baseline | **0.48 (CI: 0.44-0.51)** | 0.34 (CI: 0.31-0.37) | 0.31 (CI: 0.28-0.35) | 0.43 (CI: 0.41-0.46) | 0.31 (CI: 0.28-0.34) | 0.19 (CI: 0.16-0.22) | 0.34 (CI: 0.31-0.37) |
| 1-year | **0.55 (CI: 0.52-0.58)** | 0.52 (CI: 0.49-0.55) | 0.44 (CI: 0.42-0.47) | 0.54 (CI: 0.51-0.57) | 0.49 (CI: 0.46-0.52) | 0.14 (CI: 0.11-0.18) | 0.37 (CI: 0.34-0.40) |
| 2-year | **0.59 (CI: 0.56-0.62)** | 0.51 (CI: 0.48-0.54) | 0.52 (CI: 0.49-0.56) | 0.57 (CI: 0.54-0.60) | 0.49 (CI: 0.46-0.52) | 0.27 (CI: 0.22-0.31) | 0.30 (CI: 0.27-0.33) |
| 3-year | 0.51 (CI: 0.47-0.54) | 0.49 (CI: 0.46-0.53) | 0.42 (CI: 0.38-0.46) | **0.52 (CI: 0.48-0.55)** | 0.49 (CI: 0.45-0.52) | 0.34 (CI: 0.30-0.38) | 0.30 (CI: 0.27-0.34) |
| 4-year | 0.34 (CI: 0.29-0.39) | **0.40 (CI: 0.36-0.45)** | **0.40 (CI: 0.36-0.45)** | 0.31 (CI: 0.26-0.35) | **0.40 (CI: 0.36-0.45)** | 0.21 (CI: 0.17-0.26) | 0.32 (CI: 0.28-0.37) |
| 5-year | 0.45 (CI: 0.40-0.51) | 0.39 (CI: 0.33-0.45) | **0.54 (CI: 0.49-0.60)** | 0.47 (CI: 0.41-0.53) | 0.37 (CI: 0.31-0.42) | -0.01 (CI: -0.07-0.06) | 0.17 (CI: 0.11-0.24) |
| Average | **0.49 (CI: 0.45-0.53)** | 0.44 (CI: 0.41-0.48) | 0.44 (CI: 0.40-0.48) | 0.47 (CI: 0.44-0.51) | 0.43 (CI: 0.39-0.46) | 0.19 (CI: 0.16-0.23) | 0.30 (CI: 0.26-0.34) |

**Table S4P**: MCC values with 95% Confidence Intervals (CI) rounded at 2 digits for XGBoost, GRU, Logistic Regression, LightGBM, LSTM, Naïve Bayes models and the clinical model in predicting clinical outcomes of the men population at different visit intervals over a 2-year term using the cumulative approach.

|  | **Prediction at 2 years term** | | | | | | |
| --- | --- | --- | --- | --- | --- | --- | --- |
| **Visit** | **XGboost** | **GRU** | **LogisticRegression** | **LightGBM** | **LSTM** | **Naïve Bayes** | **Clinical model** |
| Baseline | 0.21 (CI: 0.18-0.24) | 0.26 (CI: 0.23-0.29) | 0.18 (CI: 0.15-0.22) | 0.21 (CI: 0.18-0.24) | **0.27 (CI: 0.24-0.30)** | -0.03 (CI: -0.07-0.00) | -0.01 (CI: -0.04-0.01) |
| 1-year | 0.55 (CI: 0.52-0.57) | 0.47 (CI: 0.44-0.51) | 0.53 (CI: 0.50-0.56) | **0.55 (CI: 0.52-0.58)** | 0.48 (CI: 0.45-0.51) | 0.05 (CI: 0.02-0.09) | 0.29 (CI: 0.26-0.32) |
| 2-year | **0.59 (CI: 0.56-0.61)** | 0.57 (CI: 0.55-0.60) | 0.58 (CI: 0.55-0.61) | 0.57 (CI: 0.54-0.60) | 0.56 (CI: 0.53-0.59) | 0.11 (CI: 0.07-0.14) | 0.28 (CI: 0.25-0.31) |
| 3-year | 0.63 (CI: 0.60-0.66) | 0.60 (CI: 0.57-0.63) | 0.56 (CI: 0.53-0.60) | **0.64 (CI: 0.61-0.67)** | 0.59 (CI: 0.56-0.62) | 0.09 (CI: 0.04-0.13) | 0.34 (CI: 0.30-0.38) |
| 4-year | 0.62 (CI: 0.58-0.65) | 0.58 (CI: 0.55-0.62) | 0.56 (CI: 0.53-0.59) | **0.62 (CI: 0.59-0.66)** | 0.61 (CI: 0.58-0.65) | 0.06 (CI: 0.00-0.11) | 0.44 (CI: 0.40-0.48) |
| 5-year | 0.36 (CI: 0.30-0.42) | 0.49 (CI: 0.44-0.54) | 0.45 (CI: 0.39-0.51) | 0.38 (CI: 0.31-0.44) | **0.49 (CI: 0.44-0.55)** | -0.06 (CI: -0.13-0.00) | 0.19 (CI: 0.13-0.26) |
| Average | 0.49 (CI: 0.46-0.53) | 0.50 (CI: 0.46-0.53) | 0.48 (CI: 0.44-0.52) | 0.50 (CI: 0.46-0.53) | **0.50 (CI: 0.47-0.54)** | 0.04 (CI: 0.01-0.09) | 0.26 (CI: 0.22-0.29) |

**Table S4Q**: MCC values with 95% Confidence Intervals (CI) rounded at 2 digits for XGBoost, GRU, Logistic Regression, LightGBM, LSTM, Naïve Bayes models and the clinical model in predicting clinical outcomes of the women population at different visit intervals over a 5-year term using the cumulative approach.

|  | **Prediction at 5 years term** | | | | | | |
| --- | --- | --- | --- | --- | --- | --- | --- |
| **Visit** | **XGboost** | **GRU** | **LogisticRegression** | **LightGBM** | **LSTM** | **Naïve Bayes** | **Clinical model** |
| Baseline | **0.46 (CI: 0.41-0.51)** | 0.31 (CI: 0.25-0.37) | 0.36 (CI: 0.30-0.41) | **0.46 (CI: 0.41-0.51)** | 0.31 (CI: 0.26-0.37) | 0.23 (CI: 0.17-0.28) | 0.18 (CI: 0.13-0.24) |
| 1-year | **0.52 (CI: 0.47-0.56)** | 0.51 (CI: 0.46-0.56) | 0.46 (CI: 0.41-0.52) | 0.50 (CI: 0.45-0.54) | 0.48 (CI: 0.43-0.53) | 0.16 (CI: 0.11-0.21) | 0.22 (CI: 0.16-0.28) |
| 2-year | **0.53 (CI: 0.48-0.58)** | 0.48 (CI: 0.42-0.53) | 0.36 (CI: 0.30-0.42) | 0.50 (CI: 0.44-0.55) | 0.45 (CI: 0.40-0.51) | 0.19 (CI: 0.13-0.24) | 0.33 (CI: 0.27-0.40) |
| 3-year | 0.47 (CI: 0.41-0.53) | **0.48 (CI: 0.41-0.54)** | 0.34 (CI: 0.27-0.41) | 0.46 (CI: 0.40-0.51) | 0.47 (CI: 0.41-0.53) | 0.19 (CI: 0.13-0.26) | 0.31 (CI: 0.24-0.38) |
| 4-year | 0.38 (CI: 0.30-0.45) | **0.44 (CI: 0.37-0.51)** | 0.40 (CI: 0.33-0.48) | 0.34 (CI: 0.26-0.43) | 0.41 (CI: 0.34-0.49) | 0.10 (CI: 0.02-0.18) | 0.19 (CI: 0.12-0.26) |
| 5-year | 0.52 (CI: 0.44-0.61) | **0.61 (CI: 0.53-0.70)** | 0.48 (CI: 0.40-0.57) | 0.45 (CI: 0.36-0.54) | 0.58 (CI: 0.50-0.67) | 0.13 (CI: 0.06-0.20) | 0.29 (CI: 0.22-0.36) |
| Average | **0.48 (CI: 0.42-0.54)** | 0.47 (CI: 0.41-0.54) | 0.40 (CI: 0.34-0.47) | 0.45 (CI: 0.39-0.51) | 0.45 (CI: 0.39-0.52) | 0.17 (CI: 0.10-0.23) | 0.25 (CI: 0.19-0.32) |

**Table S4R**: MCC values with 95% Confidence Intervals (CI) rounded at 2 digits for XGBoost, GRU, Logistic Regression, LightGBM, LSTM, Naïve Bayes models and the clinical model in predicting clinical outcomes of the women population at different visit intervals over a 2-year term using the cumulative approach.

|  | **Prediction at 2 years term** | | | | | | |
| --- | --- | --- | --- | --- | --- | --- | --- |
| **Visit** | **XGboost** | **GRU** | **LogisticRegression** | **LightGBM** | **LSTM** | **Naïve Bayes** | **Clinical model** |
| Baseline | 0.15 (CI: 0.09-0.21) | 0.21 (CI: 0.15-0.27) | 0.20 (CI: 0.14-0.26) | 0.16 (CI: 0.10-0.22) | **0.24 (CI: 0.17-0.30)** | -0.05 (CI: -0.11-0.00) | -0.05 (CI: -0.07--0.03) |
| 1-year | **0.52 (CI: 0.47-0.57)** | 0.40 (CI: 0.34-0.45) | 0.38 (CI: 0.33-0.44) | 0.52 (CI: 0.47-0.56) | 0.41 (CI: 0.36-0.46) | 0.20 (CI: 0.14-0.25) | 0.35 (CI: 0.30-0.40) |
| 2-year | **0.59 (CI: 0.54-0.64)** | 0.50 (CI: 0.44-0.56) | 0.44 (CI: 0.38-0.50) | 0.56 (CI: 0.51-0.61) | 0.50 (CI: 0.44-0.56) | 0.10 (CI: 0.04-0.16) | 0.42 (CI: 0.37-0.48) |
| 3-year | 0.57 (CI: 0.52-0.63) | 0.50 (CI: 0.45-0.56) | 0.50 (CI: 0.43-0.56) | **0.58 (CI: 0.52-0.64)** | 0.52 (CI: 0.46-0.58) | -0.02 (CI: -0.08-0.05) | 0.37 (CI: 0.31-0.43) |
| 4-year | **0.63 (CI: 0.56-0.70)** | 0.49 (CI: 0.42-0.56) | 0.33 (CI: 0.26-0.41) | 0.60 (CI: 0.53-0.68) | 0.49 (CI: 0.42-0.57) | 0.10 (CI: 0.02-0.18) | 0.32 (CI: 0.25-0.39) |
| 5-year | 0.39 (CI: 0.32-0.47) | **0.55 (CI: 0.47-0.62)** | 0.44 (CI: 0.36-0.53) | 0.41 (CI: 0.32-0.50) | 0.54 (CI: 0.46-0.62) | 0.06 (CI: -0.02-0.14) | 0.23 (CI: 0.16-0.30) |
| Average | **0.48 (CI: 0.42-0.54)** | 0.44 (CI: 0.38-0.50) | 0.38 (CI: 0.32-0.45) | 0.47 (CI: 0.41-0.54) | 0.45 (CI: 0.39-0.52) | 0.07 (CI: 0.02-0.11) | 0.27 (CI: 0.23-0.34) |

**Table S4S**: MCC values with 95% Confidence Intervals (CI) rounded at 2 digits for XGBoost, GRU, Logistic Regression, LightGBM, LSTM, Naïve Bayes models and the clinical model in predicting clinical outcomes of the bicuspid population at different visit intervals over a 5-year term using the non-cumulative approach.

|  | **Prediction at 5 years term** | | | | | | |
| --- | --- | --- | --- | --- | --- | --- | --- |
| **Visit** | **XGboost** | **GRU** | **LogisticRegression** | **LightGBM** | **LSTM** | **Naïve Bayes** | **Clinical model** |
| Baseline | **0.50 (CI: 0.47-0.53)** | 0.32 (CI: 0.29-0.35) | 0.33 (CI: 0.30-0.36) | 0.49 (CI: 0.46-0.52) | 0.32 (CI: 0.28-0.35) | 0.02 (CI: -0.00-0.05) | 0.27 (CI: 0.24-0.30) |
| 1-year | **0.54 (CI: 0.52-0.57)** | 0.52 (CI: 0.49-0.55) | 0.42 (CI: 0.39-0.46) | 0.54 (CI: 0.51-0.57) | 0.49 (CI: 0.46-0.52) | 0.11 (CI: 0.08-0.14) | 0.36 (CI: 0.33-0.39) |
| 2-year | **0.51 (CI: 0.48-0.54)** | 0.45 (CI: 0.42-0.48) | 0.42 (CI: 0.39-0.46) | **0.51 (CI: 0.48-0.54)** | 0.43 (CI: 0.40-0.46) | 0.07 (CI: 0.04-0.11) | 0.37 (CI: 0.33-0.41) |
| 3-year | 0.55 (CI: 0.51-0.59) | 0.46 (CI: 0.42-0.50) | 0.41 (CI: 0.37-0.45) | **0.56 (CI: 0.52-0.60)** | 0.45 (CI: 0.42-0.49) | 0.11 (CI: 0.07-0.16) | 0.18 (CI: 0.13-0.23) |
| 4-year | **0.43 (CI: 0.39-0.48)** | 0.37 (CI: 0.32-0.42) | 0.36 (CI: 0.30-0.41) | 0.41 (CI: 0.36-0.46) | 0.36 (CI: 0.31-0.41) | 0.11 (CI: 0.07-0.15) | 0.30 (CI: 0.24-0.36) |
| 5-year | 0.45 (CI: 0.39-0.52) | 0.50 (CI: 0.44-0.56) | 0.45 (CI: 0.39-0.52) | **0.47 (CI: 0.41-0.53)** | 0.48 (CI: 0.42-0.54) | -0.00 (CI: -0.05-0.05) | 0.31 (CI: 0.24-0.37) |
| Average | **0.50 (CI: 0.46-0.54)** | 0.44 (CI: 0.40-0.48) | 0.40 (CI: 0.36-0.44) | **0.50 (CI: 0.46-0.54)** | 0.42 (CI: 0.38-0.46) | 0.07 (CI: 0.04-0.09) | 0.30 (CI: 0.25-0.34) |

**Table S4T**: MCC values with 95% Confidence Intervals (CI) rounded at 2 digits for XGBoost, GRU, Logistic Regression, LightGBM, LSTM, Naïve Bayes models and the clinical model in predicting clinical outcomes of the bicuspid population at different visit intervals over a 2-year term using the non-cumulative approach.

|  | **Prediction at 2 years term** | | | | | | |
| --- | --- | --- | --- | --- | --- | --- | --- |
| **Visit** | **XGboost** | **GRU** | **LogisticRegression** | **LightGBM** | **LSTM** | **Naïve Bayes** | **Clinical model** |
| Baseline | 0.37 (CI: 0.34-0.40) | 0.30 (CI: 0.26-0.33) | 0.28 (CI: 0.26-0.31) | **0.40 (CI: 0.36-0.43)** | 0.32 (CI: 0.29-0.36) | -0.03 (CI: -0.05--0.01) | 0.25 (CI: 0.21-0.28) |
| 1-year | 0.56 (CI: 0.54-0.59) | 0.48 (CI: 0.44-0.52) | 0.45 (CI: 0.42-0.49) | **0.59 (CI: 0.56-0.62)** | 0.48 (CI: 0.45-0.52) | 0.16 (CI: 0.13-0.19) | 0.36 (CI: 0.32-0.39) |
| 2-year | **0.60 (CI: 0.57-0.63)** | 0.58 (CI: 0.56-0.61) | 0.47 (CI: 0.44-0.50) | 0.59 (CI: 0.56-0.62) | 0.59 (CI: 0.57-0.62) | 0.12 (CI: 0.08-0.15) | 0.38 (CI: 0.35-0.41) |
| 3-year | 0.66 (CI: 0.63-0.69) | 0.65 (CI: 0.61-0.68) | 0.61 (CI: 0.58-0.65) | **0.66 (CI: 0.63-0.70)** | 0.63 (CI: 0.59-0.67) | 0.12 (CI: 0.07-0.17) | 0.35 (CI: 0.30-0.39) |
| 4-year | **0.67 (CI: 0.64-0.71)** | 0.62 (CI: 0.58-0.66) | 0.48 (CI: 0.43-0.53) | 0.67 (CI: 0.63-0.71) | 0.64 (CI: 0.60-0.69) | 0.12 (CI: 0.08-0.16) | 0.51 (CI: 0.46-0.57) |
| 5-year | 0.50 (CI: 0.45-0.56) | 0.55 (CI: 0.50-0.60) | 0.37 (CI: 0.32-0.43) | 0.51 (CI: 0.46-0.57) | **0.57 (CI: 0.51-0.62)** | -0.01 (CI: -0.06-0.04) | 0.38 (CI: 0.31-0.46) |
| Average | 0.56 (CI: 0.53-0.60) | 0.53 (CI: 0.49-0.57) | 0.44 (CI: 0.41-0.49) | **0.57 (CI: 0.53-0.61)** | 0.54 (CI: 0.50-0.58) | 0.08 (CI: 0.06-0.12) | 0.37 (CI: 0.33-0.42) |

**Table S4U**: MCC values with 95% Confidence Intervals (CI) rounded at 2 digits for XGBoost, GRU, Logistic Regression, LightGBM, LSTM, Naïve Bayes models and the clinical model in predicting clinical outcomes of the tricuspid population at different visit intervals over a 5-year term using the non-cumulative approach.

|  | **Prediction at 5 years term** | | | | | | |
| --- | --- | --- | --- | --- | --- | --- | --- |
| **Visit** | **XGboost** | **GRU** | **LogisticRegression** | **LightGBM** | **LSTM** | **Naïve Bayes** | **Clinical model** |
| Baseline | **0.43 (CI: 0.37-0.49)** | 0.36 (CI: 0.30-0.42) | 0.38 (CI: 0.32-0.44) | 0.41 (CI: 0.35-0.47) | 0.31 (CI: 0.25-0.38) | 0.03 (CI: -0.00-0.05) | 0.39 (CI: 0.33-0.45) |
| 1-year | 0.50 (CI: 0.45-0.55) | **0.53 (CI: 0.47-0.59)** | 0.50 (CI: 0.45-0.55) | 0.51 (CI: 0.46-0.56) | 0.50 (CI: 0.45-0.56) | 0.23 (CI: 0.18-0.27) | 0.16 (CI: 0.10-0.22) |
| 2-year | 0.62 (CI: 0.57-0.68) | **0.63 (CI: 0.57-0.68)** | 0.58 (CI: 0.52-0.64) | 0.60 (CI: 0.54-0.66) | 0.61 (CI: 0.56-0.67) | 0.11 (CI: 0.06-0.15) | 0.42 (CI: 0.35-0.48) |
| 3-year | 0.55 (CI: 0.47-0.62) | **0.57 (CI: 0.50-0.64)** | 0.49 (CI: 0.42-0.57) | 0.54 (CI: 0.46-0.62) | 0.57 (CI: 0.49-0.65) | 0.06 (CI: 0.01-0.10) | 0.19 (CI: 0.13-0.26) |
| 4-year | **0.44 (CI: 0.35-0.53)** | 0.40 (CI: 0.32-0.49) | 0.33 (CI: 0.25-0.41) | 0.43 (CI: 0.34-0.52) | 0.35 (CI: 0.26-0.43) | 0.01 (CI: -0.01-0.02) | 0.20 (CI: 0.11-0.29) |
| 5-year | 0.37 (CI: 0.28-0.46) | **0.41 (CI: 0.32-0.50)** | 0.35 (CI: 0.26-0.44) | 0.38 (CI: 0.28-0.47) | 0.41 (CI: 0.32-0.49) | 0.03 (CI: 0.00-0.06) | 0.22 (CI: 0.14-0.30) |
| Average | **0.49 (CI: 0.42-0.56)** | 0.48 (CI: 0.41-0.55) | 0.44 (CI: 0.37-0.51) | 0.48 (CI: 0.41-0.55) | 0.46 (CI: 0.39-0.53) | 0.08 (CI: 0.04-0.10) | 0.26 (CI: 0.19-0.33) |

**Table S4V**: MCC values with 95% Confidence Intervals (CI) rounded at 2 digits for XGBoost, GRU, Logistic Regression, LightGBM, LSTM, Naïve Bayes models and the clinical model in predicting clinical outcomes of the tricuspid population at different visit intervals over a 2-year term using the non-cumulative approach.

|  | **Prediction at 2 years term** | | | | | | |
| --- | --- | --- | --- | --- | --- | --- | --- |
| **Visit** | **XGboost** | **GRU** | **LogisticRegression** | **LightGBM** | **LSTM** | **Naïve Bayes** | **Clinical model** |
| Baseline | 0.12 (CI: 0.06-0.18) | 0.11 (CI: 0.05-0.16) | **0.16 (CI: 0.10-0.23)** | 0.08 (CI: 0.01-0.14) | 0.07 (CI: 0.01-0.13) | 0.02 (CI: -0.01-0.04) | 0.10 (CI: 0.03-0.16) |
| 1-year | 0.37 (CI: 0.31-0.42) | 0.35 (CI: 0.29-0.41) | 0.36 (CI: 0.30-0.42) | **0.37 (CI: 0.32-0.43)** | 0.35 (CI: 0.29-0.41) | 0.16 (CI: 0.10-0.21) | 0.11 (CI: 0.05-0.17) |
| 2-year | 0.46 (CI: 0.39-0.52) | 0.49 (CI: 0.43-0.56) | **0.47 (CI: 0.41-0.53)** | 0.43 (CI: 0.37-0.50) | 0.45 (CI: 0.38-0.52) | 0.10 (CI: 0.05-0.14) | 0.42 (CI: 0.35-0.49) |
| 3-year | 0.33 (CI: 0.25-0.40) | **0.42 (CI: 0.34-0.50)** | 0.26 (CI: 0.19-0.33) | 0.34 (CI: 0.27-0.41) | 0.39 (CI: 0.31-0.47) | 0.01 (CI: -0.04-0.05) | 0.30 (CI: 0.21-0.38) |
| 4-year | **0.52 (CI: 0.43-0.61)** | 0.45 (CI: 0.37-0.54) | 0.30 (CI: 0.21-0.40) | 0.52 (CI: 0.43-0.60) | 0.45 (CI: 0.36-0.54) | -0.06 (CI: -0.11--0.01) | 0.36 (CI: 0.27-0.45) |
| 5-year | 0.35 (CI: 0.27-0.44) | 0.38 (CI: 0.30-0.47) | 0.34 (CI: 0.26-0.42) | 0.31 (CI: 0.22-0.39) | **0.39 (CI: 0.31-0.48)** | 0.01 (CI: -0.00-0.01) | 0.28 (CI: 0.19-0.36) |
| Average | 0.36 (CI: 0.29-0.43) | **0.37 (CI: 0.30-0.44)** | 0.32 (CI: 0.25-0.39) | 0.34 (CI: 0.27-0.41) | 0.35 (CI: 0.28-0.43) | 0.04 (CI: 0.01-0.08) | 0.26 (CI: 0.18-0.34) |

**Table S4W**: MCC values with 95% Confidence Intervals (CI) rounded at 2 digits for XGBoost, GRU, Logistic Regression, LightGBM, LSTM, Naïve Bayes models and the clinical model in predicting clinical outcomes of the bicuspid population at different visit intervals over a 5-year term using the cumulative approach.

|  | **Prediction at 5 years term** | | | | | | |
| --- | --- | --- | --- | --- | --- | --- | --- |
| **Visit** | **XGboost** | **GRU** | **LogisticRegression** | **LightGBM** | **LSTM** | **Naïve Bayes** | **Clinical model** |
| Baseline | 0.22 (CI: 0.19-0.25) | 0.30 (CI: 0.26-0.33) | 0.20 (CI: 0.17-0.24) | 0.22 (CI: 0.19-0.26) | **0.32 (CI: 0.29-0.36)** | 0.01 (CI: -0.02-0.05) | -0.03 (CI: -0.06--0.01) |
| 1-year | 0.56 (CI: 0.53-0.58) | 0.48 (CI: 0.44-0.52) | 0.50 (CI: 0.46-0.54) | **0.57 (CI: 0.54-0.59)** | 0.48 (CI: 0.45-0.52) | 0.09 (CI: 0.05-0.12) | 0.29 (CI: 0.26-0.32) |
| 2-year | **0.61 (CI: 0.58-0.64)** | 0.58 (CI: 0.56-0.61) | 0.56 (CI: 0.53-0.59) | 0.60 (CI: 0.57-0.63) | 0.59 (CI: 0.57-0.62) | 0.09 (CI: 0.06-0.13) | 0.35 (CI: 0.31-0.38) |
| 3-year | 0.65 (CI: 0.62-0.69) | 0.65 (CI: 0.61-0.68) | 0.58 (CI: 0.55-0.62) | **0.66 (CI: 0.63-0.70)** | 0.63 (CI: 0.59-0.67) | 0.14 (CI: 0.09-0.18) | 0.38 (CI: 0.34-0.42) |
| 4-year | **0.68 (CI: 0.64-0.72)** | 0.62 (CI: 0.58-0.66) | 0.57 (CI: 0.53-0.61) | 0.67 (CI: 0.63-0.71) | 0.64 (CI: 0.60-0.69) | 0.07 (CI: 0.01-0.13) | 0.40 (CI: 0.36-0.45) |
| 5-year | 0.40 (CI: 0.34-0.46) | 0.55 (CI: 0.50-0.60) | 0.49 (CI: 0.43-0.55) | 0.44 (CI: 0.37-0.50) | **0.57 (CI: 0.51-0.62)** | -0.06 (CI: -0.13-0.01) | 0.21 (CI: 0.15-0.27) |
| Average | 0.52 (CI: 0.48-0.56) | 0.53 (CI: 0.49-0.57) | 0.48 (CI: 0.45-0.53) | 0.53 (CI: 0.49-0.57) | **0.54 (CI: 0.50-0.58)** | 0.06 (CI: 0.02-0.11) | 0.27 (CI: 0.24-0.31) |

**Table S4X**: MCC values with 95% Confidence Intervals (CI) rounded at 2 digits for XGBoost, GRU, Logistic Regression, LightGBM, LSTM, Naïve Bayes models and the clinical model in predicting clinical outcomes of the bicuspid population at different visit intervals over a 2-year term using the cumulative approach.

|  | **Prediction at 2 years term** | | | | | | |
| --- | --- | --- | --- | --- | --- | --- | --- |
| **Visit** | **XGboost** | **GRU** | **LogisticRegression** | **LightGBM** | **LSTM** | **Naïve Bayes** | **Clinical model** |
| Baseline | **0.50 (CI: 0.47-0.54)** | 0.32 (CI: 0.29-0.35) | 0.32 (CI: 0.29-0.36) | 0.45 (CI: 0.42-0.48) | 0.32 (CI: 0.28-0.35) | 0.14 (CI: 0.11-0.18) | 0.27 (CI: 0.25-0.30) |
| 1-year | **0.56 (CI: 0.53-0.59)** | 0.52 (CI: 0.49-0.55) | 0.46 (CI: 0.43-0.49) | 0.54 (CI: 0.51-0.57) | 0.49 (CI: 0.46-0.52) | 0.13 (CI: 0.09-0.16) | 0.35 (CI: 0.32-0.38) |
| 2-year | **0.55 (CI: 0.52-0.58)** | 0.45 (CI: 0.42-0.48) | 0.43 (CI: 0.40-0.47) | 0.52 (CI: 0.49-0.55) | 0.43 (CI: 0.40-0.46) | 0.22 (CI: 0.17-0.26) | 0.26 (CI: 0.23-0.30) |
| 3-year | 0.51 (CI: 0.47-0.54) | 0.46 (CI: 0.42-0.50) | 0.38 (CI: 0.34-0.42) | **0.52 (CI: 0.48-0.56)** | 0.45 (CI: 0.42-0.49) | 0.22 (CI: 0.18-0.27) | 0.28 (CI: 0.24-0.32) |
| 4-year | 0.35 (CI: 0.30-0.40) | **0.37 (CI: 0.32-0.42)** | 0.34 (CI: 0.29-0.40) | 0.34 (CI: 0.29-0.39) | 0.36 (CI: 0.31-0.41) | 0.10 (CI: 0.05-0.15) | 0.24 (CI: 0.20-0.29) |
| 5-year | 0.54 (CI: 0.48-0.60) | 0.50 (CI: 0.44-0.56) | **0.57 (CI: 0.52-0.63)** | 0.56 (CI: 0.50-0.62) | 0.48 (CI: 0.42-0.54) | -0.11 (CI: -0.18--0.05) | 0.19 (CI: 0.14-0.25) |
| Average | **0.50 (CI: 0.46-0.54)** | 0.44 (CI: 0.40-0.48) | 0.42 (CI: 0.38-0.46) | 0.49 (CI: 0.45-0.53) | 0.42 (CI: 0.38-0.46) | 0.12 (CI: 0.08-0.19) | 0.27 (CI: 0.23-0.31) |

**Table S4Y**: MCC values with 95% Confidence Intervals (CI) rounded at 2 digits for XGBoost, GRU, Logistic Regression, LightGBM, LSTM, Naïve Bayes models and the clinical model in predicting clinical outcomes of the tricuspid population at different visit intervals over a 5-year term using the cumulative approach.

|  | **Prediction at 5 years term** | | | | | | |
| --- | --- | --- | --- | --- | --- | --- | --- |
| **Visit** | **XGboost** | **GRU** | **LogisticRegression** | **LightGBM** | **LSTM** | **Naïve Bayes** | **Clinical model** |
| Baseline | 0.13 (CI: 0.07-0.19) | 0.11 (CI: 0.05-0.16) | **0.14 (CI: 0.08-0.21)** | 0.13 (CI: 0.07-0.19) | 0.07 (CI: 0.01-0.13) | -0.05 (CI: -0.11-0.01) | 0.02 (CI: 0.00-0.04) |
| 1-year | 0.41 (CI: 0.35-0.47) | 0.35 (CI: 0.29-0.41) | **0.43 (CI: 0.37-0.49)** | 0.40 (CI: 0.34-0.46) | 0.35 (CI: 0.29-0.41) | 0.12 (CI: 0.06-0.18) | 0.17 (CI: 0.11-0.23) |
| 2-year | 0.51 (CI: 0.45-0.57) | 0.49 (CI: 0.43-0.56) | **0.52 (CI: 0.46-0.58)** | 0.50 (CI: 0.44-0.56) | 0.45 (CI: 0.38-0.52) | 0.14 (CI: 0.08-0.20) | 0.31 (CI: 0.25-0.37) |
| 3-year | 0.46 (CI: 0.39-0.53) | 0.42 (CI: 0.34-0.50) | **0.48 (CI: 0.41-0.55)** | 0.45 (CI: 0.38-0.52) | 0.39 (CI: 0.31-0.47) | -0.09 (CI: -0.17--0.01) | 0.22 (CI: 0.14-0.30) |
| 4-year | 0.49 (CI: 0.41-0.58) | 0.45 (CI: 0.37-0.54) | 0.39 (CI: 0.30-0.49) | **0.56 (CI: 0.48-0.65)** | 0.45 (CI: 0.36-0.54) | 0.18 (CI: 0.09-0.27) | 0.44 (CI: 0.36-0.52) |
| 5-year | 0.17 (CI: 0.09-0.26) | 0.38 (CI: 0.30-0.47) | 0.27 (CI: 0.19-0.36) | 0.18 (CI: 0.10-0.27) | **0.39 (CI: 0.31-0.48)** | 0.15 (CI: 0.06-0.24) | 0.21 (CI: 0.12-0.29) |
| Average | 0.36 (CI: 0.29-0.43) | 0.37 (CI: 0.30-0.44) | **0.37 (CI: 0.30-0.45)** | 0.37 (CI: 0.30-0.44) | 0.35 (CI: 0.28-0.43) | 0.08 (CI: 0.02-0.17) | 0.23 (CI: 0.16-0.29) |

**Table S4Z**: MCC values with 95% Confidence Intervals (CI) rounded at 2 digits for XGBoost, GRU, Logistic Regression, LightGBM, LSTM, Naïve Bayes models and the clinical model in predicting clinical outcomes of the tricuspid population at different visit intervals over a 2-year term using the cumulative approach.

|  | **Prediction at 2 years term** | | | | | | |
| --- | --- | --- | --- | --- | --- | --- | --- |
| **Visit** | **XGboost** | **GRU** | **LogisticRegression** | **LightGBM** | **LSTM** | **Naïve Bayes** | **Clinical model** |
| Baseline | 0.41 (CI: 0.35-0.47) | 0.36 (CI: 0.30-0.42) | 0.39 (CI: 0.32-0.45) | **0.45 (CI: 0.39-0.51)** | 0.31 (CI: 0.25-0.38) | 0.34 (CI: 0.28-0.40) | 0.34 (CI: 0.28-0.41) |
| 1-year | **0.54 (CI: 0.50-0.59)** | 0.53 (CI: 0.47-0.59) | 0.43 (CI: 0.37-0.49) | 0.53 (CI: 0.49-0.58) | 0.50 (CI: 0.45-0.56) | 0.20 (CI: 0.15-0.26) | 0.20 (CI: 0.13-0.26) |
| 2-year | **0.69 (CI: 0.64-0.75)** | 0.63 (CI: 0.57-0.68) | 0.59 (CI: 0.54-0.64) | 0.69 (CI: 0.63-0.75) | 0.61 (CI: 0.56-0.67) | 0.38 (CI: 0.31-0.45) | 0.36 (CI: 0.29-0.43) |
| 3-year | 0.48 (CI: 0.40-0.56) | **0.57 (CI: 0.50-0.64)** | 0.48 (CI: 0.41-0.55) | 0.49 (CI: 0.40-0.57) | 0.57 (CI: 0.49-0.65) | 0.38 (CI: 0.31-0.45) | 0.40 (CI: 0.33-0.47) |
| 4-year | 0.30 (CI: 0.21-0.40) | 0.40 (CI: 0.32-0.49) | **0.41 (CI: 0.33-0.50)** | 0.27 (CI: 0.17-0.37) | 0.35 (CI: 0.26-0.43) | 0.29 (CI: 0.21-0.37) | 0.30 (CI: 0.21-0.40) |
| 5-year | 0.31 (CI: 0.22-0.40) | **0.41 (CI: 0.32-0.50)** | **0.41 (CI: 0.32-0.50)** | 0.21 (CI: 0.11-0.31) | 0.41 (CI: 0.32-0.49) | 0.28 (CI: 0.18-0.37) | 0.16 (CI: 0.07-0.24) |
| Average | 0.46 (CI: 0.39-0.53) | **0.48 (CI: 0.41-0.55)** | 0.45 (CI: 0.38-0.52) | 0.44 (CI: 0.37-0.52) | 0.46 (CI: 0.39-0.53) | 0.31 (CI: 0.24-0.38) | 0.29 (CI: 0.22-0.37) |

**SENSITIVITY (TRUE POSITIVE RATE) RESULTS**

**Table S4alpha**: Sensitivity values with 95% Confidence Intervals (CI) rounded at 2 digits for XGBoost, GRU, Logistic Regression, LightGBM, LSTM, Naïve Bayes models and the clinical model in predicting clinical outcomes at different visit intervals over a 5-year term using the non-cumulative approach. This table represent the sensitivity values of the AUC values presented in table 2A in the main manuscript and has been called alpha to maintain the A-Z hierarchy of the of other tables S4.

|  | **Prediction at 5 years term** | | | | | | |
| --- | --- | --- | --- | --- | --- | --- | --- |
| **Visit** | **XGboost** | **GRU** | **LogisticRegression** | **LightGBM** | **LSTM** | **Naïve Bayes** | **Clinical model** |
| Baseline | **0.77 (CI: 0.75-0.78)** | 0.66 (CI: 0.63-0.68) | 0.70 (CI: 0.68-0.72) | 0.76 (CI: 0.74-0.78) | 0.66 (CI: 0.63-0.68) | 0.04 (CI: 0.02-0.06) | 0.56 (CI: 0.54-0.58) |
| 1-year | 0.80 (CI: 0.78-0.81) | **0.80 (CI: 0.79-0.82)** | 0.75 (CI: 0.73-0.76) | 0.80 (CI: 0.78-0.81) | 0.80 (CI: 0.78-0.82) | 0.14 (CI: 0.11-0.16) | 0.70 (CI: 0.69-0.72) |
| 2-year | **0.80 (CI: 0.78-0.82)** | 0.79 (CI: 0.77-0.81) | 0.74 (CI: 0.72-0.76) | **0.80 (CI: 0.78-0.82)** | **0.80 (CI: 0.78-0.82)** | 0.12 (CI: 0.10-0.14) | 0.78 (CI: 0.77-0.80) |
| 3-year | 0.80 (CI: 0.78-0.82) | 0.78 (CI: 0.76-0.81) | 0.74 (CI: 0.72-0.76) | **0.81 (CI: 0.79-0.83)** | 0.79 (CI: 0.77-0.82) | 0.15 (CI: 0.13-0.18) | 0.81 (CI: 0.79-0.82) |
| 4-year | 0.74 (CI: 0.72-0.77) | 0.75 (CI: 0.72-0.78) | 0.68 (CI: 0.65-0.71) | 0.74 (CI: 0.71-0.77) | 0.75 (CI: 0.72-0.78) | 0.10 (CI: 0.07-0.12) | **0.87 (CI: 0.86-0.89)** |
| 5-year | 0.68 (CI: 0.65-0.72) | 0.75 (CI: 0.72-0.79) | 0.70 (CI: 0.67-0.73) | 0.70 (CI: 0.67-0.73) | 0.75 (CI: 0.72-0.79) | 0.11 (CI: 0.09-0.14) | **0.90 (CI: 0.89-0.91)** |
| Average | 0.77 (CI: 0.74-0.79) | 0.76 (CI: 0.73-0.78) | 0.72 (CI: 0.70-0.74) | 0.77 (CI: 0.75-0.79) | 0.76 (CI: 0.73-0.79) | 0.11 (CI: 0.09-0.13) | **0.77 (CI: 0.76-0.79)** |

**Table S4beta**: Sensitivity values with 95% Confidence Intervals (CI) rounded at 2 digits for XGBoost, GRU, Logistic Regression, LightGBM, LSTM, Naïve Bayes models and the clinical model in predicting clinical outcomes at different visit intervals over a 2-year term using the non-cumulative approach. This table represent the sensitivity values of the AUC values presented in table 2A in the main manuscript and has been called beta to maintain the A-Z hierarchy of the of other tables S4.

|  | **Prediction at 2 years term** | | | | | | |
| --- | --- | --- | --- | --- | --- | --- | --- |
| **Visit** | **XGboost** | **GRU** | **LogisticRegression** | **LightGBM** | **LSTM** | **Naïve Bayes** | **Clinical model** |
| Baseline | 0.56 (CI: 0.54-0.59) | 0.38 (CI: 0.36-0.40) | 0.55 (CI: 0.53-0.57) | **0.58 (CI: 0.56-0.60)** | 0.41 (CI: 0.38-0.43) | 0.03 (CI: 0.00-0.04) | 0.48 (CI: 0.46-0.50) |
| 1-year | 0.80 (CI: 0.78-0.81) | 0.67 (CI: 0.65-0.69) | 0.71 (CI: 0.69-0.73) | **0.81 (CI: 0.80-0.83)** | 0.69 (CI: 0.67-0.71) | 0.13 (CI: 0.11-0.16) | 0.60 (CI: 0.58-0.62) |
| 2-year | **0.80 (CI: 0.78-0.82)** | 0.77 (CI: 0.75-0.79) | 0.72 (CI: 0.70-0.74) | 0.79 (CI: 0.78-0.81) | 0.78 (CI: 0.76-0.80) | 0.13 (CI: 0.10-0.15) | 0.71 (CI: 0.69-0.73) |
| 3-year | **0.82 (CI: 0.80-0.84)** | 0.80 (CI: 0.78-0.82) | 0.72 (CI: 0.70-0.74) | **0.82 (CI: 0.80-0.84)** | **0.82 (CI: 0.80-0.84)** | 0.13 (CI: 0.11-0.16) | 0.78 (CI: 0.76-0.79) |
| 4-year | 0.83 (CI: 0.81-0.85) | 0.79 (CI: 0.77-0.81) | 0.67 (CI: 0.64-0.70) | 0.83 (CI: 0.81-0.85) | 0.82 (CI: 0.80-0.85) | 0.10 (CI: 0.08-0.12) | **0.89 (CI: 0.88-0.90)** |
| 5-year | 0.73 (CI: 0.69-0.76) | 0.76 (CI: 0.73-0.79) | 0.65 (CI: 0.62-0.68) | 0.73 (CI: 0.70-0.77) | 0.79 (CI: 0.76-0.82) | 0.11 (CI: 0.08-0.14) | **0.90 (CI: 0.89-0.91)** |
| Average | 0.76 (CI: 0.73-0.78) | 0.70 (CI: 0.67-0.72) | 0.67 (CI: 0.65-0.69) | **0.76 (CI: 0.74-0.78)** | 0.72 (CI: 0.70-0.74) | 0.11 (CI: 0.08-0.13) | 0.73 (CI: 0.71-0.74) |

**Table S4A**: Sensitivity values with 95% Confidence Intervals (CI) rounded at 2 digits for XGBoost, GRU, Logistic Regression, LightGBM, LSTM, Naïve Bayes models and the clinical model in predicting clinical outcomes at different visit intervals over a 5-year term using the cumulative approach.

|  | **Prediction at 5 years term** | | | | | | |
| --- | --- | --- | --- | --- | --- | --- | --- |
| **Visit** | **XGboost** | **GRU** | **LogisticRegression** | **LightGBM** | **LSTM** | **Naïve Bayes** | **Clinical model** |
| Baseline | 0.74 (CI: 0.72-0.75) | 0.66 (CI: 0.63-0.68) | 0.64 (CI: 0.62-0.66) | 0.72 (CI: 0.71-0.74) | 0.66 (CI: 0.63-0.68) | **0.85 (CI: 0.82-0.89)** | 0.61 (CI: 0.59-0.64) |
| 1-year | 0.79 (CI: 0.77-0.80) | **0.80 (CI: 0.79-0.82)** | 0.73 (CI: 0.71-0.75) | 0.78 (CI: 0.76-0.80) | 0.80 (CI: 0.78-0.82) | 0.29 (CI: 0.23-0.34) | 0.70 (CI: 0.68-0.71) |
| 2-year | **0.80 (CI: 0.78-0.82)** | 0.79 (CI: 0.77-0.81) | 0.76 (CI: 0.74-0.79) | **0.80 (CI: 0.78-0.82)** | **0.80 (CI: 0.78-0.82)** | 0.52 (CI: 0.47-0.58) | 0.65 (CI: 0.63-0.68) |
| 3-year | 0.78 (CI: 0.76-0.81) | 0.78 (CI: 0.76-0.81) | 0.75 (CI: 0.73-0.77) | 0.79 (CI: 0.76-0.81) | **0.79 (CI: 0.77-0.82)** | 0.78 (CI: 0.75-0.80) | 0.70 (CI: 0.67-0.72) |
| 4-year | 0.76 (CI: 0.73-0.79) | 0.75 (CI: 0.72-0.78) | 0.78 (CI: 0.75-0.81) | 0.75 (CI: 0.72-0.78) | 0.75 (CI: 0.72-0.78) | 0.76 (CI: 0.73-0.79) | **0.79 (CI: 0.77-0.82)** |
| 5-year | **0.87 (CI: 0.84-0.90)** | 0.75 (CI: 0.72-0.79) | 0.87 (CI: 0.84-0.89) | 0.86 (CI: 0.83-0.89) | 0.75 (CI: 0.72-0.79) | 0.78 (CI: 0.75-0.81) | 0.84 (CI: 0.81-0.87) |
| Average | **0.79 (CI: 0.77-0.81)** | 0.76 (CI: 0.73-0.78) | 0.76 (CI: 0.73-0.78) | 0.78 (CI: 0.76-0.81) | 0.76 (CI: 0.73-0.79) | 0.66 (CI: 0.63-0.70) | 0.72 (CI: 0.69-0.74) |

**Table S4B**: Sensitivity values with 95% Confidence Intervals (CI) rounded at 2 digits for XGBoost, GRU, Logistic Regression, LightGBM, LSTM, Naïve Bayes models and the clinical model in predicting clinical outcomes at different visit intervals over a 2-year term using the cumulative approach.

|  | **Prediction at 2 years term** | | | | | | |
| --- | --- | --- | --- | --- | --- | --- | --- |
| **Visit** | **XGboost** | **GRU** | **LogisticRegression** | **LightGBM** | **LSTM** | **Naïve Bayes** | **Clinical model** |
| Baseline | 0.37 (CI: 0.34-0.39) | 0.38 (CI: 0.36-0.40) | 0.39 (CI: 0.36-0.41) | 0.36 (CI: 0.33-0.38) | 0.41 (CI: 0.38-0.43) | **0.86 (CI: 0.84-0.88)** | 0.03 (CI: 0.02-0.03) |
| 1-year | **0.76 (CI: 0.75-0.78)** | 0.67 (CI: 0.65-0.69) | 0.72 (CI: 0.70-0.74) | 0.76 (CI: 0.74-0.78) | 0.69 (CI: 0.67-0.71) | 0.15 (CI: 0.12-0.18) | 0.57 (CI: 0.55-0.59) |
| 2-year | **0.81 (CI: 0.79-0.83)** | 0.77 (CI: 0.75-0.79) | 0.78 (CI: 0.76-0.80) | 0.80 (CI: 0.78-0.82) | 0.78 (CI: 0.76-0.80) | 0.19 (CI: 0.16-0.22) | 0.64 (CI: 0.62-0.66) |
| 3-year | 0.84 (CI: 0.82-0.86) | 0.80 (CI: 0.78-0.82) | 0.80 (CI: 0.78-0.82) | **0.85 (CI: 0.84-0.87)** | 0.82 (CI: 0.80-0.84) | 0.31 (CI: 0.28-0.34) | 0.73 (CI: 0.71-0.75) |
| 4-year | **0.86 (CI: 0.84-0.88)** | 0.79 (CI: 0.77-0.81) | 0.80 (CI: 0.78-0.82) | **0.86 (CI: 0.84-0.88)** | 0.82 (CI: 0.80-0.85) | 0.53 (CI: 0.50-0.56) | 0.85 (CI: 0.82-0.87) |
| 5-year | 0.80 (CI: 0.78-0.83) | 0.76 (CI: 0.73-0.79) | 0.81 (CI: 0.78-0.84) | **0.82 (CI: 0.79-0.85)** | 0.79 (CI: 0.76-0.82) | 0.69 (CI: 0.65-0.72) | 0.81 (CI: 0.79-0.84) |
| Average | **0.74 (CI: 0.72-0.76)** | 0.70 (CI: 0.67-0.72) | 0.72 (CI: 0.69-0.74) | **0.74 (CI: 0.72-0.76)** | 0.72 (CI: 0.70-0.74) | 0.46 (CI: 0.43-0.48) | 0.61 (CI: 0.59-0.62) |

**Table S4C**: Sensitivity values with 95% Confidence Intervals (CI) rounded at 2 digits for XGBoost, GRU, Logistic Regression, LightGBM, LSTM, Naïve Bayes models and the clinical model in predicting clinical outcomes of the population strictly above 68 years old (median age of the cohort) at different visit intervals over a 5-year term using the non-cumulative approach.

|  | **Prediction at 5 years term** | | | | | | |
| --- | --- | --- | --- | --- | --- | --- | --- |
| **Visit** | **XGboost** | **GRU** | **LogisticRegression** | **LightGBM** | **LSTM** | **Naïve Bayes** | **Clinical model** |
| Baseline | **0.79 (CI: 0.77-0.82)** | 0.70 (CI: 0.67-0.73) | 0.70 (CI: 0.67-0.73) | 0.78 (CI: 0.76-0.81) | 0.70 (CI: 0.67-0.73) | 0.05 (CI: 0.02-0.07) | 0.57 (CI: 0.54-0.59) |
| 1-year | 0.83 (CI: 0.81-0.85) | **0.84 (CI: 0.82-0.86)** | 0.77 (CI: 0.75-0.79) | 0.83 (CI: 0.81-0.85) | **0.84 (CI: 0.82-0.86)** | 0.15 (CI: 0.12-0.17) | 0.74 (CI: 0.71-0.76) |
| 2-year | 0.83 (CI: 0.81-0.86) | 0.82 (CI: 0.80-0.85) | 0.77 (CI: 0.75-0.80) | **0.84 (CI: 0.81-0.86)** | 0.82 (CI: 0.80-0.85) | 0.16 (CI: 0.13-0.18) | 0.78 (CI: 0.75-0.80) |
| 3-year | 0.84 (CI: 0.82-0.87) | 0.83 (CI: 0.80-0.85) | 0.77 (CI: 0.74-0.80) | **0.86 (CI: 0.84-0.89)** | 0.83 (CI: 0.81-0.86) | 0.20 (CI: 0.17-0.23) | 0.81 (CI: 0.78-0.83) |
| 4-year | 0.76 (CI: 0.72-0.80) | 0.76 (CI: 0.72-0.80) | 0.68 (CI: 0.64-0.73) | 0.77 (CI: 0.72-0.81) | 0.76 (CI: 0.72-0.80) | 0.13 (CI: 0.10-0.16) | **0.90 (CI: 0.87-0.92)** |
| 5-year | 0.75 (CI: 0.70-0.79) | 0.79 (CI: 0.75-0.84) | 0.77 (CI: 0.73-0.81) | 0.77 (CI: 0.72-0.81) | 0.78 (CI: 0.73-0.83) | 0.18 (CI: 0.14-0.22) | **0.92 (CI: 0.90-0.94)** |
| Average | 0.80 (CI: 0.77-0.83) | 0.79 (CI: 0.76-0.82) | 0.74 (CI: 0.71-0.78) | **0.81 (CI: 0.78-0.84)** | 0.79 (CI: 0.76-0.82) | 0.15 (CI: 0.11-0.17) | 0.79 (CI: 0.76-0.81) |

**Table S4D**: Sensitivity values with 95% Confidence Intervals (CI) rounded at 2 digits for XGBoost, GRU, Logistic Regression, LightGBM, LSTM, Naïve Bayes models and the clinical model in predicting clinical outcomes of the population strictly above 68 years old (median age of the cohort) at different visit intervals over a 2-year term using the non-cumulative approach.

|  | **Prediction at 2 years term** | | | | | | |
| --- | --- | --- | --- | --- | --- | --- | --- |
| **Visit** | **XGboost** | **GRU** | **LogisticRegression** | **LightGBM** | **LSTM** | **Naïve Bayes** | **Clinical model** |
| Baseline | 0.59 (CI: 0.56-0.63) | 0.45 (CI: 0.41-0.48) | 0.55 (CI: 0.51-0.58) | **0.61 (CI: 0.58-0.64)** | 0.48 (CI: 0.44-0.51) | 0.03 (CI: 0.00-0.05) | 0.53 (CI: 0.50-0.57) |
| 1-year | 0.81 (CI: 0.79-0.83) | 0.72 (CI: 0.69-0.75) | 0.70 (CI: 0.68-0.73) | **0.84 (CI: 0.82-0.86)** | 0.72 (CI: 0.69-0.75) | 0.13 (CI: 0.10-0.15) | 0.66 (CI: 0.63-0.69) |
| 2-year | **0.84 (CI: 0.82-0.87)** | 0.80 (CI: 0.77-0.82) | 0.71 (CI: 0.68-0.74) | **0.84 (CI: 0.82-0.87)** | 0.81 (CI: 0.79-0.84) | 0.16 (CI: 0.13-0.19) | 0.71 (CI: 0.68-0.74) |
| 3-year | 0.86 (CI: 0.83-0.88) | 0.84 (CI: 0.82-0.87) | 0.72 (CI: 0.69-0.76) | **0.86 (CI: 0.84-0.89)** | 0.85 (CI: 0.83-0.88) | 0.18 (CI: 0.15-0.21) | 0.79 (CI: 0.77-0.82) |
| 4-year | 0.83 (CI: 0.80-0.87) | 0.78 (CI: 0.74-0.82) | 0.65 (CI: 0.61-0.69) | 0.82 (CI: 0.79-0.86) | 0.82 (CI: 0.78-0.85) | 0.12 (CI: 0.09-0.15) | **0.91 (CI: 0.89-0.93)** |
| 5-year | 0.78 (CI: 0.74-0.83) | 0.79 (CI: 0.75-0.84) | 0.67 (CI: 0.62-0.71) | 0.78 (CI: 0.74-0.83) | 0.81 (CI: 0.77-0.86) | 0.18 (CI: 0.13-0.21) | **0.90 (CI: 0.88-0.93)** |
| Average | 0.79 (CI: 0.76-0.82) | 0.73 (CI: 0.70-0.76) | 0.67 (CI: 0.63-0.70) | **0.79 (CI: 0.77-0.83)** | 0.75 (CI: 0.72-0.78) | 0.13 (CI: 0.10-0.16) | 0.75 (CI: 0.73-0.78) |

**Table S4E**: Sensitivity values with 95% Confidence Intervals (CI) rounded at 2 digits for XGBoost, GRU, Logistic Regression, LightGBM, LSTM, Naïve Bayes models and the clinical model in predicting clinical outcomes of the population under or equal to 68 years old (median age of the cohort) at different visit intervals over a 5-year term using the non-cumulative approach.

|  | **Prediction at 5 years term** | | | | | | |
| --- | --- | --- | --- | --- | --- | --- | --- |
| **Visit** | **XGboost** | **GRU** | **LogisticRegression** | **LightGBM** | **LSTM** | **Naïve Bayes** | **Clinical model** |
| Baseline | **0.74 (CI: 0.71-0.77)** | 0.62 (CI: 0.59-0.65) | 0.69 (CI: 0.66-0.72) | 0.73 (CI: 0.70-0.75) | 0.62 (CI: 0.59-0.65) | 0.03 (CI: 0.01-0.05) | 0.55 (CI: 0.52-0.58) |
| 1-year | 0.75 (CI: 0.73-0.78) | 0.76 (CI: 0.73-0.79) | 0.72 (CI: 0.69-0.74) | **0.76 (CI: 0.74-0.79)** | 0.76 (CI: 0.73-0.79) | 0.13 (CI: 0.10-0.15) | 0.66 (CI: 0.63-0.69) |
| 2-year | 0.76 (CI: 0.73-0.79) | 0.75 (CI: 0.72-0.78) | 0.70 (CI: 0.67-0.73) | 0.75 (CI: 0.72-0.78) | 0.76 (CI: 0.73-0.79) | 0.09 (CI: 0.06-0.11) | **0.78 (CI: 0.76-0.80)** |
| 3-year | 0.75 (CI: 0.72-0.78) | 0.72 (CI: 0.69-0.76) | 0.71 (CI: 0.67-0.74) | 0.75 (CI: 0.71-0.79) | 0.74 (CI: 0.70-0.78) | 0.09 (CI: 0.06-0.12) | **0.81 (CI: 0.78-0.84)** |
| 4-year | 0.74 (CI: 0.70-0.78) | 0.73 (CI: 0.68-0.78) | 0.68 (CI: 0.63-0.72) | 0.72 (CI: 0.67-0.76) | 0.73 (CI: 0.69-0.78) | 0.06 (CI: 0.03-0.08) | **0.91 (CI: 0.89-0.93)** |
| 5-year | 0.61 (CI: 0.55-0.67) | 0.71 (CI: 0.66-0.76) | 0.62 (CI: 0.56-0.67) | 0.63 (CI: 0.57-0.68) | 0.72 (CI: 0.67-0.78) | 0.05 (CI: 0.02-0.07) | **0.94 (CI: 0.92-0.96)** |
| Average | 0.73 (CI: 0.69-0.76) | 0.72 (CI: 0.68-0.75) | 0.69 (CI: 0.65-0.72) | 0.72 (CI: 0.69-0.76) | 0.72 (CI: 0.69-0.76) | 0.08 (CI: 0.05-0.10) | **0.78 (CI: 0.75-0.80)** |

**Table S4F**: Sensitivity values with 95% Confidence Intervals (CI) rounded at 2 digits for XGBoost, GRU, Logistic Regression, LightGBM, LSTM, Naïve Bayes models and the clinical model in predicting clinical outcomes of the population under or equal to 68 years old (median age of the cohort) at different visit intervals over a 2-year term using the non-cumulative approach.

|  | **Prediction at 2 years term** | | | | | | |
| --- | --- | --- | --- | --- | --- | --- | --- |
| **Visit** | **XGboost** | **GRU** | **LogisticRegression** | **LightGBM** | **LSTM** | **Naïve Bayes** | **Clinical model** |
| Baseline | 0.55 (CI: 0.52-0.59) | 0.33 (CI: 0.29-0.36) | 0.55 (CI: 0.51-0.58) | **0.56 (CI: 0.52-0.60)** | 0.34 (CI: 0.31-0.38) | 0.02 (CI: -0.00-0.04) | 0.44 (CI: 0.40-0.48) |
| 1-year | **0.78 (CI: 0.76-0.81)** | 0.62 (CI: 0.59-0.65) | 0.72 (CI: 0.69-0.75) | **0.78 (CI: 0.76-0.81)** | 0.65 (CI: 0.62-0.68) | 0.14 (CI: 0.11-0.17) | 0.53 (CI: 0.50-0.56) |
| 2-year | **0.76 (CI: 0.74-0.79)** | 0.73 (CI: 0.70-0.77) | 0.71 (CI: 0.68-0.74) | 0.75 (CI: 0.72-0.77) | 0.75 (CI: 0.72-0.78) | 0.10 (CI: 0.07-0.13) | 0.73 (CI: 0.71-0.76) |
| 3-year | 0.79 (CI: 0.76-0.82) | 0.76 (CI: 0.73-0.79) | 0.71 (CI: 0.68-0.74) | 0.79 (CI: 0.76-0.82) | 0.78 (CI: 0.75-0.81) | 0.09 (CI: 0.06-0.11) | **0.80 (CI: 0.77-0.82)** |
| 4-year | 0.83 (CI: 0.80-0.86) | 0.80 (CI: 0.76-0.83) | 0.70 (CI: 0.66-0.74) | 0.84 (CI: 0.81-0.88) | 0.83 (CI: 0.80-0.86) | 0.08 (CI: 0.05-0.10) | **0.93 (CI: 0.91-0.95)** |
| 5-year | 0.68 (CI: 0.63-0.74) | 0.74 (CI: 0.69-0.79) | 0.66 (CI: 0.61-0.72) | 0.68 (CI: 0.63-0.74) | 0.78 (CI: 0.73-0.82) | 0.05 (CI: 0.02-0.07) | **0.92 (CI: 0.90-0.95)** |
| Average | **0.73 (CI: 0.70-0.77)** | 0.66 (CI: 0.63-0.70) | 0.68 (CI: 0.64-0.71) | **0.73 (CI: 0.70-0.77)** | 0.69 (CI: 0.66-0.72) | 0.08 (CI: 0.05-0.10) | 0.73 (CI: 0.70-0.75) |

**Table S4G**: Sensitivity values with 95% Confidence Intervals (CI) rounded at 2 digits for XGBoost, GRU, Logistic Regression, LightGBM, LSTM, Naïve Bayes models and the clinical model in predicting clinical outcomes of the population strictly above 68 years old (median age of the cohort) at different visit intervals over a 5-year term using the cumulative approach.

|  | **Prediction at 5 years term** | | | | | | |
| --- | --- | --- | --- | --- | --- | --- | --- |
| **Visit** | **XGboost** | **GRU** | **LogisticRegression** | **LightGBM** | **LSTM** | **Naïve Bayes** | **Clinical model** |
| Baseline | 0.77 (CI: 0.75-0.79) | 0.70 (CI: 0.67-0.73) | 0.66 (CI: 0.64-0.69) | 0.74 (CI: 0.72-0.76) | 0.70 (CI: 0.67-0.73) | **0.88 (CI: 0.85-0.91)** | 0.61 (CI: 0.59-0.64) |
| 1-year | **0.81 (CI: 0.79-0.83)** | 0.84 (CI: 0.82-0.86) | 0.75 (CI: 0.73-0.78) | **0.81 (CI: 0.79-0.83)** | 0.84 (CI: 0.82-0.86) | 0.32 (CI: 0.26-0.38) | 0.72 (CI: 0.70-0.74) |
| 2-year | **0.81 (CI: 0.79-0.84)** | 0.82 (CI: 0.80-0.85) | 0.77 (CI: 0.74-0.80) | 0.81 (CI: 0.79-0.83) | 0.82 (CI: 0.80-0.85) | 0.55 (CI: 0.49-0.61) | 0.70 (CI: 0.67-0.73) |
| 3-year | 0.82 (CI: 0.79-0.85) | 0.83 (CI: 0.80-0.85) | 0.76 (CI: 0.73-0.80) | 0.82 (CI: 0.79-0.85) | **0.83 (CI: 0.81-0.86)** | 0.78 (CI: 0.75-0.81) | 0.71 (CI: 0.67-0.75) |
| 4-year | **0.79 (CI: 0.75-0.82)** | 0.76 (CI: 0.72-0.80) | 0.77 (CI: 0.74-0.81) | 0.75 (CI: 0.72-0.79) | 0.76 (CI: 0.72-0.80) | 0.75 (CI: 0.72-0.78) | 0.78 (CI: 0.74-0.81) |
| 5-year | **0.90 (CI: 0.87-0.93)** | 0.79 (CI: 0.75-0.84) | 0.87 (CI: 0.84-0.90) | 0.89 (CI: 0.87-0.93) | 0.78 (CI: 0.73-0.83) | 0.74 (CI: 0.70-0.78) | 0.87 (CI: 0.84-0.91) |
| Average | **0.82 (CI: 0.79-0.84)** | 0.79 (CI: 0.76-0.82) | 0.76 (CI: 0.74-0.80) | 0.80 (CI: 0.78-0.83) | 0.79 (CI: 0.76-0.82) | 0.67 (CI: 0.63-0.71) | 0.73 (CI: 0.70-0.76) |

**Table S4H**: Sensitivity values with 95% Confidence Intervals (CI) rounded at 2 digits for XGBoost, GRU, Logistic Regression, LightGBM, LSTM, Naïve Bayes models and the clinical model in predicting clinical outcomes of the population strictly above 68 years old (median age of the cohort) at different visit intervals over a 2-year term using the cumulative approach.

|  | **Prediction at 2 years term** | | | | | | |
| --- | --- | --- | --- | --- | --- | --- | --- |
| **Visit** | **XGboost** | **GRU** | **LogisticRegression** | **LightGBM** | **LSTM** | **Naïve Bayes** | **Clinical model** |
| Baseline | 0.35 (CI: 0.32-0.39) | 0.45 (CI: 0.41-0.48) | 0.36 (CI: 0.33-0.40) | 0.36 (CI: 0.32-0.39) | 0.48 (CI: 0.44-0.51) | **0.85 (CI: 0.82-0.88)** | 0.05 (CI: 0.03-0.07) |
| 1-year | **0.79 (CI: 0.77-0.82)** | 0.72 (CI: 0.69-0.75) | 0.75 (CI: 0.73-0.78) | **0.79 (CI: 0.77-0.82)** | 0.72 (CI: 0.69-0.75) | 0.17 (CI: 0.14-0.20) | 0.62 (CI: 0.60-0.65) |
| 2-year | **0.82 (CI: 0.79-0.84)** | 0.80 (CI: 0.77-0.82) | 0.81 (CI: 0.78-0.83) | 0.81 (CI: 0.79-0.83) | 0.81 (CI: 0.79-0.84) | 0.18 (CI: 0.15-0.21) | 0.69 (CI: 0.66-0.72) |
| 3-year | 0.86 (CI: 0.83-0.88) | 0.84 (CI: 0.82-0.87) | 0.80 (CI: 0.76-0.83) | **0.86 (CI: 0.84-0.88)** | 0.85 (CI: 0.83-0.88) | 0.34 (CI: 0.30-0.38) | 0.78 (CI: 0.75-0.81) |
| 4-year | 0.83 (CI: 0.80-0.86) | 0.78 (CI: 0.74-0.82) | 0.80 (CI: 0.77-0.84) | **0.83 (CI: 0.80-0.87)** | 0.82 (CI: 0.78-0.85) | 0.52 (CI: 0.48-0.56) | 0.80 (CI: 0.76-0.84) |
| 5-year | 0.85 (CI: 0.82-0.89) | 0.79 (CI: 0.75-0.84) | 0.84 (CI: 0.80-0.88) | **0.86 (CI: 0.82-0.89)** | 0.81 (CI: 0.77-0.86) | 0.67 (CI: 0.62-0.72) | 0.84 (CI: 0.80-0.88) |
| Average | **0.75 (CI: 0.72-0.78)** | 0.73 (CI: 0.70-0.76) | 0.73 (CI: 0.70-0.76) | **0.75 (CI: 0.72-0.78)** | **0.75 (CI: 0.72-0.78)** | 0.46 (CI: 0.42-0.49) | 0.63 (CI: 0.60-0.66) |

**Table S4I**: Sensitivity values with 95% Confidence Intervals (CI) rounded at 2 digits for XGBoost, GRU, Logistic Regression, LightGBM, LSTM, Naïve Bayes models and the clinical model in predicting clinical outcomes of the population under or equal to 68 years old (median age of the cohort) at different visit intervals over a 5-year term using the cumulative approach.

|  | **Prediction at 5 years term** | | | | | | |
| --- | --- | --- | --- | --- | --- | --- | --- |
| **Visit** | **XGboost** | **GRU** | **LogisticRegression** | **LightGBM** | **LSTM** | **Naïve Bayes** | **Clinical model** |
| Baseline | 0.70 (CI: 0.68-0.73) | 0.62 (CI: 0.59-0.65) | 0.63 (CI: 0.59-0.66) | 0.70 (CI: 0.68-0.73) | 0.62 (CI: 0.59-0.65) | **0.83 (CI: 0.79-0.87)** | 0.61 (CI: 0.58-0.65) |
| 1-year | 0.76 (CI: 0.73-0.78) | **0.76 (CI: 0.73-0.79)** | 0.71 (CI: 0.68-0.74) | 0.75 (CI: 0.73-0.78) | **0.76 (CI: 0.73-0.79)** | 0.25 (CI: 0.19-0.30) | 0.66 (CI: 0.64-0.69) |
| 2-year | 0.79 (CI: 0.76-0.82) | 0.75 (CI: 0.72-0.78) | 0.76 (CI: 0.73-0.79) | **0.80 (CI: 0.77-0.82)** | 0.76 (CI: 0.73-0.79) | 0.49 (CI: 0.43-0.55) | 0.59 (CI: 0.56-0.62) |
| 3-year | 0.74 (CI: 0.70-0.78) | 0.72 (CI: 0.69-0.76) | 0.73 (CI: 0.69-0.76) | **0.75 (CI: 0.71-0.79)** | 0.74 (CI: 0.70-0.78) | **0.75 (CI: 0.71-0.79)** | 0.69 (CI: 0.65-0.72) |
| 4-year | 0.75 (CI: 0.71-0.79) | 0.73 (CI: 0.68-0.78) | 0.79 (CI: 0.75-0.83) | 0.75 (CI: 0.71-0.79) | 0.73 (CI: 0.69-0.78) | 0.77 (CI: 0.73-0.82) | **0.82 (CI: 0.78-0.85)** |
| 5-year | 0.82 (CI: 0.78-0.87) | 0.71 (CI: 0.66-0.76) | **0.86 (CI: 0.83-0.90)** | 0.81 (CI: 0.76-0.85) | 0.72 (CI: 0.67-0.78) | 0.81 (CI: 0.76-0.85) | 0.79 (CI: 0.75-0.84) |
| Average | **0.76 (CI: 0.73-0.80)** | 0.72 (CI: 0.68-0.75) | 0.75 (CI: 0.71-0.78) | 0.76 (CI: 0.73-0.79) | 0.72 (CI: 0.69-0.76) | 0.65 (CI: 0.60-0.70) | 0.69 (CI: 0.66-0.73) |

**Table S4J**: Sensitivity values with 95% Confidence Intervals (CI) rounded at 2 digits for XGBoost, GRU, Logistic Regression, LightGBM, LSTM, Naïve Bayes models and the clinical model in predicting clinical outcomes of the population under or equal to 68 years old (median age of the cohort) at different visit intervals over a 2-year term using the cumulative approach.

|  | **Prediction at 2 years term** | | | | | | |
| --- | --- | --- | --- | --- | --- | --- | --- |
| **Visit** | **XGboost** | **GRU** | **LogisticRegression** | **LightGBM** | **LSTM** | **Naïve Bayes** | **Clinical model** |
| Baseline | 0.38 (CI: 0.35-0.42) | 0.33 (CI: 0.29-0.36) | 0.41 (CI: 0.37-0.44) | 0.37 (CI: 0.33-0.40) | 0.34 (CI: 0.31-0.38) | **0.86 (CI: 0.83-0.88)** | 0.00 (CI: -0.00-0.01) |
| 1-year | **0.73 (CI: 0.70-0.76)** | 0.62 (CI: 0.59-0.65) | 0.67 (CI: 0.64-0.70) | 0.72 (CI: 0.69-0.75) | 0.65 (CI: 0.62-0.68) | 0.12 (CI: 0.10-0.15) | 0.51 (CI: 0.48-0.54) |
| 2-year | **0.80 (CI: 0.77-0.83)** | 0.73 (CI: 0.70-0.77) | 0.75 (CI: 0.73-0.78) | 0.79 (CI: 0.76-0.82) | 0.75 (CI: 0.72-0.78) | 0.20 (CI: 0.16-0.23) | 0.57 (CI: 0.54-0.60) |
| 3-year | 0.82 (CI: 0.79-0.85) | 0.76 (CI: 0.73-0.79) | 0.80 (CI: 0.77-0.83) | **0.85 (CI: 0.82-0.88)** | 0.78 (CI: 0.75-0.81) | 0.28 (CI: 0.24-0.32) | 0.68 (CI: 0.65-0.71) |
| 4-year | 0.89 (CI: 0.86-0.91) | 0.80 (CI: 0.76-0.83) | 0.81 (CI: 0.78-0.84) | 0.89 (CI: 0.87-0.92) | 0.83 (CI: 0.80-0.86) | 0.54 (CI: 0.50-0.59) | **0.90 (CI: 0.88-0.93)** |
| 5-year | 0.77 (CI: 0.72-0.81) | 0.74 (CI: 0.69-0.79) | **0.79 (CI: 0.75-0.84)** | **0.79 (CI: 0.74-0.84)** | 0.78 (CI: 0.73-0.82) | 0.71 (CI: 0.65-0.76) | **0.79 (CI: 0.75-0.84)** |
| Average | 0.73 (CI: 0.70-0.76) | 0.66 (CI: 0.63-0.70) | 0.71 (CI: 0.67-0.74) | **0.74 (CI: 0.70-0.77)** | 0.69 (CI: 0.66-0.72) | 0.45 (CI: 0.41-0.49) | 0.58 (CI: 0.55-0.60) |

**Table S4K**: Sensitivity values with 95% Confidence Intervals (CI) rounded at 2 digits for XGBoost, GRU, Logistic Regression, LightGBM, LSTM, Naïve Bayes models and the clinical model in predicting clinical outcomes of the men population at different visit intervals over a 5-year term using the non-cumulative approach.

|  | **Prediction at 5 years term** | | | | | | |
| --- | --- | --- | --- | --- | --- | --- | --- |
| **Visit** | **XGboost** | **GRU** | **LogisticRegression** | **LightGBM** | **LSTM** | **Naïve Bayes** | **Clinical model** |
| Baseline | **0.76 (CI: 0.74-0.78)** | 0.66 (CI: 0.64-0.69) | 0.71 (CI: 0.69-0.73) | **0.76 (CI: 0.74-0.78)** | 0.66 (CI: 0.63-0.68) | 0.05 (CI: 0.02-0.06) | 0.54 (CI: 0.52-0.56) |
| 1-year | 0.81 (CI: 0.79-0.83) | 0.81 (CI: 0.79-0.83) | 0.77 (CI: 0.75-0.79) | **0.81 (CI: 0.80-0.83)** | 0.80 (CI: 0.78-0.82) | 0.14 (CI: 0.11-0.16) | 0.69 (CI: 0.67-0.72) |
| 2-year | 0.82 (CI: 0.80-0.84) | 0.80 (CI: 0.77-0.82) | 0.76 (CI: 0.74-0.79) | **0.83 (CI: 0.81-0.85)** | 0.80 (CI: 0.78-0.83) | 0.13 (CI: 0.10-0.15) | 0.78 (CI: 0.76-0.80) |
| 3-year | 0.81 (CI: 0.79-0.84) | 0.79 (CI: 0.76-0.81) | 0.76 (CI: 0.73-0.79) | **0.83 (CI: 0.80-0.85)** | 0.80 (CI: 0.77-0.82) | 0.15 (CI: 0.12-0.17) | 0.80 (CI: 0.78-0.82) |
| 4-year | 0.73 (CI: 0.70-0.76) | 0.74 (CI: 0.71-0.77) | 0.67 (CI: 0.63-0.70) | 0.72 (CI: 0.69-0.76) | 0.75 (CI: 0.71-0.78) | 0.08 (CI: 0.05-0.10) | **0.90 (CI: 0.88-0.92)** |
| 5-year | 0.62 (CI: 0.58-0.66) | 0.71 (CI: 0.68-0.76) | 0.67 (CI: 0.63-0.71) | 0.63 (CI: 0.59-0.67) | 0.72 (CI: 0.68-0.76) | 0.16 (CI: 0.13-0.19) | **0.93 (CI: 0.91-0.95)** |
| Average | 0.76 (CI: 0.73-0.79) | 0.75 (CI: 0.73-0.78) | 0.72 (CI: 0.70-0.75) | 0.76 (CI: 0.74-0.79) | 0.76 (CI: 0.73-0.78) | 0.12 (CI: 0.09-0.14) | **0.77 (CI: 0.75-0.80)** |

**Table S4L**: Sensitivity values with 95% Confidence Intervals (CI) rounded at 2 digits for XGBoost, GRU, Logistic Regression, LightGBM, LSTM, Naïve Bayes models and the clinical model in predicting clinical outcomes of the men population at different visit intervals over a 2-year term using the non-cumulative approach.

|  | **Prediction at 2 years term** | | | | | | |
| --- | --- | --- | --- | --- | --- | --- | --- |
| **Visit** | **XGboost** | **GRU** | **LogisticRegression** | **LightGBM** | **LSTM** | **Naïve Bayes** | **Clinical model** |
| Baseline | 0.57 (CI: 0.54-0.60) | 0.40 (CI: 0.37-0.43) | 0.56 (CI: 0.54-0.59) | **0.59 (CI: 0.57-0.62)** | 0.42 (CI: 0.39-0.45) | 0.03 (CI: 0.00-0.05) | 0.46 (CI: 0.44-0.48) |
| 1-year | 0.79 (CI: 0.77-0.81) | 0.69 (CI: 0.67-0.71) | 0.72 (CI: 0.70-0.74) | **0.81 (CI: 0.79-0.83)** | 0.70 (CI: 0.68-0.72) | 0.13 (CI: 0.11-0.16) | 0.55 (CI: 0.52-0.58) |
| 2-year | **0.81 (CI: 0.78-0.83)** | 0.78 (CI: 0.76-0.80) | 0.73 (CI: 0.71-0.76) | 0.80 (CI: 0.78-0.82) | 0.79 (CI: 0.77-0.82) | 0.13 (CI: 0.10-0.15) | 0.72 (CI: 0.70-0.74) |
| 3-year | **0.84 (CI: 0.82-0.86)** | 0.82 (CI: 0.80-0.85) | 0.75 (CI: 0.73-0.77) | 0.83 (CI: 0.81-0.86) | **0.84 (CI: 0.82-0.86)** | 0.14 (CI: 0.11-0.16) | 0.79 (CI: 0.77-0.81) |
| 4-year | 0.85 (CI: 0.83-0.88) | 0.81 (CI: 0.79-0.84) | 0.73 (CI: 0.70-0.76) | 0.85 (CI: 0.83-0.88) | 0.84 (CI: 0.82-0.87) | 0.10 (CI: 0.07-0.13) | **0.92 (CI: 0.91-0.94)** |
| 5-year | 0.70 (CI: 0.66-0.75) | 0.75 (CI: 0.71-0.78) | 0.67 (CI: 0.63-0.70) | 0.70 (CI: 0.66-0.75) | 0.76 (CI: 0.73-0.80) | 0.16 (CI: 0.12-0.19) | **0.91 (CI: 0.89-0.93)** |
| Average | 0.76 (CI: 0.73-0.79) | 0.71 (CI: 0.68-0.74) | 0.69 (CI: 0.67-0.72) | **0.76 (CI: 0.74-0.79)** | 0.73 (CI: 0.70-0.75) | 0.12 (CI: 0.09-0.14) | 0.73 (CI: 0.71-0.75) |

**Table S4M**: Sensitivity values with 95% Confidence Intervals (CI) rounded at 2 digits for XGBoost, GRU, Logistic Regression, LightGBM, LSTM, Naïve Bayes models and the clinical model in predicting clinical outcomes of the women population at different visit intervals over a 5-year term using the non-cumulative approach.

|  | **Prediction at 5 years term** | | | | | | |
| --- | --- | --- | --- | --- | --- | --- | --- |
| **Visit** | **XGboost** | **GRU** | **LogisticRegression** | **LightGBM** | **LSTM** | **Naïve Bayes** | **Clinical model** |
| Baseline | **0.78 (CI: 0.74-0.82)** | 0.63 (CI: 0.59-0.68) | 0.65 (CI: 0.61-0.69) | 0.76 (CI: 0.73-0.80) | 0.66 (CI: 0.61-0.71) | 0.03 (CI: 0.00-0.05) | 0.62 (CI: 0.57-0.67) |
| 1-year | 0.76 (CI: 0.73-0.80) | 0.80 (CI: 0.76-0.83) | 0.69 (CI: 0.65-0.73) | 0.76 (CI: 0.73-0.80) | **0.80 (CI: 0.77-0.83)** | 0.13 (CI: 0.09-0.16) | 0.75 (CI: 0.72-0.79) |
| 2-year | 0.73 (CI: 0.69-0.77) | **0.78 (CI: 0.74-0.81)** | 0.69 (CI: 0.65-0.73) | 0.71 (CI: 0.67-0.75) | **0.78 (CI: 0.74-0.81)** | 0.11 (CI: 0.07-0.14) | **0.78 (CI: 0.74-0.81)** |
| 3-year | 0.73 (CI: 0.68-0.79) | 0.76 (CI: 0.70-0.81) | 0.67 (CI: 0.62-0.72) | 0.75 (CI: 0.70-0.81) | 0.76 (CI: 0.71-0.81) | 0.17 (CI: 0.13-0.22) | **0.83 (CI: 0.80-0.86)** |
| 4-year | 0.78 (CI: 0.72-0.84) | 0.77 (CI: 0.72-0.82) | 0.71 (CI: 0.65-0.78) | 0.78 (CI: 0.72-0.84) | 0.75 (CI: 0.70-0.81) | 0.14 (CI: 0.09-0.19) | **0.91 (CI: 0.88-0.94)** |
| 5-year | 0.83 (CI: 0.78-0.89) | 0.84 (CI: 0.79-0.90) | 0.76 (CI: 0.70-0.83) | 0.87 (CI: 0.81-0.92) | 0.81 (CI: 0.75-0.87) | 0.02 (CI: -0.01-0.05) | **0.91 (CI: 0.87-0.95)** |
| Average | 0.77 (CI: 0.72-0.82) | 0.76 (CI: 0.72-0.81) | 0.70 (CI: 0.65-0.75) | 0.77 (CI: 0.73-0.82) | 0.76 (CI: 0.71-0.81) | 0.10 (CI: 0.06-0.13) | **0.80 (CI: 0.76-0.84)** |

**Table S4N**: Sensitivity values with 95% Confidence Intervals (CI) rounded at 2 digits for XGBoost, GRU, Logistic Regression, LightGBM, LSTM, Naïve Bayes models and the clinical model in predicting clinical outcomes of the women population at different visit intervals over a 2-year term using the non-cumulative approach.

|  | **Prediction at 2 years term** | | | | | | |
| --- | --- | --- | --- | --- | --- | --- | --- |
| **Visit** | **XGboost** | **GRU** | **LogisticRegression** | **LightGBM** | **LSTM** | **Naïve Bayes** | **Clinical model** |
| Baseline | 0.52 (CI: 0.46-0.58) | 0.32 (CI: 0.26-0.37) | 0.49 (CI: 0.43-0.56) | **0.53 (CI: 0.47-0.59)** | 0.38 (CI: 0.32-0.44) | 0.01 (CI: -0.01-0.02) | **0.53 (CI: 0.47-0.59)** |
| 1-year | 0.83 (CI: 0.80-0.86) | 0.64 (CI: 0.60-0.68) | 0.69 (CI: 0.65-0.73) | **0.83 (CI: 0.80-0.87)** | 0.67 (CI: 0.63-0.71) | 0.14 (CI: 0.10-0.18) | 0.79 (CI: 0.76-0.82) |
| 2-year | **0.79 (CI: 0.75-0.83)** | 0.72 (CI: 0.67-0.77) | 0.65 (CI: 0.60-0.70) | 0.76 (CI: 0.72-0.81) | 0.74 (CI: 0.69-0.79) | 0.14 (CI: 0.10-0.18) | 0.72 (CI: 0.68-0.76) |
| 3-year | 0.77 (CI: 0.73-0.83) | 0.72 (CI: 0.67-0.77) | 0.63 (CI: 0.58-0.68) | 0.77 (CI: 0.72-0.82) | 0.76 (CI: 0.71-0.81) | 0.12 (CI: 0.08-0.16) | **0.80 (CI: 0.76-0.83)** |
| 4-year | 0.77 (CI: 0.72-0.82) | 0.74 (CI: 0.70-0.79) | 0.52 (CI: 0.46-0.58) | 0.77 (CI: 0.72-0.82) | 0.79 (CI: 0.74-0.84) | 0.09 (CI: 0.05-0.12) | **0.92 (CI: 0.89-0.94)** |
| 5-year | 0.76 (CI: 0.70-0.82) | 0.79 (CI: 0.73-0.85) | 0.60 (CI: 0.53-0.67) | 0.77 (CI: 0.72-0.84) | 0.84 (CI: 0.79-0.90) | 0.02 (CI: -0.01-0.04) | **0.92 (CI: 0.89-0.95)** |
| Average | 0.74 (CI: 0.69-0.79) | 0.66 (CI: 0.61-0.71) | 0.60 (CI: 0.54-0.65) | 0.74 (CI: 0.69-0.79) | 0.70 (CI: 0.65-0.75) | 0.09 (CI: 0.06-0.11) | **0.78 (CI: 0.74-0.82)** |

**Table S4O**: Sensitivity values with 95% Confidence Intervals (CI) rounded at 2 digits for XGBoost, GRU, Logistic Regression, LightGBM, LSTM, Naïve Bayes models and the clinical model in predicting clinical outcomes of the men population at different visit intervals over a 5-year term using the cumulative approach.

|  | **Prediction at 5 years term** | | | | | | |
| --- | --- | --- | --- | --- | --- | --- | --- |
| **Visit** | **XGboost** | **GRU** | **LogisticRegression** | **LightGBM** | **LSTM** | **Naïve Bayes** | **Clinical model** |
| Baseline | 0.75 (CI: 0.73-0.77) | 0.66 (CI: 0.64-0.69) | 0.65 (CI: 0.63-0.67) | 0.74 (CI: 0.72-0.76) | 0.66 (CI: 0.63-0.68) | **0.86 (CI: 0.83-0.89)** | 0.60 (CI: 0.57-0.62) |
| 1-year | 0.80 (CI: 0.79-0.82) | **0.81 (CI: 0.79-0.83)** | 0.73 (CI: 0.71-0.75) | 0.80 (CI: 0.78-0.82) | 0.80 (CI: 0.78-0.82) | 0.29 (CI: 0.24-0.35) | 0.67 (CI: 0.65-0.69) |
| 2-year | **0.83 (CI: 0.81-0.85)** | 0.80 (CI: 0.77-0.82) | 0.80 (CI: 0.78-0.82) | **0.83 (CI: 0.81-0.85)** | 0.80 (CI: 0.78-0.83) | 0.55 (CI: 0.49-0.61) | 0.61 (CI: 0.58-0.64) |
| 3-year | 0.80 (CI: 0.78-0.83) | 0.79 (CI: 0.76-0.81) | 0.77 (CI: 0.75-0.80) | **0.81 (CI: 0.79-0.84)** | 0.80 (CI: 0.77-0.82) | 0.79 (CI: 0.77-0.82) | 0.66 (CI: 0.63-0.69) |
| 4-year | 0.76 (CI: 0.73-0.80) | 0.74 (CI: 0.71-0.77) | **0.79 (CI: 0.76-0.82)** | 0.75 (CI: 0.72-0.79) | 0.75 (CI: 0.71-0.78) | 0.75 (CI: 0.71-0.78) | 0.76 (CI: 0.73-0.79) |
| 5-year | 0.86 (CI: 0.83-0.89) | 0.71 (CI: 0.68-0.76) | **0.89 (CI: 0.86-0.91)** | 0.87 (CI: 0.84-0.90) | 0.72 (CI: 0.68-0.76) | 0.75 (CI: 0.71-0.79) | 0.80 (CI: 0.76-0.83) |
| Average | **0.80 (CI: 0.78-0.83)** | 0.75 (CI: 0.73-0.78) | 0.77 (CI: 0.75-0.80) | **0.80 (CI: 0.78-0.83)** | 0.76 (CI: 0.73-0.78) | 0.67 (CI: 0.63-0.71) | 0.68 (CI: 0.65-0.71) |

**Table S4P**: Sensitivity values with 95% Confidence Intervals (CI) rounded at 2 digits for XGBoost, GRU, Logistic Regression, LightGBM, LSTM, Naïve Bayes models and the clinical model in predicting clinical outcomes of the men population at different visit intervals over a 2-year term using the cumulative approach.

|  | **Prediction at 2 years term** | | | | | | |
| --- | --- | --- | --- | --- | --- | --- | --- |
| **Visit** | **XGboost** | **GRU** | **LogisticRegression** | **LightGBM** | **LSTM** | **Naïve Bayes** | **Clinical model** |
| Baseline | 0.39 (CI: 0.36-0.41) | 0.40 (CI: 0.37-0.43) | 0.40 (CI: 0.37-0.43) | 0.37 (CI: 0.34-0.39) | 0.42 (CI: 0.39-0.45) | **0.87 (CI: 0.85-0.90)** | 0.03 (CI: 0.02-0.04) |
| 1-year | **0.77 (CI: 0.75-0.79)** | 0.69 (CI: 0.67-0.71) | 0.74 (CI: 0.72-0.77) | 0.76 (CI: 0.74-0.79) | 0.70 (CI: 0.68-0.72) | 0.14 (CI: 0.11-0.16) | 0.53 (CI: 0.51-0.56) |
| 2-year | **0.83 (CI: 0.81-0.85)** | 0.78 (CI: 0.76-0.80) | 0.81 (CI: 0.79-0.83) | 0.81 (CI: 0.79-0.84) | 0.79 (CI: 0.77-0.82) | 0.20 (CI: 0.16-0.22) | 0.58 (CI: 0.55-0.60) |
| 3-year | 0.85 (CI: 0.84-0.87) | 0.82 (CI: 0.80-0.85) | 0.82 (CI: 0.80-0.85) | **0.87 (CI: 0.85-0.89)** | 0.84 (CI: 0.82-0.86) | 0.34 (CI: 0.30-0.38) | 0.68 (CI: 0.65-0.70) |
| 4-year | 0.87 (CI: 0.85-0.90) | 0.81 (CI: 0.79-0.84) | 0.82 (CI: 0.80-0.85) | **0.88 (CI: 0.86-0.90)** | 0.84 (CI: 0.82-0.87) | 0.52 (CI: 0.48-0.56) | 0.81 (CI: 0.78-0.84) |
| 5-year | 0.81 (CI: 0.77-0.84) | 0.75 (CI: 0.71-0.78) | 0.81 (CI: 0.78-0.85) | **0.82 (CI: 0.79-0.85)** | 0.76 (CI: 0.73-0.80) | 0.67 (CI: 0.63-0.71) | 0.78 (CI: 0.75-0.81) |
| Average | **0.75 (CI: 0.73-0.78)** | 0.71 (CI: 0.68-0.74) | 0.73 (CI: 0.71-0.76) | **0.75 (CI: 0.73-0.78)** | 0.73 (CI: 0.70-0.75) | 0.46 (CI: 0.42-0.49) | 0.57 (CI: 0.54-0.59) |

**Table S4Q**: Sensitivity values with 95% Confidence Intervals (CI) rounded at 2 digits for XGBoost, GRU, Logistic Regression, LightGBM, LSTM, Naïve Bayes models and the clinical model in predicting clinical outcomes of the women population at different visit intervals over a 5-year term using the cumulative approach.

|  | **Prediction at 5 years term** | | | | | | |
| --- | --- | --- | --- | --- | --- | --- | --- |
| **Visit** | **XGboost** | **GRU** | **LogisticRegression** | **LightGBM** | **LSTM** | **Naïve Bayes** | **Clinical model** |
| Baseline | 0.69 (CI: 0.64-0.73) | 0.63 (CI: 0.59-0.68) | 0.62 (CI: 0.57-0.66) | 0.70 (CI: 0.66-0.74) | 0.66 (CI: 0.61-0.71) | **0.83 (CI: 0.79-0.88)** | 0.68 (CI: 0.63-0.72) |
| 1-year | 0.73 (CI: 0.70-0.76) | 0.80 (CI: 0.76-0.83) | 0.73 (CI: 0.69-0.77) | 0.72 (CI: 0.68-0.76) | **0.80 (CI: 0.77-0.83)** | 0.29 (CI: 0.22-0.35) | 0.78 (CI: 0.75-0.81) |
| 2-year | 0.73 (CI: 0.69-0.77) | 0.78 (CI: 0.74-0.81) | 0.67 (CI: 0.63-0.72) | 0.72 (CI: 0.68-0.76) | 0.78 (CI: 0.74-0.81) | 0.46 (CI: 0.39-0.52) | **0.80 (CI: 0.76-0.84)** |
| 3-year | 0.70 (CI: 0.65-0.76) | 0.76 (CI: 0.70-0.81) | 0.67 (CI: 0.62-0.73) | 0.69 (CI: 0.64-0.75) | 0.76 (CI: 0.71-0.81) | 0.72 (CI: 0.66-0.77) | **0.82 (CI: 0.78-0.87)** |
| 4-year | 0.76 (CI: 0.70-0.82) | 0.77 (CI: 0.72-0.82) | 0.75 (CI: 0.70-0.81) | 0.74 (CI: 0.68-0.81) | 0.75 (CI: 0.70-0.81) | 0.79 (CI: 0.74-0.84) | **0.90 (CI: 0.86-0.95)** |
| 5-year | 0.85 (CI: 0.80-0.91) | 0.84 (CI: 0.79-0.90) | 0.77 (CI: 0.71-0.84) | 0.79 (CI: 0.73-0.85) | 0.81 (CI: 0.75-0.87) | 0.82 (CI: 0.77-0.88) | **0.94 (CI: 0.90-0.99)** |
| Average | 0.74 (CI: 0.70-0.79) | 0.76 (CI: 0.72-0.81) | 0.70 (CI: 0.65-0.76) | 0.73 (CI: 0.68-0.78) | 0.76 (CI: 0.71-0.81) | 0.65 (CI: 0.60-0.71) | **0.82 (CI: 0.78-0.86)** |

**Table S4R**: Sensitivity values with 95% Confidence Intervals (CI) rounded at 2 digits for XGBoost, GRU, Logistic Regression, LightGBM, LSTM, Naïve Bayes models and the clinical model in predicting clinical outcomes of the women population at different visit intervals over a 2-year term using the cumulative approach.

|  | **Prediction at 2 years term** | | | | | | |
| --- | --- | --- | --- | --- | --- | --- | --- |
| **Visit** | **XGboost** | **GRU** | **LogisticRegression** | **LightGBM** | **LSTM** | **Naïve Bayes** | **Clinical model** |
| Baseline | 0.30 (CI: 0.25-0.36) | 0.32 (CI: 0.26-0.37) | 0.35 (CI: 0.29-0.41) | 0.29 (CI: 0.23-0.35) | 0.38 (CI: 0.32-0.44) | **0.76 (CI: 0.71-0.82)** | 0.00 (CI: -0.00-0.01) |
| 1-year | 0.75 (CI: 0.71-0.78) | 0.64 (CI: 0.60-0.68) | 0.64 (CI: 0.59-0.68) | **0.75 (CI: 0.71-0.79)** | 0.67 (CI: 0.63-0.71) | 0.21 (CI: 0.17-0.25) | 0.71 (CI: 0.67-0.75) |
| 2-year | 0.76 (CI: 0.72-0.81) | 0.72 (CI: 0.67-0.77) | 0.67 (CI: 0.62-0.72) | 0.75 (CI: 0.71-0.79) | 0.74 (CI: 0.69-0.79) | 0.19 (CI: 0.14-0.23) | **0.84 (CI: 0.80-0.88)** |
| 3-year | 0.79 (CI: 0.75-0.84) | 0.72 (CI: 0.67-0.77) | 0.72 (CI: 0.67-0.77) | 0.79 (CI: 0.74-0.84) | 0.76 (CI: 0.71-0.81) | 0.21 (CI: 0.16-0.26) | **0.91 (CI: 0.88-0.95)** |
| 4-year | 0.81 (CI: 0.76-0.86) | 0.74 (CI: 0.70-0.79) | 0.75 (CI: 0.70-0.80) | 0.82 (CI: 0.77-0.86) | 0.79 (CI: 0.74-0.84) | 0.57 (CI: 0.51-0.62) | **0.95 (CI: 0.93-0.98)** |
| 5-year | 0.78 (CI: 0.73-0.84) | 0.79 (CI: 0.73-0.85) | 0.78 (CI: 0.72-0.84) | 0.80 (CI: 0.75-0.86) | 0.84 (CI: 0.79-0.90) | 0.70 (CI: 0.64-0.77) | **0.90 (CI: 0.86-0.95)** |
| Average | 0.70 (CI: 0.65-0.75) | 0.66 (CI: 0.61-0.71) | 0.65 (CI: 0.60-0.70) | 0.70 (CI: 0.65-0.75) | 0.70 (CI: 0.65-0.75) | 0.44 (CI: 0.39-0.49) | **0.72 (CI: 0.69-0.75)** |

**Table S4S**: Sensitivity values with 95% Confidence Intervals (CI) rounded at 2 digits for XGBoost, GRU, Logistic Regression, LightGBM, LSTM, Naïve Bayes models and the clinical model in predicting clinical outcomes of the bicuspid population at different visit intervals over a 5-year term using the non-cumulative approach.

|  | **Prediction at 5 years term** | | | | | | |
| --- | --- | --- | --- | --- | --- | --- | --- |
| **Visit** | **XGboost** | **GRU** | **LogisticRegression** | **LightGBM** | **LSTM** | **Naïve Bayes** | **Clinical model** |
| Baseline | **0.81 (CI: 0.79-0.83)** | 0.71 (CI: 0.69-0.74) | 0.72 (CI: 0.69-0.74) | 0.80 (CI: 0.78-0.82) | 0.72 (CI: 0.69-0.74) | 0.05 (CI: 0.02-0.06) | 0.57 (CI: 0.55-0.60) |
| 1-year | 0.82 (CI: 0.80-0.84) | **0.83 (CI: 0.81-0.85)** | 0.75 (CI: 0.73-0.77) | 0.82 (CI: 0.80-0.84) | **0.83 (CI: 0.81-0.85)** | 0.12 (CI: 0.09-0.14) | 0.72 (CI: 0.70-0.75) |
| 2-year | **0.83 (CI: 0.81-0.85)** | 0.81 (CI: 0.79-0.83) | 0.75 (CI: 0.73-0.77) | **0.83 (CI: 0.81-0.85)** | 0.81 (CI: 0.79-0.84) | 0.13 (CI: 0.11-0.16) | 0.77 (CI: 0.75-0.79) |
| 3-year | 0.82 (CI: 0.80-0.84) | 0.80 (CI: 0.77-0.82) | 0.74 (CI: 0.72-0.77) | **0.84 (CI: 0.82-0.86)** | 0.81 (CI: 0.78-0.83) | 0.18 (CI: 0.15-0.21) | 0.81 (CI: 0.79-0.83) |
| 4-year | 0.76 (CI: 0.73-0.79) | 0.76 (CI: 0.73-0.80) | 0.69 (CI: 0.66-0.72) | 0.75 (CI: 0.71-0.78) | 0.77 (CI: 0.73-0.80) | 0.12 (CI: 0.09-0.14) | **0.91 (CI: 0.89-0.93)** |
| 5-year | 0.70 (CI: 0.66-0.75) | 0.76 (CI: 0.72-0.80) | 0.72 (CI: 0.67-0.76) | 0.72 (CI: 0.68-0.76) | 0.77 (CI: 0.73-0.81) | 0.07 (CI: 0.05-0.10) | **0.92 (CI: 0.90-0.94)** |
| Average | **0.79 (CI: 0.77-0.82)** | 0.78 (CI: 0.75-0.81) | 0.73 (CI: 0.70-0.76) | **0.79 (CI: 0.77-0.82)** | 0.79 (CI: 0.76-0.81) | 0.11 (CI: 0.09-0.14) | 0.78 (CI: 0.76-0.81) |

**Table S4T**: Sensitivity values with 95% Confidence Intervals (CI) rounded at 2 digits for XGBoost, GRU, Logistic Regression, LightGBM, LSTM, Naïve Bayes models and the clinical model in predicting clinical outcomes of the bicuspid population at different visit intervals over a 2-year term using the non-cumulative approach.

|  | **Prediction at 2 years term** | | | | | | |
| --- | --- | --- | --- | --- | --- | --- | --- |
| **Visit** | **XGboost** | **GRU** | **LogisticRegression** | **LightGBM** | **LSTM** | **Naïve Bayes** | **Clinical model** |
| Baseline | 0.63 (CI: 0.60-0.66) | 0.44 (CI: 0.41-0.47) | 0.58 (CI: 0.56-0.61) | **0.66 (CI: 0.63-0.69)** | 0.48 (CI: 0.45-0.51) | 0.02 (CI: -0.00-0.04) | 0.55 (CI: 0.52-0.58) |
| 1-year | 0.82 (CI: 0.80-0.84) | 0.70 (CI: 0.67-0.72) | 0.71 (CI: 0.69-0.74) | **0.84 (CI: 0.82-0.86)** | 0.71 (CI: 0.68-0.73) | 0.12 (CI: 0.09-0.14) | 0.62 (CI: 0.60-0.65) |
| 2-year | **0.84 (CI: 0.82-0.86)** | 0.80 (CI: 0.78-0.82) | 0.70 (CI: 0.68-0.73) | 0.83 (CI: 0.81-0.85) | 0.82 (CI: 0.80-0.85) | 0.14 (CI: 0.11-0.16) | 0.71 (CI: 0.69-0.74) |
| 3-year | **0.85 (CI: 0.83-0.87)** | 0.83 (CI: 0.81-0.85) | 0.76 (CI: 0.74-0.79) | **0.85 (CI: 0.83-0.87)** | 0.84 (CI: 0.82-0.87) | 0.16 (CI: 0.13-0.18) | 0.79 (CI: 0.77-0.81) |
| 4-year | 0.86 (CI: 0.84-0.89) | 0.83 (CI: 0.80-0.85) | 0.72 (CI: 0.68-0.75) | 0.87 (CI: 0.84-0.89) | 0.86 (CI: 0.83-0.88) | 0.13 (CI: 0.10-0.16) | **0.92 (CI: 0.90-0.94)** |
| 5-year | 0.72 (CI: 0.68-0.77) | 0.75 (CI: 0.71-0.79) | 0.64 (CI: 0.60-0.69) | 0.73 (CI: 0.68-0.77) | 0.77 (CI: 0.73-0.81) | 0.07 (CI: 0.04-0.10) | **0.90 (CI: 0.88-0.92)** |
| Average | 0.79 (CI: 0.76-0.82) | 0.73 (CI: 0.70-0.75) | 0.69 (CI: 0.66-0.72) | **0.80 (CI: 0.77-0.82)** | 0.75 (CI: 0.72-0.78) | 0.11 (CI: 0.08-0.12) | 0.75 (CI: 0.73-0.77) |

**Table S4U**: Sensitivity values with 95% Confidence Intervals (CI) rounded at 2 digits for XGBoost, GRU, Logistic Regression, LightGBM, LSTM, Naïve Bayes models and the clinical model in predicting clinical outcomes of the tricuspid population at different visit intervals over a 5-year term using the non-cumulative approach.

|  | **Prediction at 5 years term** | | | | | | |
| --- | --- | --- | --- | --- | --- | --- | --- |
| **Visit** | **XGboost** | **GRU** | **LogisticRegression** | **LightGBM** | **LSTM** | **Naïve Bayes** | **Clinical model** |
| Baseline | **0.63 (CI: 0.59-0.68)** | 0.47 (CI: 0.42-0.52) | **0.63 (CI: 0.58-0.68)** | 0.61 (CI: 0.56-0.67) | 0.46 (CI: 0.41-0.52) | 0.03 (CI: -0.00-0.05) | 0.56 (CI: 0.52-0.62) |
| 1-year | **0.71 (CI: 0.67-0.76)** | 0.67 (CI: 0.62-0.71) | 0.70 (CI: 0.65-0.74) | 0.71 (CI: 0.66-0.75) | 0.67 (CI: 0.62-0.72) | 0.17 (CI: 0.13-0.20) | 0.66 (CI: 0.62-0.71) |
| 2-year | 0.76 (CI: 0.71-0.80) | 0.73 (CI: 0.68-0.78) | 0.73 (CI: 0.69-0.78) | 0.74 (CI: 0.69-0.79) | 0.74 (CI: 0.69-0.78) | 0.11 (CI: 0.08-0.15) | **0.82 (CI: 0.78-0.85)** |
| 3-year | 0.69 (CI: 0.63-0.76) | 0.66 (CI: 0.59-0.73) | 0.64 (CI: 0.57-0.70) | 0.68 (CI: 0.62-0.75) | 0.67 (CI: 0.60-0.74) | 0.08 (CI: 0.04-0.12) | **0.81 (CI: 0.77-0.86)** |
| 4-year | 0.63 (CI: 0.55-0.71) | 0.57 (CI: 0.49-0.65) | 0.52 (CI: 0.44-0.60) | 0.63 (CI: 0.56-0.72) | 0.55 (CI: 0.46-0.63) | 0.01 (CI: -0.01-0.01) | **0.85 (CI: 0.80-0.90)** |
| 5-year | 0.58 (CI: 0.50-0.67) | 0.60 (CI: 0.52-0.68) | 0.53 (CI: 0.45-0.61) | 0.61 (CI: 0.52-0.69) | 0.60 (CI: 0.52-0.69) | 0.04 (CI: 0.01-0.06) | **0.90 (CI: 0.86-0.95)** |
| Average | 0.67 (CI: 0.61-0.73) | 0.62 (CI: 0.55-0.68) | 0.63 (CI: 0.56-0.69) | 0.66 (CI: 0.60-0.73) | 0.62 (CI: 0.55-0.68) | 0.07 (CI: 0.04-0.09) | **0.77 (CI: 0.73-0.82)** |

**Table S4V**: Sensitivity values with 95% Confidence Intervals (CI) rounded at 2 digits for XGBoost, GRU, Logistic Regression, LightGBM, LSTM, Naïve Bayes models and the clinical model in predicting clinical outcomes of the tricuspid population at different visit intervals over a 2-year term using the non-cumulative approach.

|  | **Prediction at 2 years term** | | | | | | |
| --- | --- | --- | --- | --- | --- | --- | --- |
| **Visit** | **XGboost** | **GRU** | **LogisticRegression** | **LightGBM** | **LSTM** | **Naïve Bayes** | **Clinical model** |
| Baseline | 0.41 (CI: 0.35-0.46) | 0.20 (CI: 0.16-0.24) | **0.43 (CI: 0.38-0.49)** | 0.38 (CI: 0.33-0.44) | 0.21 (CI: 0.17-0.25) | 0.03 (CI: 0.00-0.05) | 0.33 (CI: 0.27-0.38) |
| 1-year | 0.65 (CI: 0.60-0.70) | 0.52 (CI: 0.47-0.57) | 0.62 (CI: 0.57-0.67) | **0.66 (CI: 0.62-0.71)** | 0.56 (CI: 0.51-0.61) | 0.16 (CI: 0.12-0.20) | 0.45 (CI: 0.40-0.51) |
| 2-year | 0.71 (CI: 0.66-0.75) | 0.69 (CI: 0.64-0.74) | 0.69 (CI: 0.65-0.74) | 0.70 (CI: 0.65-0.74) | 0.70 (CI: 0.65-0.75) | 0.12 (CI: 0.08-0.15) | **0.76 (CI: 0.72-0.80)** |
| 3-year | 0.67 (CI: 0.62-0.73) | 0.65 (CI: 0.59-0.71) | 0.55 (CI: 0.49-0.61) | 0.65 (CI: 0.60-0.71) | 0.67 (CI: 0.61-0.73) | 0.07 (CI: 0.04-0.09) | **0.78 (CI: 0.74-0.83)** |
| 4-year | 0.79 (CI: 0.73-0.85) | 0.68 (CI: 0.61-0.75) | 0.50 (CI: 0.43-0.57) | 0.77 (CI: 0.71-0.84) | 0.71 (CI: 0.65-0.78) | 0.02 (CI: 0.00-0.03) | **0.88 (CI: 0.84-0.94)** |
| 5-year | 0.60 (CI: 0.52-0.68) | 0.67 (CI: 0.59-0.74) | 0.54 (CI: 0.45-0.62) | 0.55 (CI: 0.47-0.63) | 0.70 (CI: 0.63-0.77) | 0.01 (CI: -0.00-0.02) | **0.89 (CI: 0.84-0.94)** |
| Average | 0.64 (CI: 0.58-0.70) | 0.57 (CI: 0.51-0.63) | 0.56 (CI: 0.50-0.62) | 0.62 (CI: 0.56-0.68) | 0.59 (CI: 0.54-0.65) | 0.07 (CI: 0.04-0.09) | **0.68 (CI: 0.64-0.73)** |

**Table S4W**: Sensitivity values with 95% Confidence Intervals (CI) rounded at 2 digits for XGBoost, GRU, Logistic Regression, LightGBM, LSTM, Naïve Bayes models and the clinical model in predicting clinical outcomes of the bicuspid population at different visit intervals over a 5-year term using the cumulative approach.

|  | **Prediction at 5 years term** | | | | | | |
| --- | --- | --- | --- | --- | --- | --- | --- |
| **Visit** | **XGboost** | **GRU** | **LogisticRegression** | **LightGBM** | **LSTM** | **Naïve Bayes** | **Clinical model** |
| Baseline | 0.40 (CI: 0.37-0.43) | 0.44 (CI: 0.41-0.47) | 0.40 (CI: 0.37-0.43) | 0.38 (CI: 0.35-0.41) | 0.48 (CI: 0.45-0.51) | **0.91 (CI: 0.89-0.93)** | 0.03 (CI: 0.02-0.04) |
| 1-year | **0.80 (CI: 0.78-0.82)** | 0.70 (CI: 0.67-0.72) | 0.73 (CI: 0.70-0.75) | 0.80 (CI: 0.77-0.82) | 0.71 (CI: 0.68-0.73) | 0.14 (CI: 0.11-0.17) | 0.59 (CI: 0.56-0.61) |
| 2-year | **0.84 (CI: 0.82-0.87)** | 0.80 (CI: 0.78-0.82) | 0.80 (CI: 0.78-0.82) | 0.84 (CI: 0.82-0.86) | 0.82 (CI: 0.80-0.85) | 0.17 (CI: 0.14-0.20) | 0.64 (CI: 0.62-0.67) |
| 3-year | 0.83 (CI: 0.81-0.86) | 0.83 (CI: 0.81-0.85) | 0.80 (CI: 0.77-0.82) | **0.85 (CI: 0.83-0.87)** | 0.84 (CI: 0.82-0.87) | 0.36 (CI: 0.32-0.40) | 0.74 (CI: 0.72-0.77) |
| 4-year | **0.88 (CI: 0.86-0.90)** | 0.83 (CI: 0.80-0.85) | 0.83 (CI: 0.80-0.86) | **0.88 (CI: 0.86-0.90)** | 0.86 (CI: 0.83-0.88) | 0.54 (CI: 0.51-0.58) | 0.84 (CI: 0.81-0.87) |
| 5-year | 0.78 (CI: 0.74-0.82) | 0.75 (CI: 0.71-0.79) | 0.79 (CI: 0.75-0.83) | 0.80 (CI: 0.77-0.85) | 0.77 (CI: 0.73-0.81) | 0.65 (CI: 0.60-0.70) | **0.81 (CI: 0.77-0.85)** |
| Average | 0.76 (CI: 0.73-0.78) | 0.73 (CI: 0.70-0.75) | 0.73 (CI: 0.70-0.75) | **0.76 (CI: 0.73-0.79)** | 0.75 (CI: 0.72-0.78) | 0.46 (CI: 0.43-0.50) | 0.61 (CI: 0.58-0.64) |

**Table S4X**: Sensitivity values with 95% Confidence Intervals (CI) rounded at 2 digits for XGBoost, GRU, Logistic Regression, LightGBM, LSTM, Naïve Bayes models and the clinical model in predicting clinical outcomes of the bicuspid population at different visit intervals over a 2-year term using the cumulative approach.

|  | **Prediction at 2 years term** | | | | | | |
| --- | --- | --- | --- | --- | --- | --- | --- |
| **Visit** | **XGboost** | **GRU** | **LogisticRegression** | **LightGBM** | **LSTM** | **Naïve Bayes** | **Clinical model** |
| Baseline | 0.77 (CI: 0.75-0.79) | 0.71 (CI: 0.69-0.74) | 0.66 (CI: 0.64-0.68) | 0.75 (CI: 0.73-0.77) | 0.72 (CI: 0.69-0.74) | **0.88 (CI: 0.85-0.92)** | 0.64 (CI: 0.62-0.66) |
| 1-year | 0.80 (CI: 0.79-0.82) | **0.83 (CI: 0.81-0.85)** | 0.76 (CI: 0.74-0.78) | 0.80 (CI: 0.78-0.82) | **0.83 (CI: 0.81-0.85)** | 0.29 (CI: 0.23-0.35) | 0.71 (CI: 0.68-0.73) |
| 2-year | **0.83 (CI: 0.81-0.85)** | 0.81 (CI: 0.79-0.83) | 0.77 (CI: 0.74-0.80) | **0.83 (CI: 0.81-0.85)** | 0.81 (CI: 0.79-0.84) | 0.53 (CI: 0.47-0.59) | 0.64 (CI: 0.61-0.67) |
| 3-year | 0.80 (CI: 0.77-0.82) | 0.80 (CI: 0.77-0.82) | 0.76 (CI: 0.73-0.78) | **0.81 (CI: 0.78-0.83)** | **0.81 (CI: 0.78-0.83)** | 0.78 (CI: 0.75-0.81) | 0.69 (CI: 0.66-0.72) |
| 4-year | 0.78 (CI: 0.75-0.82) | 0.76 (CI: 0.73-0.80) | 0.76 (CI: 0.73-0.80) | **0.79 (CI: 0.75-0.82)** | 0.77 (CI: 0.73-0.80) | 0.74 (CI: 0.71-0.77) | 0.76 (CI: 0.73-0.80) |
| 5-year | 0.86 (CI: 0.83-0.89) | 0.76 (CI: 0.72-0.80) | **0.87 (CI: 0.84-0.90)** | **0.87 (CI: 0.84-0.90)** | 0.77 (CI: 0.73-0.81) | 0.74 (CI: 0.69-0.78) | 0.84 (CI: 0.81-0.88) |
| Average | **0.81 (CI: 0.78-0.83)** | 0.78 (CI: 0.75-0.81) | 0.76 (CI: 0.74-0.79) | **0.81 (CI: 0.78-0.83)** | 0.79 (CI: 0.76-0.81) | 0.66 (CI: 0.62-0.70) | 0.71 (CI: 0.69-0.74) |

**Table S4Y**: Sensitivity values with 95% Confidence Intervals (CI) rounded at 2 digits for XGBoost, GRU, Logistic Regression, LightGBM, LSTM, Naïve Bayes models and the clinical model in predicting clinical outcomes of the tricuspid population at different visit intervals over a 5-year term using the cumulative approach.

|  | **Prediction at 5 years term** | | | | | | |
| --- | --- | --- | --- | --- | --- | --- | --- |
| **Visit** | **XGboost** | **GRU** | **LogisticRegression** | **LightGBM** | **LSTM** | **Naïve Bayes** | **Clinical model** |
| Baseline | 0.31 (CI: 0.26-0.35) | 0.20 (CI: 0.16-0.24) | 0.36 (CI: 0.30-0.41) | 0.30 (CI: 0.25-0.35) | 0.21 (CI: 0.17-0.25) | **0.75 (CI: 0.70-0.80)** | 0.01 (CI: 0.00-0.02) |
| 1-year | 0.54 (CI: 0.49-0.59) | 0.52 (CI: 0.47-0.57) | **0.62 (CI: 0.57-0.68)** | 0.56 (CI: 0.51-0.61) | 0.56 (CI: 0.51-0.61) | 0.15 (CI: 0.11-0.19) | 0.43 (CI: 0.38-0.48) |
| 2-year | 0.72 (CI: 0.67-0.76) | 0.69 (CI: 0.64-0.74) | **0.75 (CI: 0.71-0.80)** | 0.70 (CI: 0.65-0.75) | 0.70 (CI: 0.65-0.75) | 0.22 (CI: 0.18-0.26) | 0.62 (CI: 0.58-0.66) |
| 3-year | 0.77 (CI: 0.71-0.82) | 0.65 (CI: 0.59-0.71) | 0.74 (CI: 0.69-0.80) | **0.79 (CI: 0.74-0.85)** | 0.67 (CI: 0.61-0.73) | 0.20 (CI: 0.14-0.25) | 0.66 (CI: 0.61-0.72) |
| 4-year | 0.81 (CI: 0.75-0.87) | 0.68 (CI: 0.61-0.75) | 0.73 (CI: 0.66-0.79) | **0.82 (CI: 0.76-0.88)** | 0.71 (CI: 0.65-0.78) | 0.47 (CI: 0.39-0.54) | 0.79 (CI: 0.73-0.85) |
| 5-year | 0.67 (CI: 0.60-0.75) | 0.67 (CI: 0.59-0.74) | **0.71 (CI: 0.64-0.79)** | 0.68 (CI: 0.60-0.76) | 0.70 (CI: 0.63-0.77) | 0.69 (CI: 0.61-0.77) | 0.71 (CI: 0.64-0.78) |
| Average | 0.64 (CI: 0.58-0.69) | 0.57 (CI: 0.51-0.63) | **0.65 (CI: 0.60-0.71)** | 0.64 (CI: 0.59-0.70) | 0.59 (CI: 0.54-0.65) | 0.41 (CI: 0.36-0.47) | 0.54 (CI: 0.49-0.59) |

**Table S4Z**: Sensitivity values with 95% Confidence Intervals (CI) rounded at 2 digits for XGBoost, GRU, Logistic Regression, LightGBM, LSTM, Naïve Bayes models and the clinical model in predicting clinical outcomes of the tricuspid population at different visit intervals over a 2-year term using the cumulative approach.

|  | **Prediction at 2 years term** | | | | | | |
| --- | --- | --- | --- | --- | --- | --- | --- |
| **Visit** | **XGboost** | **GRU** | **LogisticRegression** | **LightGBM** | **LSTM** | **Naïve Bayes** | **Clinical model** |
| Baseline | 0.63 (CI: 0.58-0.68) | 0.47 (CI: 0.42-0.52) | 0.60 (CI: 0.55-0.66) | 0.65 (CI: 0.60-0.70) | 0.46 (CI: 0.41-0.52) | **0.78 (CI: 0.73-0.84)** | 0.59 (CI: 0.54-0.64) |
| 1-year | **0.72 (CI: 0.68-0.77)** | 0.67 (CI: 0.62-0.71) | 0.61 (CI: 0.57-0.66) | 0.71 (CI: 0.66-0.75) | 0.67 (CI: 0.62-0.72) | 0.25 (CI: 0.20-0.31) | 0.58 (CI: 0.53-0.63) |
| 2-year | 0.80 (CI: 0.76-0.84) | 0.73 (CI: 0.68-0.78) | 0.75 (CI: 0.70-0.79) | **0.80 (CI: 0.76-0.85)** | 0.74 (CI: 0.69-0.78) | 0.51 (CI: 0.44-0.59) | 0.63 (CI: 0.58-0.68) |
| 3-year | 0.68 (CI: 0.62-0.75) | 0.66 (CI: 0.59-0.73) | 0.69 (CI: 0.63-0.76) | 0.67 (CI: 0.60-0.74) | 0.67 (CI: 0.60-0.74) | 0.66 (CI: 0.59-0.73) | **0.70 (CI: 0.64-0.77)** |
| 4-year | 0.63 (CI: 0.54-0.71) | 0.57 (CI: 0.49-0.65) | 0.67 (CI: 0.59-0.75) | 0.59 (CI: 0.51-0.68) | 0.55 (CI: 0.46-0.63) | **0.71 (CI: 0.63-0.79)** | 0.67 (CI: 0.59-0.75) |
| 5-year | **0.74 (CI: 0.67-0.82)** | 0.60 (CI: 0.52-0.68) | **0.74 (CI: 0.67-0.82)** | 0.68 (CI: 0.60-0.76) | 0.60 (CI: 0.52-0.69) | 0.70 (CI: 0.62-0.78) | 0.69 (CI: 0.62-0.77) |
| Average | **0.70 (CI: 0.64-0.76)** | 0.62 (CI: 0.55-0.68) | 0.68 (CI: 0.62-0.74) | 0.68 (CI: 0.62-0.75) | 0.62 (CI: 0.55-0.68) | 0.60 (CI: 0.54-0.67) | 0.64 (CI: 0.58-0.71) |
